# Using Genomic Information to Guide Ibrutinib Treatment Decisions in Chronic Lymphocytic Leukaemia: A Cost-Effectiveness Analysis

**DOI:** 10.1007/s40273-017-0519-z

**Published:** 2017-07-31

**Authors:** James Buchanan, Sarah Wordsworth, Ruth Clifford, Pauline Robbe, Jenny C. Taylor, Anna Schuh, Samantha J. L. Knight

**Affiliations:** 1 Health Economics Research Centre, Nuffield Department of Population Health, University of Oxford, Old Road Campus, Headington, Oxford, OX3 7LF UK; 2 Oxford Molecular Diagnostics Centre, Oxford University Hospitals Trust, Oxford, UK; 3 Oxford Cancer and Haematology Centre, Churchill Hospital, Oxford, UK; 4 National Institute for Health Research Oxford Biomedical Research Centre, Oxford, UK; 5 Wellcome Trust Centre for Human Genetics, University of Oxford, Oxford, UK; 6 Department of Oncology, University of Oxford, Oxford, UK

## Abstract

**Background:**

Genomic tests may improve the stratification of patients to receive new therapies in several disease areas. However, the use of expensive targeted therapies can impact on the cost effectiveness of these tests. This study presents an economic evaluation of genomic testing in chronic lymphocytic leukaemia in the context of the UK National Health Service.

**Methods:**

Cost-effectiveness and cost-utility analyses (using life-years and quality-adjusted life-years) were undertaken from a National Health Service and societal perspective. Five strategies were evaluated across several age groups using Markov modelling: three strategies that reflected varying current genetic testing practice and two configurations of genomic testing (including ibrutinib treatment).

**Results:**

Genomic testing strategies yielded the most life-years/quality-adjusted life-years per patient, but were not cost effective compared with a threshold of £30,000 per life-year/quality-adjusted life-year gained. Cost-effectiveness acceptability curves indicated that there was some uncertainty surrounding this result. A genomic testing strategy becomes the most cost-effective option if a higher end-of-life cost-effectiveness threshold of £50,000 is applied, if a societal costing perspective is considered in 25-year-old patients or if the cost of ibrutinib treatment falls.

**Conclusion:**

Stratifying patients with chronic lymphocytic leukaemia to targeted treatment using genomic testing improves health outcomes, but will likely only represent a cost-effective use of limited National Health Service resources if a higher cost-effectiveness threshold or societal costing perspective is applied, or if the price of ibrutinib treatment is reduced. This result may be broadly indicative of the likely cost effectiveness of other genomic tests that inform the stratification of patients to high cost-targeted therapies.

**Electronic supplementary material:**

The online version of this article (doi:10.1007/s40273-017-0519-z) contains supplementary material, which is available to authorized users.

## Key Points

**Table:** 

Stratifying patients with chronic lymphocytic leukaemia to targeted treatment using genomic testing is not a cost-effective use of limited National Health Service resources, primarily owing to the high cost of ibrutinib treatment.
However, if a higher end-of-life cost-effectiveness threshold is applied, if a societal costing perspective is considered in younger patients or if the cost of ibrutinib treatment falls, strategies that use genomic information to stratify patients to ibrutinib treatment become cost effective.

## Introduction

Genetic tests are assays targeted solely at specific genes of interest, and the use of these tests to guide diagnosis, prognosis and treatment decisions is now routine practice in several clinical areas (e.g. *BRCA1/2* testing in breast cancer [1]). In some clinical contexts, attention is now turning towards genomic interventions that could improve disease stratification and permit the more widespread use of individually tailored therapies, facilitating a precision medicine approach to patient care [2]. These next-generation sequencing (NGS) technologies, which include targeted gene panels, whole-exome sequencing and whole-genome sequencing, differ from genetic tests in that they offer genome-wide testing capability, simultaneously scrutinising multiple genes and their inter-relationships to identify their combined influence on diseases and disorders [3]. These tests may provide information that could improve the quantity and quality of life of patients, and evidence of the clinical utility of such tests is already emerging [4–9].

In many countries, the translation of these genomic technologies from research settings into clinical practice will be informed by evidence of both clinical utility and cost effectiveness. However, health economists face a variety of methodological challenges when conducting economic evaluations of genomic technologies. These challenges are not necessarily unique to the evaluation of genomic technologies, but the quantity and range of challenges are such that economic evaluations of genomic technologies present a particular challenge for health economists. These challenges include selecting an appropriate analytical approach and uncertainty surrounding the appropriate measurement of outcomes [10]. A key challenge is that economic evaluations of genomic technologies must include the costs and consequences associated with actions taken on the basis of these test results. Importantly, this includes the cost of any targeted therapy. Such therapies are often much more expensive than the genomic tests that could be used to guide treatment selection, and evidence from the genetic testing literature suggests that in such a scenario these interventions may not be cost effective [11, 12]. Given these challenges, few good-quality economic evaluations of genomic technologies have been conducted (compared with other types of diagnostic tests), and there is limited evidence available to inform the allocation (or not) of scarce healthcare resources to genomic testing.

Chronic lymphocytic leukaemia (CLL) is one disease in which genomic testing could generate clinical utility by stratifying patients to targeted therapies. Chronic lymphocytic leukaemia is the most common adult leukaemia in the Western world [13] and chemotherapy is usually offered to patients with symptomatic disease. First-line treatment with fludarabine, cyclophosphamide and rituximab (FCR) combination therapy is the standard of care. However, 25% of patients either do not respond to FCR or will relapse within 2 years of achieving remission [14, 15]. Genetic factors (e.g. *TP53* mutations) are thought to be the main drivers of treatment resistance [13, 16], and current UK and international guidelines recommend that patients undergo pre-treatment genetic testing to guide treatment selection [17–19]. Two tests targeted specifically at *TP53* mutations are commonly used: fluorescent in situ hybridisation testing and Sanger sequencing. However, these low-resolution tests can only identify around a third of FCR non-responders [20].

New testing approaches such as targeted NGS offer a whole genome view at increased resolution, providing additional information on multiple genetic alterations with clinical utility in CLL [15, 21, 22]. This information could help to identify more FCR non-responders, further reducing ineffective and therefore unnecessary treatment and associated side effects such as anaemia and pneumonia [20, 23]. However, these new tests are yet to be translated into clinical practice. While there is ample evidence that this genomic information is clinically useful [15, 16, 21, 22, 24–29], little data are available on the costs and benefits of such testing in CLL.

One possible consequence of a ‘non-response’ genomic test result for patients with CLL could be the use of new therapies such as ibrutinib. Ibrutinib may improve progression-free survival, overall survival and response rate amongst patients with CLL [30], and may also yield productivity gains, allowing patients to return to work more quickly, even if they are still undergoing treatment. However, ibrutinib is expensive (the annual cost of treatment is £56,000) [31, 32], hence its use could impact on the cost effectiveness of genomic testing in this context. In addition, there is currently limited evidence on the costs and health outcomes associated with adding ibrutinib to standard clinical pathways.

This article presents an economic evaluation of the use of genomic testing to guide CLL treatment decisions in the UK National Health Service (NHS). The main aim was to compare current genetic testing practice with different configurations of future genomic testing practice and ibrutinib treatment, in terms of costs, health outcomes and cost effectiveness. The conditions under which genomic tests could stratify patients to ibrutinib treatment in a cost-effective manner are also explored, including the application of a societal analytical perspective in different patient subgroups.

## Methods

Given the uncertainty surrounding the appropriate measurement of outcomes in genomics, two forms of economic evaluation were conducted, cost-effectiveness analysis (CEA) and cost-utility analysis (CUA), with outcomes measured using life-years and quality-adjusted life-years (QALYs). A decision analytic model was constructed that synthesised data on the clinical utility of genetic and genomic testing from a retrospective study [21] with data on healthcare resource use, health outcomes and costs from secondary sources. This model is summarised below and reported in full in the appendices contained within the Electronic Supplementary Material (ESM).

### Patient Population and Clinical Pathways

Clinical pathways for patients with CLL in the UK NHS were developed via interviews with haematologists at the Oxford University Hospitals NHS Trust (hereafter OUH), Royal Liverpool University Hospital, Royal Marsden Hospital (London) and The Christie NHS Foundation Trust (Manchester). These pathways focused on ‘go-go’ patients. These patients represent around 40% of all patients diagnosed with CLL and are judged to be physically fit and able to tolerate aggressive chemotherapy treatment, based on factors such as age, presence of co-morbidities and susceptibility to infection [18]. As genetic information can inform the clinical management of these patients, and the retrospective study primarily considered patients with these characteristics, this economic evaluation focused on this patient group.

### Model Structure

**Fig. 1:**
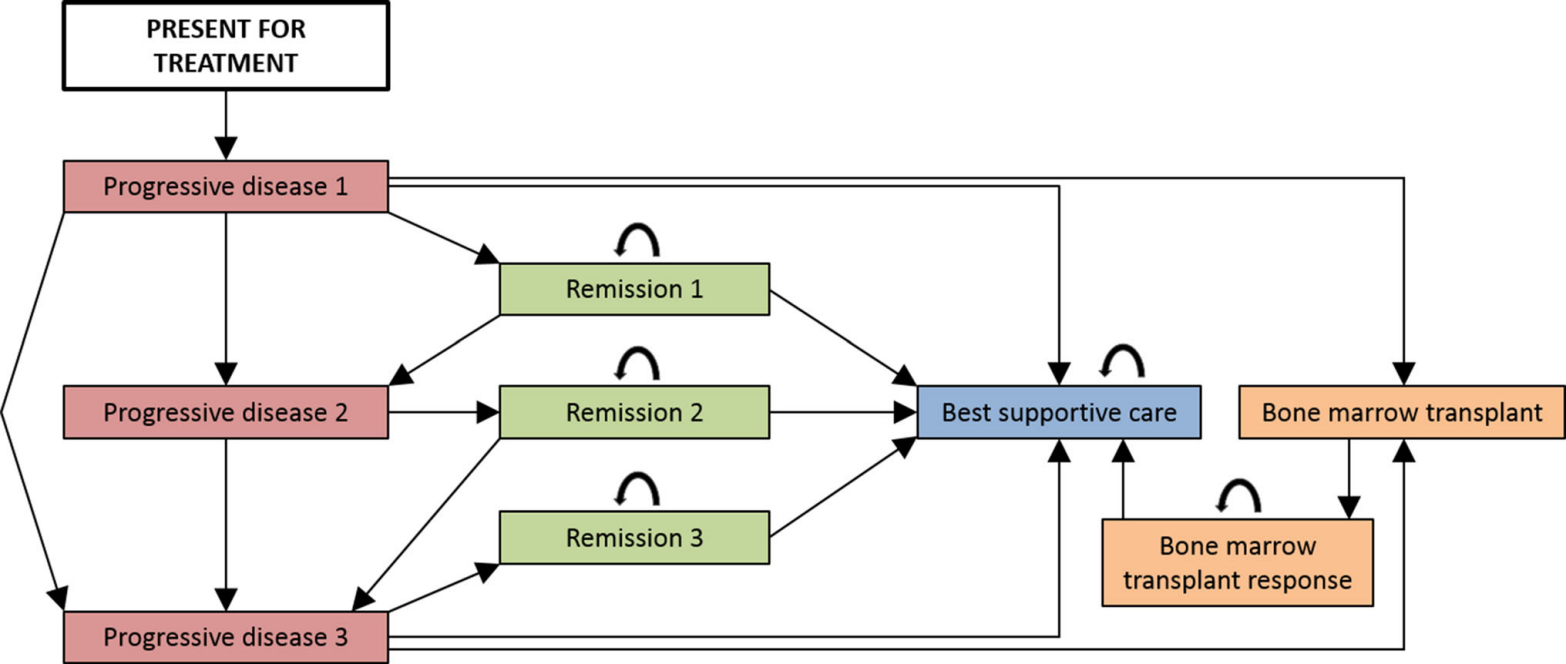
Markov model to simulate the disease pathway of patients who can tolerate aggressive chemotherapy treatment. Patients enter the Markov model when they present for treatment and undergo genetic or genomic testing. All patients who undergo first-line treatment then cycle between remission (progression-free survival) and progressive disease (symptomatic and receiving treatment) until entering best supportive care. Some patients will also undergo allogeneic transplantation (bone marrow transplant). Death from chronic lymphocytic leukaemia-related causes is possible in all non-remission states, and death from other causes is possible in all states; transitions to death states are not indicated in the figure for brevity

The clinical pathways informed the development of a Markov model (Fig. 1). This approach was selected because it enabled the disease pathway of these chronically ill patients to be appropriately simulated. Patients enter the model when they present for treatment and undergo genetic or genomic testing. All patients who undergo first-line treatment then cycle between remission [progression-free survival (PFS)] and progressive disease (symptomatic and receiving treatment) until entering best supportive care. Some patients will also undergo allogeneic transplantation [bone marrow transplant (BMT)]. Death from CLL-related causes is possible in all non-remission states, and death from other causes is possible in all states.

This model was then used to evaluate five strategies in which treatment decisions were guided by genetic or genomic testing (or no testing) (Table 1). These strategies were identified by CLL specialists at OUH as being the most common current strategies and the most likely future strategies. Computational constraints and data limitations precluded the evaluation of all potential comparators. These five strategies comprised three current practice comparators (A, B and C), and two future practice comparators (Interventions 1 and 2). Comparator A reflects current practice in hospitals that use genetic information to stratify patients by likely response to FCR treatment. Comparator B is similar to Comparator A, with patients stratified to ibrutinib instead of FCR treatment. Comparator C reflects current practice in hospitals that do not use genetic information to stratify patients by likely response to FCR treatment. In Intervention 1, patients are stratified using genomic testing into likely FCR responders and non-responders, with non-responders receiving ibrutinib as first-line treatment. Intervention 2 is similar to Intervention 1, but non-responders now receive ibrutinib as refractory treatment. Detailed pathways for all comparators are provided in Appendix 1 of the ESM.

**Table 1:** Strategies evaluated in the economic evaluation

Comparator	Current or future practice?	Pre-treatment genetic or genomic testing?	Ibrutinib used?	Notes
A	Current	Genetic testing	No	This strategy reflects current practice in hospitals that use genetic information to stratify patients by likely response to FCR treatment. Symptomatic patients first undergo genetic testing (FISH testing and Sanger sequencing) to identify those with *TP53* mutations, with the two patient groups then following different clinical pathways. For patients without a *TP53* mutation, first- and second-line treatment is either combination FCR or BR chemotherapy. Patients with refractory disease (or patients who have acquired a *TP53* mutation following an earlier line of treatment) receive ofatumumab combination therapy, with a proportion also undergoing allogeneic transplantation. Patients with a *TP53* mutation receive ofatumumab treatment, consolidated with allogeneic transplantation in a proportion of patients, followed by a second course of ofatumumab treatment if required
B	Current	Genetic testing	Yes	This strategy is similar to Comparator A, but refractory treatment for all patients is now ibrutinib. This is categorised as a current practice comparator as genetic testing is still used to stratify patients to first-line treatment
C	Current	None	No	This strategy reflects current practice in hospitals that do not use genetic information to stratify patients by likely response to FCR treatment. This pathway is the same as that for patients with no *TP53* mutation in Comparator A, with the caveat that in Comparator C, patients cannot move from first-line to refractory treatment as there is no genetic testing (thus, the emergence of a high-risk genetic mutation cannot be identified)
Intervention 1	Future	Genomic testing	Yes	In this strategy, patients are stratified using genomic testing (targeted NGS) into likely FCR responders and non-responders. FCR responders follow a pathway similar to that for patients with *TP53* mutations in Comparator B. Non-responders receive ibrutinib as first-line treatment until they no longer respond, then receive best supportive care
Intervention 2	Future	Genomic testing	Yes	In this strategy, patients are again stratified using genomic testing, with FCR responders following the same pathway as in Intervention 1. However, non-responders now receive first-line ofatumumab treatment and then refractory ibrutinib treatment

*BR* bendamustine and rituximab, *FCR* rituximab, cyclophosphamide and fludarabine, *FISH* fluorescent in situ hybridisation, *NGS* next-generation sequencing

The comparators evaluated using this model were not narrowly defined as current practice (with genetic testing) and future practice (with genomic testing) for three reasons. First, pre-treatment genetic testing is not undertaken in all UK hospitals and there are several ways in which genomic testing could be implemented in CLL. Second, ibrutinib has recently been reviewed by the National Institute for Health and Care Excellence (NICE) for use in patients with relapsed/refractory CLL, and its use has been recommended in patients with specific genetic mutations, provided that the manufacturer provides ibrutinib at an agreed discounted cost [33]. Finally, evidence to support the use of ibrutinib as first-line treatment in likely FCR non-responders is anticipated soon [34].

### Design Considerations

Patients entering the model were assumed to be 65 years old, to reflect the average age of patients with CLL in the UK guidance [18]. Some ‘go-go’ patients are, however, younger, and a scenario analysis considered a younger starting age (see Sect. 2.9). The model cycle length was 28 days, reflecting the usual duration of a cycle of chemotherapy treatment. Patients typically receive a maximum of six 28-day cycles of FCR, bendamustine/rituximab or ofatumumab treatment, and this restriction was applied in the model by implementing tunnel states. Patients had to pass through six treatment tunnel states to reach a remission state, but could move from any of these six states to later lines of treatment (owing to non-response) or to death from any cause. A similar restriction was not applied for ibrutinib treatment as this continues until patients no longer respond. Finally, patients undergoing BMT enter a dedicated tunnel state for one cycle then enter remission. This state contains all of the costs related to the BMT procedure, while the BMT remission state contains all ongoing costs, e.g. related to complications. Detailed descriptions of model health states are provided in Appendix 2 of the ESM.

### Genetic and Genomic Parameters

The genetic parameters were estimated within a study that retrospectively analysed patient DNA samples from four multicentre clinical trials conducted in the UK using targeted NGS (a gene panel test) and a combination of fluorescent in situ hybridisation and Sanger sequencing. The two approaches were compared in terms of the proportion of FCR non-responders that were correctly identified, and these results were combined with data on PFS in multiple logistic regression models. The current practice (fluorescent in situ hybridisation plus Sanger sequencing) model predicted that nine (7%) of the 133 patients in the study would experience disease progression at 36 months. Of these, 78% were true positives. Of the residual group of patients, 69% had not progressed at 36 months. The future practice (targeted NGS) model identified more patients (17%) as non-responders, with a higher true positive rate (82%). Appendix 3 of the ESM describes these parameters in detail.

### Transition Probabilities

Transition probabilities were primarily derived from published studies. Appendix 4 of the ESM describes how these parameters were estimated. Data on ibrutinib treatment efficacy were only available from clinical trials with small sample sizes, short follow-up periods and few patients in clinically relevant genetic subgroups [30, 35–37]. Consequently, the transition probabilities that are informed by these data sources are characterised by uncertainty. Some are based on average treatment effects from different trials, while others are extrapolated from data on treatment efficacy in other patient groups. Data on treatment efficacy for different genetic subgroups were only available for more established treatments (e.g. FCR) [15]. Several approaches were used to estimate these parameters for other treatments, including the application of hazard rates for treatment mortality from one study to treatment response rates from a second study. All of these parameters were varied in sensitivity analyses.

In Interventions 1 and 2, patients undergo genomic testing prior to beginning a new line of treatment. The transition probabilities applied in these scenarios were estimated by interviewing two haematology doctors at OUH. Patients correctly identified as FCR responders were assumed to transition out of first-line FCR treatment according to probabilities that reflected a 20% improvement on standard FCR treatment (in terms of mortality rate, non-response rate and PFS). This adjustment reflects the improved targeting of FCR treatment to likely responders and was varied within a wide range in sensitivity analysis. Patients incorrectly identified as FCR responders do not benefit from this effect, thus they transitioned out of first-line FCR treatment according to the probabilities associated with FCR first-line treatment in patients with *TP53* mutations. A similar improvement in outcomes in likely FCR responders receiving second-line bendamustine/rituximab treatment was assumed. The transition probabilities for patients identified as FCR non-responders were not adjusted, as these patients received ibrutinib, which is likely more efficacious than FCR treatment.

### Resource Use and Unit Cost Data

A UK NHS costing perspective was adopted in the base-case analysis. Resource use in each health state was estimated via interviews with clinicians at OUH. Unit costs were extracted from various databases [32, 38–40]. A microcosting study was conducted alongside the retrospective sample analysis study (described in Sect. 2.4) to estimate the cost of genetic and genomic testing as there are no national price lists for these tests.

Limited data were available on the cost of ibrutinib treatment in the UK. The listed price of a 140-mg tablet is £51.10 (approximately £56,000/year) [32]. A similar cost was identified in a US setting [31]. This cost was considered by clinicians to be unviable in the UK, given budget constraints and cost-effectiveness thresholds. Furthermore, a patient access scheme was in place in the only NICE appraisal of ibrutinib treatment, suggesting that a lower cost is appropriate [38]. The annual cost of treatment was therefore assumed to be £30,000, to match the upper limit of the NICE cost-effectiveness threshold [41]. This assumption was varied in sensitivity analysis.

The costs associated with treating chemotherapy and BMT complications were estimated by combining data on complication rates with unit costs extracted from secondary sources. All costs are reported in British Pounds for the year 2013 to align with the methods and results of the microcosting study of genetic and genomic testing. Table 2 presents the overall costs of each health state. Full details of these calculations are provided in Appendices 5–10 of the ESM.

**Table 2:** Summary of the cost of spending a 28-day cycle in each model health state

State	Comparator	Cost per 28-day cycle (£)
FCR treatment (first-line, first cycle)	A/B	4404
	C	4123
	Intervention 1/2	3958
FCR treatment (second-line, first cycle)	A/B	5373
	C	5092
FCR treatment (subsequent cycles)	A/B/C (first-line)	4289
	A/B/C (second-line)	5258
	Intervention 1/2	3805
BR treatment (first-line, first cycle)	A/B	4535
	C	4254
BR treatment (second-line, first cycle)	A/B	5542
	C	5261
	Intervention 1/2	4573
BR treatment (subsequent cycles)	A/B/C (first-line)	4427
	A/B/C (second-line)	5434
	Intervention 1/2	4427
Ofatumumab treatment (first-line, first cycle)	A/B	12,798
	Intervention 2	12,836
Ofatumumab treatment (first-line, second cycle)	A/B	15,334
Ofatumumab treatment (first-line, subsequent cycles)	A/B	4455
Ofatumumab treatment (second-line or refractory, first cycle)	A/B	13,054
	C	12,773
Ofatumumab treatment (second-line or refractory, second cycle)	A/B/C	15,590
Ofatumumab treatment (second-line or refractory, subsequent cycles)	A/B/C	4711
Ibrutinib treatment (first-line)	Intervention 1	2704
Ibrutinib treatment (second-line or refractory)	B	2780
	Intervention 1/2	2704
Remission following FCR treatment (all lines)	All	212
Remission following BR treatment (all lines)	All	130
Remission following ofatumumab treatment (all lines)	A/B/C/Intervention 2	49
Undergoing BMT	A/B/C/Intervention 2	43,724
Remission following BMT	A/B/C/Intervention 2	233
BSC	All	1650

*BMT* bone marrow transplant, *BR* bendamustine and rituximab, *BSC* best supportive care, *FCR* fludarabine, cyclophosphamide and rituximab

### Health Outcomes

The life-years accrued by patients were estimated in the model and combined with utility weights (extracted from secondary sources [42, 43]; see Appendix 11 of the ESM) to estimate QALYs. These weights reflected general health states (treatment-specific weights were not available), and were adjusted to reflect complication rates. Table 3 presents these weights, with further details provided in Appendix 12 of the ESM.

**Table 3:** Utility weights used to calculate quality-adjusted life-years

Disease state	Utility	Source
Undergoing first-line treatment	0.803	[42]
Undergoing second-line treatment	0.710	[42]
Undergoing refractory treatment	0.650	[42]
Undergoing BMT	0.650	[42]
In remission	0.910	[42]
Disutility associated with grade 3/4 adverse event	−0.133	Calc
Receiving BSC	0.680	[42, 43]

*BMT* bone marrow transplant, *BSC* best supportive care, *Calc* calculated

### Analytical Methods

Cohort analysis was used to conduct the economic evaluation, implemented using Microsoft Excel. Hypothetical cohorts of 10,000 patients were modelled over a 30-year period (effectively a lifetime horizon for 65-year-old patients). A half-cycle correction was applied, with costs and outcomes discounted at a rate of 3.5%, as per NICE guidance [41]. Mean costs and outcomes per patient were calculated for each comparator, along with incremental cost-effectiveness ratios (ICERs). Both dominated and extendedly dominated comparators were excluded in the incremental analysis as CLL genetic testing practice is not consistent across the UK, and this situation is expected to persist when genomic testing is introduced. Incremental cost-effectiveness ratios were compared with a threshold of £30,000 per life-year/QALY gained in the base-case analysis to judge their relative cost effectiveness [41].

### Sensitivity and Scenario Analysis

All parameters were varied in univariate sensitivity analysis. Variations reflected evidence from the literature when available, otherwise parameters were varied by 50% above and below base-case values. Several scenario analyses considered changes in the cost of ibrutinib, variations in the treatment effects applied following genomic testing, different starting ages and different time horizons. Appendix 13 of the ESM describes these analyses. Different thresholds were also applied to judge the relative cost effectiveness of different strategies. These included a more conservative threshold of £20,000 (as per NICE guidelines), and also the NICE end-of-life threshold of £50,000, on the basis that ibrutinib is classed as an orphan drug and a recent appraisal of ibrutinib treatment adopted this higher threshold [33, 41]. Finally, probabilistic sensitivity analysis (PSA) was undertaken, with distributions assigned to parameters based on measures of variance reported in the original studies [44]. Transition probabilities and utility weights were assumed to follow a beta distribution, with complication rates and other parameters related to resources assumed to follow a gamma distribution. Where measures of variance were not available to parameterise distributions in the PSA, the coefficient of variation was assumed to be 0.10 [45]. The results of 1000 PSA simulations were used to calculate cost-effectiveness acceptability curves.

### Applying a Societal Analytical Perspective

It has been noted that economic evaluations of genomic tests may require the use of a societal costing perspective as such interventions may have impacts beyond the healthcare sector [10]. Furthermore, the latest guidelines from the Second Panel on Cost-Effectiveness in Health and Medicine also recommend extending analyses to consider this perspective [46], and a recent systematic review of the economic burden and quality-of-life effects of CLL concluded that there is a need for studies in CLL that take a societal perspective [47]. The base-case analysis was therefore extended to consider a societal perspective. Three categories of costs were included: productivity costs (with the costs of long-term absenteeism estimated by applying the friction cost approach); informal care costs and out-of-pocket costs, informed by guidelines [48–52]. Data for this analysis were extracted from several sources, including journal articles and reports from charities [53, 54], and all parameters were varied within the sensitivity analysis and PSA. Appendix 14 of the ESM describes this analysis in detail.

### Research Reporting Guidelines

This economic evaluation follows the reporting guidelines specified in the Consolidated Health Economic Evaluation Reporting Standards statement [55]. Appendix 15 of the ESM provides a completed checklist.

## Results

The base-case results are presented in Table 4. Comparator C (current practice with no genetic testing) was the cheapest strategy while Intervention 1 (genomic testing followed by first-line ibrutinib treatment in likely FCR non-responders) was the most expensive. Comparator B (current practice in hospitals that use genetic information to stratify patients by likely response to FCR, with ibrutinib given as refractory treatment for all patients) yielded the most life-years per patient while Intervention 1 yielded the most QALYs per patient. This difference reflects the fact that although refractory ibrutinib treatment in current practice (Comparator B) generates gains in life expectancy, once quality of life in these additional life-years is considered, first-line ibrutinib treatment following genomic testing (Intervention 1) offers greater health gains. In both analyses, Comparator C yielded the fewest health gains, while Comparator A (current practice in hospitals that use genetic information to stratify patients by likely response to FCR) was the most cost-effective strategy at a threshold of £30,000 per life-year/QALY gained.

**Table 4:** Base-case results for the cost-effectiveness analysis (CEA) and cost-utility analysis (CUA)

Analysis	Comparator	Mean LYs/QALYs per patient	Mean costs per patient	ICER [excluding dominated (DOM) strategies]	ICER [excluding extendedly dominated strategies (EXT.DOM)]
CEA	C	6.37	£69,704		
	**A**	**6.61**	**£71,576**	**£7903**	**£7903**
	Int 2	6.65	£91,790	£580,390	EXT.DOM
	B	7.63	£107,703	£16,133	£35,376
	Int 1	7.45	£119,088	DOM	DOM
CUA	C	5.60	£69,704		
	**A**	**5.82**	**£71,576**	**£8565**	**£8565**
	Int 2	5.93	£91,790	£177,198	EXT.DOM
	B	6.44	£107,703	£31,153	EXT.DOM
	Int 1	6.67	£119,088	£50,559	£55,891

The most cost-effective strategy at a threshold of £30,000 per LY/QALY gained is highlighted in bold

*ICER* incremental cost-effectiveness ratio, *Int 1* Intervention 1, *Int 2* Intervention 2, *LYs* life-years, *QALYs* quality-adjusted life-years

### Sensitivity Analysis

The results were robust to changes in single parameters (results presented in Appendix 16 of the ESM). There were only 11 parameter variations that changed the most cost-effective strategy from Comparator A to an alternative strategy, in either the CEA or the CUA. These included variations in the prevalence of *TP53* mutations, the CLL mortality rate during first-line FCR treatment, and the discount rate applied to costs and health outcomes. One variation changed the most cost-effective strategy to an intervention strategy. This was an improvement in PFS following first-line FCR treatment for FCR responders who had been correctly identified via genomic testing. This changed the most cost-effective strategy to Intervention 2 (genomic testing followed by refractory ibrutinib treatment in likely FCR non-responders) in both the CEA (£21,053/life-year gained) and the CUA (£19,248/QALY gained).

The model was also sensitive to changes in the mortality rate for patients receiving first-line FCR treatment. When this was increased in patients without *TP53* mutations, the most cost-effective strategy changed to Comparator C in the CEA (£5855/life-year gained) and CUA (£6921/QALY gained). This suggests that if the mortality rate for patients selected by genetic testing to receive FCR treatment approaches that for patients with *TP53* mutations receiving first-line treatment, the benefit associated with genetic stratification disappears. Similarly, when the FCR mortality rate was decreased in patients of unknown *TP53* mutation status, the most cost-effective strategy again changed to Comparator C in the CEA (£7245/life-year gained) and CUA (£8402/QALY gained). Again, as the outcomes gap between the patient groups disappears, so does the benefit associated with genetic stratification.

### Scenario Analysis

Appendix 17 of the ESM reports the results of the scenario analyses. When the cost per cycle of ibrutinib treatment was reduced by 75% to £575 per cycle, the most cost-effective strategy in both the CEA and CUA was Intervention 1 (Scenario 1). A threshold analysis indicated that Intervention 1 becomes the most cost-effective strategy in the CEA when the cost falls to £602 per cycle (an annual treatment cost of £7848), and in the CUA when the cost falls to £1511 per cycle (an annual treatment cost of £19,830).

Variations in the improved first- and second-line treatment effects applied following genomic testing for patients identified as FCR responders also impacted on the results (Scenario 3). Moving from 20 to 40% changed the most cost-effective strategy to Intervention 2 in both the CEA and CUA, mainly owing to an improvement in health outcomes (rather than a reduction in health costs). Variations in patient starting age (Scenario 4) had no effect when younger patients (aged 25 years) were considered. However, when patients aged 85 years were considered, the most cost-effective strategy changed to Comparator C. When the underlying mortality rate increases, it becomes less important to stratify patients by response to treatment. However, few ‘go-go’ patients are aged 85 years or over.

Scenario 7 isolated the contribution of ibrutinib treatment to these results by equalising the cost and performance of genetic and genomic testing. Comparator A remained the most cost-effective strategy in both the CEA and CUA, indicating that the cost effectiveness of using genomic information to guide treatment decisions is driven by the relative costs and consequences of the different treatment options, not the costs and consequences of genomic testing.

Finally, the most cost-effective strategy (Comparator A) remains the same when a conservative threshold of £20,000 per life-year/QALY gained is applied. However, if the NICE end-of-life threshold of £50,000 is applied, Comparator B becomes the most cost-effective strategy in the CEA, while Intervention 1 approaches cost effectiveness in the CUA.

### Societal Perspective Results

Table 5 presents the results of this analytical extension. The increase in cost per patient for each comparator is minimal compared with the base-case results, hence the ICERs are approximately the same. The only variation that changes this result is the use of different starting ages. Table 6 presents the results from a societal perspective for patients aged 25 years. Costs are higher for all strategies because patients with CLL now die at a younger age, increasing the costs associated with premature mortality. Consequently, the most cost-effective strategy in the CEA changes to Comparator B (£2943/life-year gained). The most cost-effective strategy in the CUA also changes, but to Intervention 1 (£19,933/QALY gained). At younger ages, a strategy that includes ibrutinib treatment is likely to be the most cost-effective option from a societal perspective, as this minimises the costs associated with premature mortality.

**Table 5:** Results for the cost-effectiveness analysis (CEA) and the cost-utility analysis (CUA) from a societal perspective

Analysis	Comparator	Mean LYs/QALYs per patient	Mean costs per patient	ICER [excluding dominated (DOM) strategies]	ICER [excluding extendedly dominated (EXT-DOM) strategies]
CEA	C	6.37	£73,832		
	**A**	**6.61**	**£76,035**	**£9302**	**£9302**
	Int 2	6.65	£95,031	£545,428	EXT.DOM
	B	7.63	£110,820	£16,007	£34,062
	Int 1	7.45	£122,116	DOM	DOM
CUA	C	5.60	£73,832		
	**A**	**5.82**	**£76,035**	**£10,081**	**£10,081**
	Int 2	5.93	£95,031	£166,523	EXT.DOM
	B	6.44	£110,820	£30,908	EXT.DOM
	Int 1	6.67	£122,116	£50,164	£54,207

The most cost-effective strategy at a threshold of £30,000 per LY/QALY gained is highlighted in bold

*ICER* incremental cost-effectiveness ratio, *Int 1* Intervention 1, *Int 2* Intervention 2, *LYs* life-years, *QALYs* quality-adjusted life-years

**Table 6:** Results for the cost-effectiveness analysis (CEA) and the cost-utility analysis (CUA) from a societal perspective for patients aged 25 years

Analysis	Comparator	Mean LYs/QALYs per patient	Mean costs per patient (£)	ICER [excluding dominated (DOM) strategies]	ICER [excluding extendedly dominated (EXT.DOM) strategies]
CEA	Int 2	7.73	687,062		
	**B**	**8.88**	**690,438**	**£2943**	**£2943**
	A	7.35	697,523	DOM	DOM
	Int 1	8.73	698,459	DOM	DOM
	C	7.00	707,752	DOM	DOM
CUA	Int 2	6.91	687,062		
	B	7.42	690,438	£6701	£6701
	A	6.47	697,523	DOM	DOM
	**Int 1**	**7.82**	**698,459**	**£19,933**	**£19,933**
	C	6.15	707,752	DOM	DOM

The most cost-effective strategy at a threshold of £30,000 per LY/QALY gained is highlighted in bold

*ICER* incremental cost-effectiveness ratio, *Int 1* Intervention 1, *Int 2* Intervention 2, *LYs* life-years, *QALYs* quality-adjusted life-years

### Cost-Effectiveness Acceptability Curves

**Fig. 2:**
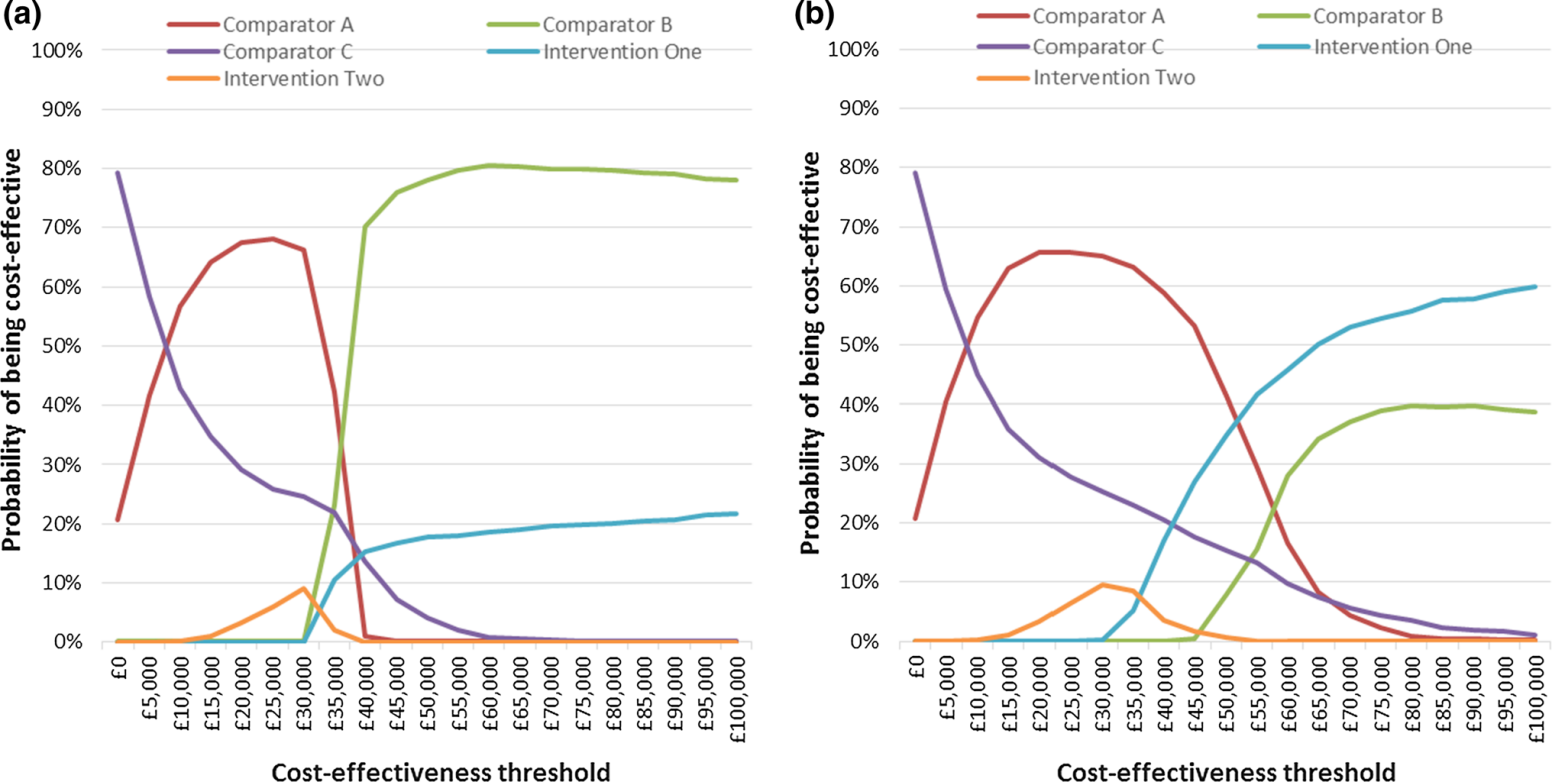
Cost-effectiveness acceptability curves for **a** the cost-effectiveness analysis, and **b** the cost-utility analysis. The cost-effectiveness acceptability curves indicate the probability that each comparator strategy is cost effective at a range of threshold values that a decision maker might be willing to pay for an additional life-year or quality-adjusted life-year. In both the cost-effectiveness analysis and cost-utility analysis, the most cost-effective strategy at a threshold of £30,000 per life-year/quality-adjusted life-year gained is Comparator A. The lowest threshold value at which a genomic testing strategy becomes the most cost-effective option is between £50,000 and £55,000 in the cost-utility analysis

Figure 2 presents the cost-effectiveness acceptability curves for the CEA and CUA. These confirm that the most cost-effective strategy at a threshold of £30,000 is Comparator A, although there was only a 65–66% probability of this conclusion being correct. In the CEA, there is no threshold value up to £100,000 at which either of the genomic testing interventions are the most cost-effective option. In the CUA, Intervention 1 becomes the most cost-effective strategy at a threshold between £50,000 and £55,000.

## Discussion

This article presents an economic evaluation of the use of genomic testing to guide CLL treatment decisions in the UK NHS. The results suggest that current genetic testing practice without ibrutinib treatment is the most cost-effective strategy at a threshold of £30,000 per life-year/QALY gained. However, if a higher ‘end-of-life’ cost-effectiveness threshold of £50,000 is applied, strategies that use genomic information to stratify patients to ibrutinib treatment become cost effective.

The key factor driving the cost effectiveness of the two genomic testing strategies is not the cost or performance of genomic testing compared with genetic testing; it is the cost of ibrutinib treatment. Indeed, a reduction in this cost leads to Intervention 1 (genomic testing followed by first-line ibrutinib treatment in likely FCR non-responders) becoming the most cost-effective strategy. This is of particular note because the cost of ibrutinib treatment in the UK remains unclear, and the costs applied in this analysis were assumptions based on the anticipated behaviour of the manufacturer. This finding suggests that if shorter periods of ibrutinib therapy were shown in clinical trials to have a similar positive effect on health outcomes (e.g. in combination with rituximab), or if the cost of ibrutinib were to be reduced by the manufacturer to reflect its potential use as first-line therapy in all likely FCR non-responders, genomic testing strategies may become cost effective at the standard NICE threshold of £20,000–30,000 per QALY gained as well as the higher end-of-life threshold.

An important methodological consideration when conducting an economic evaluation of a genomic technology is selecting an appropriate analytical approach. In this study, the most cost-effective strategy did not vary when the analytical perspective in the base-case analysis was widened to incorporate societal costs. However, the base-case analysis focused on 65-year-old patients. Although many ‘go-go’ patients are of older age, there is also a subgroup of younger patients who can tolerate aggressive chemotherapy treatments, and there was evidence that genomic testing could be cost effective in this population when a societal perspective is adopted. Future studies should consider whether the use of genomic testing to guide treatment decisions could improve productivity and avoid mortality in younger patients, as these cost savings could be substantial.

Furthermore, some societal costs that are likely important to older patients were not included in this analysis, including the costs associated with admission to a nursing home. Ibrutinib treatment may prevent such admissions as patients with effectively treated disease are better able to cope with other co-morbidities. If these costs had been included, it is possible that strategies that include ibrutinib treatment would also be cost effective in older patients when a societal perspective is considered. Future studies evaluating genomic tests to guide treatment decisions in older patients should consider quantifying these costs.

No other published economic evaluations of the use of genomic testing to guide CLL treatment decisions were identified in the literature. However, a recent NICE appraisal considered the cost effectiveness of ibrutinib treatment in patients with previously treated CLL. This appraisal concluded that “the ICERs for ibrutinib fell within the range normally considered as a cost-effective use of NHS resources for a treatment that fulfils the end-of-life criteria” [33].

The finding that Comparator A is the most cost-effective strategy has a number of implications both for current clinical practice in the UK NHS, and also more generally for economic evaluations of genomic interventions. This finding implies that hospitals in which clinical practice is closely aligned with Comparators B or C should instead modify practice to match Comparator A. Such a change in clinical practice may not be feasible for these hospitals, particularly if there is limited access to genetic testing. The same issue may arise in future studies if cost-effective genomic testing strategies are identified. Given this, future economic evaluations of genomic interventions in this and other clinical contexts should consider collecting information on the barriers and facilitators of implementation to provide important context for the economic evaluation results.

This article has presented one of the most comprehensive economic evaluations conducted to date in both a CLL context, and also in precision medicine more generally. Several current and likely future clinical pathways have been evaluated from multiple analytical perspectives, informed by the latest clinical trial data and reflecting a range of different treatments and treatment stratification strategies. However, a number of limitations should be noted. First, there is a lack of evidence on the effectiveness of CLL chemotherapy treatments; there are relatively few good-quality, phase III clinical trials with large sample sizes. In those that do exist, there are limited data on treatment efficacy in certain patient subgroups (e.g. patients with genetic abnormalities). Consequently, several of the transition probabilities in this model were calculated by combining information from disparate data sources, and a meta-analysis was not undertaken. The results of the sensitivity analysis presented in this article suggest that variations in these parameters (such as the mortality rate for patients with and without *TP53* mutations receiving first-line FCR treatment) could change the most cost-effective strategy to a strategy in which genetic information is not used to inform treatment decisions. Generating more precise estimates of such parameters in good-quality clinical studies should be a priority going forward.

A second limitation is that this economic evaluation assumed a constant benefit from ibrutinib treatment over the model course. If the duration of benefit from ibrutinib was instead decreased over time, this would reduce the health and quality-of-life outcomes associated with strategies in which ibrutinib was used, further reducing the likelihood that such strategies are cost effective. Third, some of the transition probabilities related to FCR treatment outcomes were estimated by interviewing clinical experts. This approach to expert elicitation differs from that recommended in the most recent published guidelines [56]. Future studies estimating such parameters by consulting clinical experts should follow these guidelines. Fourth, the utility weights that were applied were specific to lines of chemotherapy treatment, hence differences in utility by type of treatment were not captured. However, these weights were adjusted using information on complication rates and disutility weights, which may modify this limitation. Furthermore, there were no variations in utility weights that changed the most cost-effective strategy from Comparator A to an alternative strategy in the sensitivity analysis. Fifth, the annual cost of ibrutinib treatment is assumed to be £30,000, which is lower than the cost in USA. This assumption is supported by the fact that a confidential patient access scheme is in place in the UK. Even with this low cost in place, strategies that include ibrutinib treatment are only cost effective when a higher ‘end-of-life’ cost-effectiveness threshold is applied, which suggests that a higher cost would not have changed the main conclusions of this study. Sixth, this study conducted a CEA and CUA to evaluate the costs and health outcomes associated with different test treatment strategies, as per NICE guidance [41]. An alternative analytical approach could have yielded a different adoption decision, and there is a growing literature recommending the application of multiple-criteria decision analysis for the evaluation of orphan drugs such as ibrutinib [57–59]. Finally, the economic evaluation considered five test-treatment strategies. Considering just a subset of all possible strategies in an economic evaluation adds an additional layer of uncertainty to the study results.

## Conclusion

This study represents one of the most comprehensive economic evaluations conducted to date in CLL precision medicine, and contributes to the literature on economic evaluation and genomic testing more generally by considering multiple analytical perspectives. The results suggest that stratifying patients with CLL to targeted treatment using genomic testing is not a cost-effective use of limited NHS resources, primarily owing to the high cost of ibrutinib treatment. However, if a higher end-of-life cost-effectiveness threshold is applied, or if a societal costing perspective is considered in younger patients, genomic testing strategies become cost effective. Although this study focused on a particular application of genomic testing, the characteristics of this case study may be shared by other genomic interventions that facilitate a precision medicine approach, particularly those interventions that inform treatment decisions and which cost relatively little compared with the cost of treatment. These results may therefore be broadly indicative of the likely cost effectiveness of genomic tests that inform the stratification of patients to high-cost clinically effective therapies, particularly in other chronic disease or cancer settings such as colorectal cancer [60].

## Electronic supplementary material

Below is the link to the electronic supplementary material.

Supplementary material 1 (DOCX 578 kb)
