# Supplementary material for: Using Genomic Information to Guide Ibrutinib Treatment Decisions in Chronic Lymphocytic Leukaemia: A Cost-Effectiveness Analysis
Source: Pharmacoeconomics. 2017 Jul 31;35(8):845–58. doi: 10.1007/s40273-017-0519-z (PMC5548825; doi:10.1007/s40273-017-0519-z)
Supplement: Supplementary file 1 — Supplementary material 1 (DOCX 578 kb) [file 40273_2017_519_MOESM1_ESM.docx]

**Supplementary File**

**Using genomic information to guide ibrutinib treatment decisions in chronic lymphocytic leukaemia: a cost-effectiveness analysis**

James Buchanan, Sarah Wordsworth, Ruth Clifford, Pauline Robbe, Jenny C Taylor, Anna Schuh, Samantha JL Knight

*PharmacoEconomics*

Contents

[Appendix One: Detailed clinical pathways for all comparators evaluated using the Markov model 2](#_Toc482029073)

[Appendix Two: Descriptions of all model health states 6](#_Toc482029074)

[Appendix Three: Genetic and genomic parameters 8](#_Toc482029075)

[Appendix Four: Transition probabilities 10](#_Toc482029076)

[Appendix Five: Unit costs 18](#_Toc482029077)

[Appendix Six: Complication rates 21](#_Toc482029078)

[Appendix Seven: Costs associated with treating chemotherapy complications 23](#_Toc482029079)

[Appendix Eight: Costs associated with treating bone marrow transplant complications 24](#_Toc482029080)

[Appendix Nine: Average length of chemotherapy treatment 25](#_Toc482029081)

[Appendix Ten: Resource use and overall costs for each health state 26](#_Toc482029082)

[Appendix Eleven: Literature review to identify CLL utility values 37](#_Toc482029083)

[Appendix Twelve: CLL utility values 38](#_Toc482029084)

[Appendix Thirteen: Parameter variations considered in the sensitivity and scenario analyses 39](#_Toc482029085)

[Appendix Fourteen: Extending the economic evaluation to consider a societal perspective 46](#_Toc482029086)

[Appendix Fifteen: CHEERS checklist 50](#_Toc482029087)

[Appendix Sixteen: Parameter variations which change the base case analysis results 53](#_Toc482029088)

[Appendix Seventeen: Scenario analysis results 55](#_Toc482029089)

[Appendix Eighteen: Results from a societal perspective 61](#_Toc482029090)

[References 65](#_Toc482029091)

# Appendix One: Detailed clinical pathways for all comparators evaluated using the Markov model

Note: labels in red refer to transition probabilities (R = residual probability).

Comparator A pathways


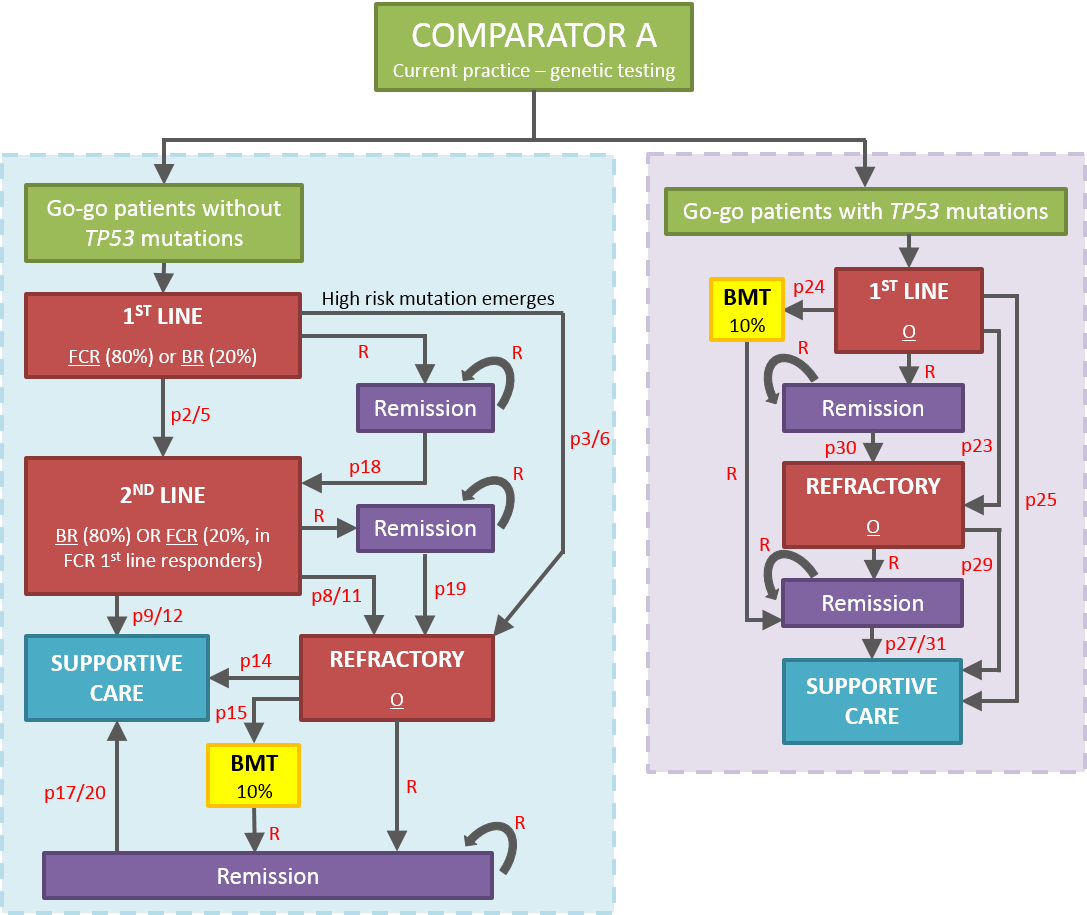


BMT = bone marrow transplant; BR = bendamustine and rituximab; FCR = rituximab, cyclophosphamide and fludarabine; O = ofatumumab.

Comparator B pathways


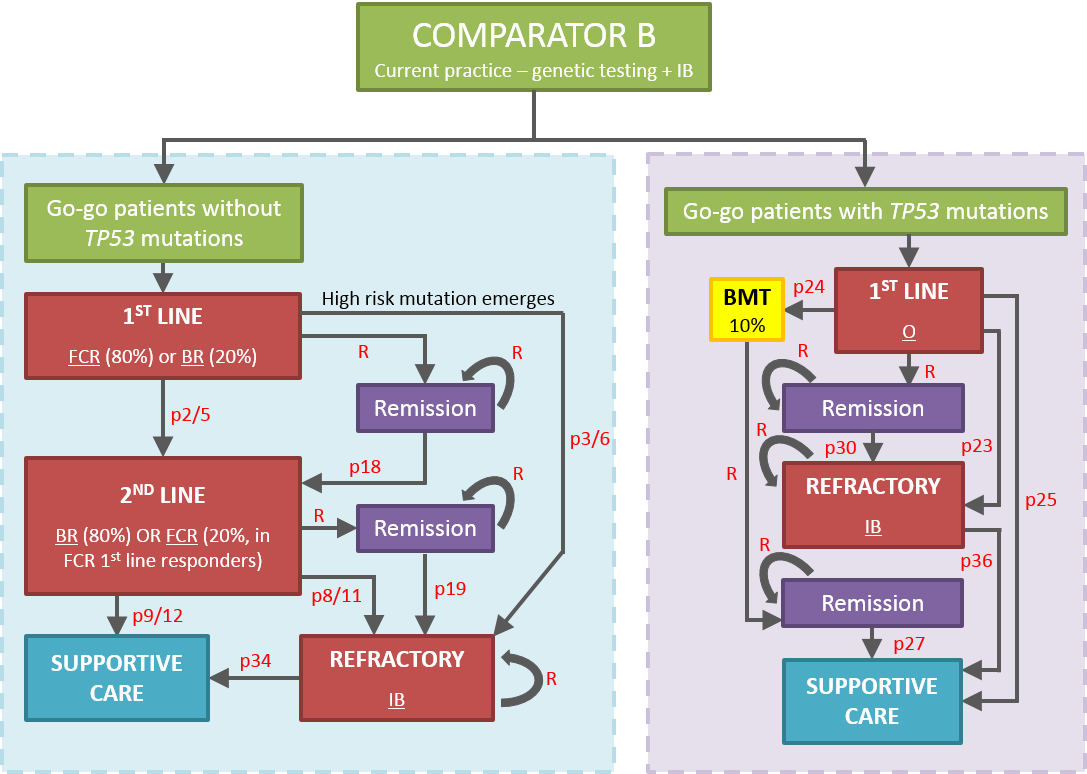


BMT = bone marrow transplant; BR = bendamustine and rituximab; FCR = rituximab, cyclophosphamide and fludarabine; IB = ibrutinib; O = ofatumumab.

Comparator C pathways


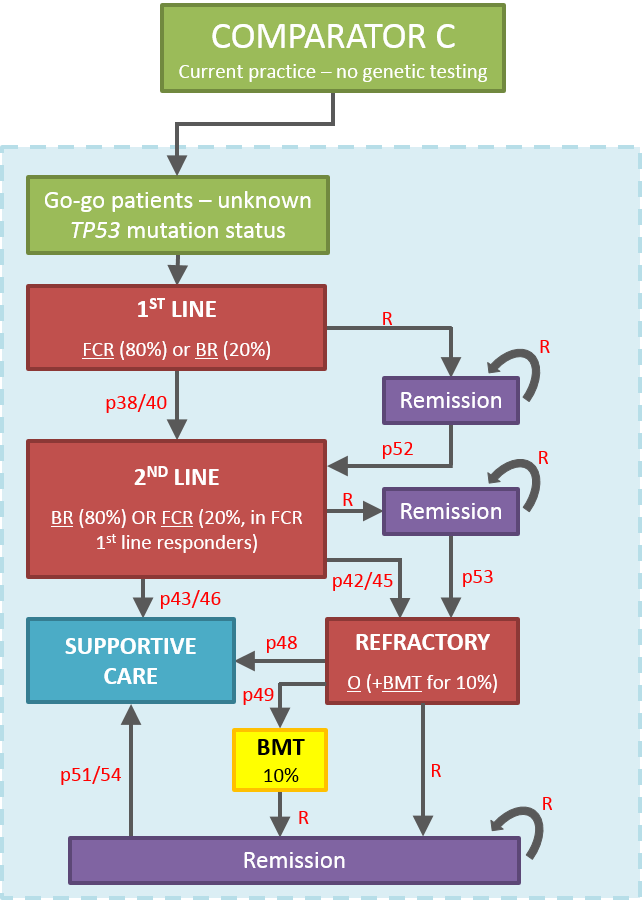


BMT = bone marrow transplant; BR = bendamustine and rituximab; FCR = rituximab, cyclophosphamide and fludarabine; O = ofatumumab.

**Intervention One pathways**


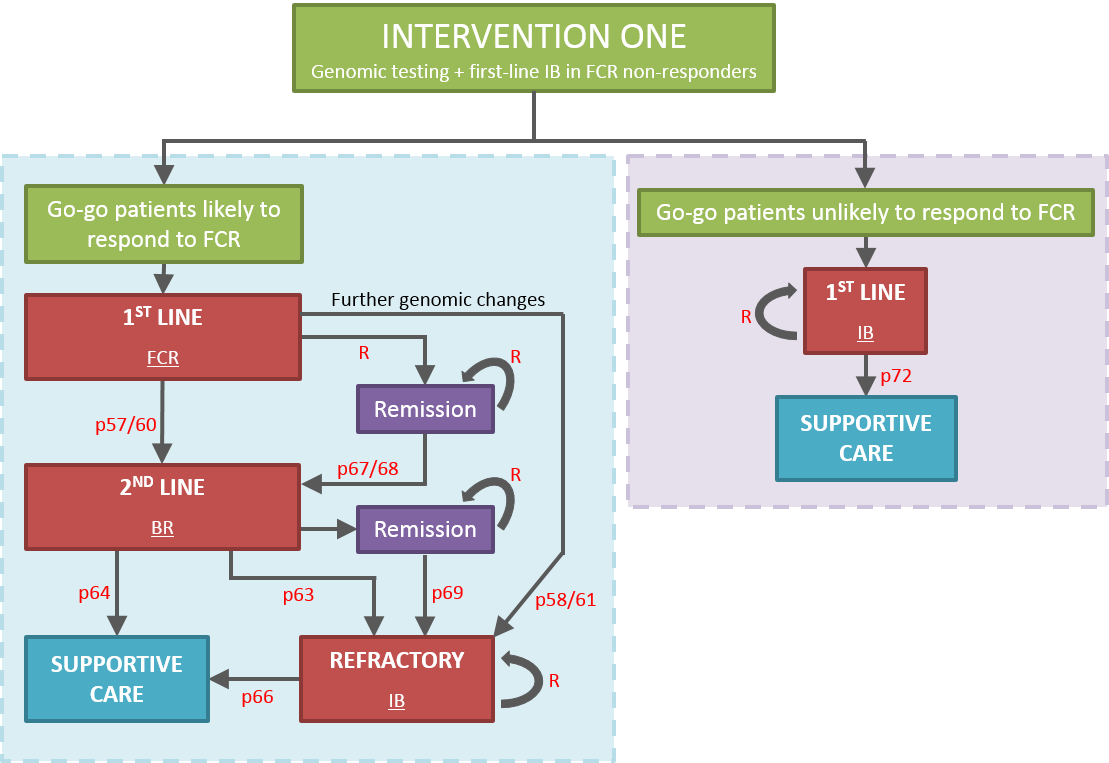


BR = bendamustine and rituximab; FCR = rituximab, cyclophosphamide and fludarabine; IB = ibrutinib.

Intervention Two pathways


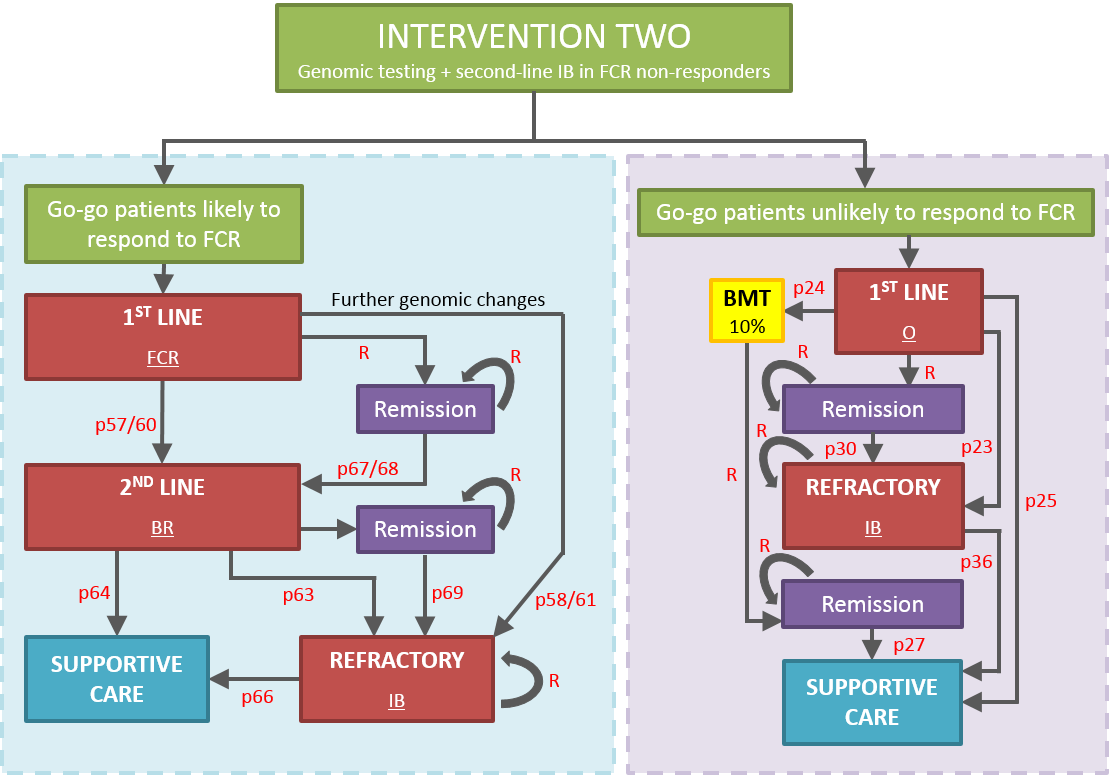


BMT = bone marrow transplant; BR = bendamustine and rituximab; FCR = rituximab, cyclophosphamide and fludarabine; IB = ibrutinib; O = ofatumumab.

# Appendix Two: Descriptions of all model health states

| **State** | **Description** |
| --- | --- |
| FCR treatment (first-line, first cycle) | Patients undergo a 28-day cycle of FCR treatment, guided by genetic or genomic testing in some cases. All receive concomitant medications and periodically undergo diagnostic tests and attend outpatient appointments. Some patients require blood transfusions. |
| FCR treatment (second-line, first cycle) | Patients undergo a 28-day cycle of FCR treatment, guided by genetic testing in some cases. All receive concomitant medications and periodically undergo diagnostic tests and attend outpatient appointments. Some patients require blood transfusions. |
| FCR treatment (first- or second-line, subsequent cycles) | Patients undergo a further 28-day cycle of FCR treatment. All receive concomitant medications and periodically undergo diagnostic tests and attend outpatient appointments. Some patients require blood transfusions. |
| BR treatment (first-line, first cycle) | Patients undergo a 28-day cycle of BR treatment, guided by genetic testing in some cases. All receive concomitant medications and periodically undergo diagnostic tests and attend outpatient appointments. Some patients require blood transfusions. |
| BR treatment (second-line, first cycle) | Patients undergo a 28-day cycle of BR treatment, guided by genetic or genomic testing in some cases. All receive concomitant medications and periodically undergo diagnostic tests and attend outpatient appointments. Some patients require blood transfusions. |
| BR treatment (first- or second-line, subsequent cycles) | Patients undergo a further 28-day cycle of BR treatment. All receive concomitant medications and periodically undergo diagnostic tests and attend outpatient appointments. Some patients require blood transfusions. |
| Ofa treatment (first-line, first cycle) | Patients undergo a 28-day cycle of Ofa treatment, guided by genetic or genomic testing. All receive concomitant medications and periodically undergo diagnostic tests and attend outpatient appointments. Some patients require blood transfusions. |
| Ofa treatment (first-line, second cycle) | Patients undergo a further 28-day cycle of Ofa treatment. All receive concomitant medications and periodically undergo diagnostic tests and attend outpatient appointments. Some patients require blood transfusions. |
| Ofa treatment (first-line, subsequent cycles) | Patients undergo a further 28-day cycle of Ofa treatment. All receive concomitant medications and periodically undergo diagnostic tests and attend outpatient appointments. Some patients require blood transfusions. |
| Ofa treatment (second-line or refractory, first cycle) | Patients undergo a 28-day cycle of Ofa treatment, guided by genetic testing in some cases. All receive concomitant medications and periodically undergo diagnostic tests and attend outpatient appointments. Some patients require blood transfusions. |
| Ofa treatment (second-line or refractory, second cycle) | Patients undergo a further 28-day cycle of Ofa treatment. All receive concomitant medications and periodically undergo diagnostic tests and attend outpatient appointments. Some patients require blood transfusions. |
| Ofa treatment (second-line or refractory, subsequent cycles) | Patients undergo a further 28-day cycle of Ofa treatment. All receive concomitant medications and periodically undergo diagnostic tests and attend outpatient appointments. Some patients require blood transfusions. |
| Ibrutinib treatment (first-line) | Patients undergo a 28-day cycle of ibrutinib treatment, guided by genomic testing in some cases. All periodically undergo diagnostic tests and attend outpatient appointments. Some patients require blood transfusions. |
| Ibrutinib treatment (second-line or refractory) | Patients undergo a 28-day cycle of ibrutinib treatment, guided by genetic or genomic testing. All periodically undergo diagnostic tests and attend outpatient appointments. Some patients require blood transfusions. |
| Remission following FCR treatment (all lines) | Patients are in remission for a 28-day cycle. Some take concomitant medications or undergo IV immunoglobulin treatment. All have periodic outpatient appointments and FBCs. |
| Remission following BR treatment (all lines) | Patients are in remission for a 28-day cycle. Some undergo IV immunoglobulin treatment. All have periodic outpatient appointments and FBCs. |
| Remission following Ofa treatment (all lines) | Patients are in remission for a 28-day cycle. All have periodic outpatient appointments and FBCs. |
| Undergoing BMT | Patient has a BMT. This is assumed to take up one 28-day cycle. |
| Remission following BMT | Patients are in remission following BMT for a 28-day cycle. All have periodic consultant and nurse outpatient appointments, and some telephone consultations. Various tests are performed periodically, and some patients require IV immunoglobulin treatment, donor lymphocyte infusions or blood and platelet support. |
| BSC | Patients spend a 28-day cycle undergoing treatment which is aimed at managing symptoms. All will have an outpatient appointment and FBC. Some will require blood transfusions or an inpatient stay. |

BMT = bone marrow transplant; BR = bendamustine and rituximab; BSC = best supportive care; FBC = full blood count; FCR = fludarabine, cyclophosphamide and rituximab; IV = intravenous; Ofa = ofatumumab.

# Appendix Three: Genetic and genomic parameters

Data on the efficacy of current genetic testing practice and future genomic testing practice was provided by a retrospective study [1]. This was the only study identified in the literature that had investigated this research question. In this study, patient DNA samples from four multicentre clinical trials conducted in the UK underwent FISH testing and Sanger sequencing (current genetic testing practice) and targeted NGS (future genomic testing practice). The aim of both approaches was to identify genetic abnormalities such as *TP53* mutations, which have proven associations with prognosis and response to treatment in CLL. This study also provided evidence of new associations between abnormalities such as *SAMHD1* mutations and treatment outcomes [2]. The two testing approaches were compared in terms of the quantity and type of abnormalities detected, and these results were combined with data on progression free survival (PFS – a common surrogate for remission in CLL) at 36 months in a series of multiple logistic regression models. These models predicted which patients would be PFS positive at 36 months (i.e. which patients had experienced disease progression). Model performance was assessed by comparing model predictions with actual data on PFS at 36 months.

This study has been described by Clifford *et al.* [3] and the results that are relevant to this economic evaluation are summarised in the table below. The key results are contained in three regression models which reflect current genetic testing practice and two possible configurations of future genomic testing practice. The current practice model used information on *TP53* mutation status from FISH testing and Sanger sequencing to predict that 7% of patients would be PFS positive at 36 months. Of these, 78% were true positives. Of the residual group of 124 patients, 85 (69%) had not progressed at 36 months. Information on the proportion of patients in the residual and PFS positive groups was used in Comparators A and B in the Markov model in order to divide patients into those who did and did not have *TP53* mutations.

Comparison between current genetic testing practice and future genomic testing practice

|  | **Number of patients** | | |
| --- | --- | --- | --- |
|  | **Current genetic testing practice** | **Future genomic testing practice 1** | **Future genomic testing practice 2** |
|  | ***TP53* only** | ***TP53* >5%** | ***TP53* >5% + *SAMHD1*** |
| Predicted PFS positive | 7% (^9^/_133_) | 11% (^15^/_132_) | 17% (^22^/_132_) |
| True PFS positive (as % of predicted PFS positive) | 78% (^7^/_9_) | 87% (^13^/_15_) | 82% (^18^/_22_) |
| Residual group | 93% (^124^/_133_) | 89% (^117^/_132_) | 83% (^110^/_132_) |
| Patients not progressing as % of residual group | 69% (^85^/_124_) | 72% (^84^/_117_) | 75% (^82^/_110_) |
| Total patients | 133 | 132 | 132 |

PFS = progression-free survival.

The two models of future genomic testing practice both used information from targeted NGS on *TP53* mutation status to divide patients into likely FCR responders and non-responders. Specifically, these models considered patients with a *TP53* variant allele frequency (VAF) greater than 5%. VAF is a measure of the proportion of a specific gene variant (allele) among all allele copies being considered. This restriction meant that the future practice models focused on those samples in which *TP53* mutations were relatively more frequently observed (this is possible because targeted NGS provides additional information compared to FISH testing and Sanger sequencing). In addition, the second model of future genomic testing practice used information on the presence of *SAMHD1* mutations, another abnormality correlated with treatment outcomes.

In the first model of future practice, 11% of patients were predicted to be PFS positive at 36 months. The residual group – patients who would be considered to be FCR responders – contained 117 patients (89% of all patients). Of these patients, data on PFS at 36 months showed that 84/117 (72%) were actually in PFS at this time. The addition of information on *SAMHD1* mutation status in the second model of future practice resulted in fewer patients being classed as FCR responders (83%), but more of these patients (75%) were correctly identified. For this reason, this second model was used in the base case analysis to divide patients in Interventions One and Two into FCR responders and non-responders, and to divide FCR responders into those who were correctly and incorrectly identified as such. However, the use of the first model was evaluated in a sensitivity analysis.

# Appendix Four: Transition probabilities

The transition probabilities in the model were primarily derived from published studies reporting the results of clinical trials of CLL treatments. Where possible, these probabilities were estimated using data extracted from Phase III studies. However, this model considered a variety of treatments, in first-line, second-line and as refractory therapies, and for different genetic subgroups of patients. Phase III trial data were therefore not available to inform every transition probability required in the model. In these cases, Phase II and Phase II/Ib trial data were used.

In addition to the three general comments concerning transition probabilities in the main paper, two other points should be noted. First, this paper considers the stratification of patients with *TP53* mutations to different treatments. Patients with 17p deletions are also likely to benefit from stratification to treatment in the same way. However, a UK National Institute for Health and Care Excellence (NICE) committee noted in a recent appraisal that both 17p deletions and *TP53* mutations are “*on the same gene locus and tended to appear together in the same people*”. The committee also noted that “*the response would be similar in both populations*” and that it was reasonable to extrapolate data from one genetic abnormality to the other in this instance [4].

Second, as per a recent NICE appraisal, and in the absence of any evidence to the contrary, this analysis assumes a constant benefit from ibrutinib over the model course [4].

The table on the following page reports all transition probabilities used in the model, the data sources upon which these were based, and all calculations that were performed to derive these probabilities. All probabilities were converted into 28-day transition probabilities to match the model cycle length. No adjustments were made for gender differences as these were rarely reported. Residual probabilities (R) are not reported in this table, but are indicated in **Appendix One** of this supplementary file. Transition probabilities for death from other causes are not indicated in the table but were informed by age-adjusted UK life table data [5].

**Transition probabilities used in the Markov model**

| **P** | **Disease state** | **Transition** | **TP** | **Notes** | **Source** |
| --- | --- | --- | --- | --- | --- |
| p1 | Undergoing first-line FCR treatment (no *TP53* mutation) | Dead from disease | 0.0027 | The mortality rate for this patient subgroup was reported to be 0.10 (39/386) at three years. This rate was converted into a 28-day transition probability (TP). | [6] |
| p2 | Undergoing first-line FCR treatment (no *TP53* mutation) | No response to treatment – enters second-line treatment | 0.0016 | The non-response rate for this patient subgroup was reported to be 0.08 (32/386) at three years. This was converted to a 28-day TP. It was then assumed that a proportion of those patients who do not respond to treatment are those in whom a high risk genetic mutation has emerged. This TP (p3) was therefore subtracted from the TP calculated from Hallek *et al.* [6]. | [6] |
| p3 | Undergoing first-line FCR treatment (no *TP53* mutation) | High risk genetic mutation emerges – enters refractory therapy | 0.0006 | Both references reported the proportion of patients who acquired a cytogenetic abnormality that changed their risk classification to a higher level. In Shanafelt *et al.* [7] this was 5/108 patients (median time from baseline to second sample 5.6 years). In Gunnarsson *et al.* [8] this was 3/59 patients (median time 6.67 years). This information was used to calculate a 28-day TP for each study (0.0006 in both cases). An unweighted average TP was then calculated. In the absence of any information to the contrary in the literature, the same TP was assumed to apply to patients receiving either FCR or BR treatment, and also patients identified by genomic testing (correctly or otherwise) to be likely FCR responders. | [7, 8] |
| p4 | Undergoing first-line BR treatment (no *TP53* mutation) | Dead from disease | 0.0027 | There is limited good quality data from Phase III clinical trials on first-line BR treatment efficacy in the published literature. That which does exist [9-11] suggests that clinical outcomes will be similar to those achieved by FCR treatment (including the impact of del17p status). Consequently, the TPs for first-line BR treatment were assumed to be the same as those for first-line FCR treatment. | [6, 9-11] |
| p5 | Undergoing first-line BR treatment (no *TP53* mutation) | No response to treatment – enters second-line treatment | 0.0016 | See p4. | [6] |
| p6 | Undergoing first-line BR treatment (no *TP53* mutation) | High risk genetic mutation emerges – enters refractory therapy | 0.0006 | See p3. | [7, 8] |
| p7 | Undergoing second-line FCR treatment (no *TP53* mutation) | Dead from disease | 0.0033 | Robak *et al.* [12] (a Phase III clinical trial) reports a mortality rate for this patient subgroup at a maximum follow-up of 1729 days of 0.23 (62/274). However, this information is not presented by del17p status. Badoux *et al.* [13] do report such information (median overall survival is 10.5 months in del17p patients and 46.5 months in all patients), but this is a Phase II study. This hazard rate was therefore applied to the mortality rate for all patients in Robak *et al.* to generate a mortality rate for del17p patients at 1729 days. From this, the number of patients in Robak *et al.* without del17p who died was calculated, along with a mortality rate (and 28-day TP) for this patient subgroup. | [12, 13] |
| p8 | Undergoing second-line FCR treatment (no *TP53* mutation) | No response to treatment – enters refractory therapy | 0.0049 | Robak *et al.* [12] reports that the non-response rate in all patients receiving FCR as second-line treatment was 0.3007 at maximum follow-up, yielding a 28-day TP of 0.0058. However, this information is again not presented by del17p status. Instead, data from Badoux *et al.* [13] was used to calculate a hazard rate which was applied to the response rate for all patients in Robak *et al.* to generate a response rate for del17p patients. From this, the number of patients in Robak *et al.* who did not respond was calculated, along with a 28-day TP for this patient subgroup.  This 28-day TP incorporates both patients who enter BSC and patients who go on to receive refractory treatment. There is limited information available in the published literature to separate out these two groups. However, it is clear that few patients receive just two courses of FCR or BR before entering BSC [14, 15]. It was therefore assumed that most of these patients (95%) received refractory treatment, with the remaining 5% entering BSC. The 28-day TP was therefore adjusted accordingly. | [12, 13] |
| p9 | Undergoing second-line FCR treatment (no *TP53* mutation) | No response to treatment – enters BSC | 0.0003 | See p8. | [12, 13] |
| p10 | Undergoing second-line BR treatment (no *TP53* mutation) | Dead from disease | 0.0178 | Median survival is reported for various patient subgroups, but not the subgroup of patients without del17p. Therefore a weighted average was taken of median survival in three other patient subgroups (trisomy 12, 13q deletion, no abnormalities) and used to calculate a 28-day TP for the patient subgroup without del17p. | [16] |
| p11 | Undergoing second-line BR treatment (no *TP53* mutation) | No response to treatment – enters refractory therapy | 0.0049 | There is limited good quality data from Phase III clinical trials on second-line BR treatment response rates in the published literature. Therefore, the same assumption was made as for first-line BR treatment, namely that the second-line BR treatment response rate is similar to that achieved by second-line FCR treatment (including the impact of del17p status). Consequently, the TPs for response to second-line BR treatment were assumed to be the same as those for second-line FCR treatment. | [12, 13] |
| p12 | Undergoing second-line BR treatment (no *TP53* mutation) | No response to treatment – enters BSC | 0.0003 | See p11. | [12, 13] |
| p13 | Undergoing refractory Ofa treatment (no *TP53* mutation) | Dead from disease | 0.0288 | Byrd *et al.* [17] reports a 12 month mortality rate of 0.19 for all patients receiving refractory ofatumumab treatment. Wierda *et al.* [18] reports 18 month mortality rates of 0.59 (fludarabine- and alemtuzumab-refractory patients) and 0.54 (bulky fludarabine-refractory patients). In the absence of any better quality data in the published literature for this parameter, an unweighted average TP was calculated across both data sources. This TP was assumed to apply to all patients receiving refractory ofatumumab treatment, regardless of del17p status. | [17, 18] |
| p14 | Undergoing refractory Ofa treatment (no *TP53* mutation) | No response to treatment – enters BSC | 0.0201 | The non-response rate in this patient subgroup was reported to be 0.41 (42/102) at two years. A 28-day TP was calculated based on this figure. | [18] |
| p15 | Undergoing refractory Ofa treatment (no *TP53* mutation) | Undergoing BMT | 0.0174 | There was no data available in the published literature to inform the estimation of this parameter. Clinicians at OUH estimated that 10% of patients treated with ofatumumab would be fit enough to be offered a BMT. This estimate was converted into a 28-day TP, and assumed to apply to all patients, regardless of del17p status. | EO |
| p16 | Undergoing BMT (no *TP53* mutation) | Dead from disease | 0.0082 | Mortality rate at four years reported to be 0.35 in all patients (unaffected by del17p status). A 28-day TP was calculated based on this figure. | [19] |
| p17 | Undergoing BMT (no *TP53* mutation) | Enters BSC | 0.0097 | Relapse incidence at four years reported to be 0.4 (unaffected by del17p status). It was assumed that this represented a reasonable proxy for entering BSC following BMT. A 28-day TP was therefore calculated based on this figure. | [19] |
| p18 | In remission following first-line treatment with FCR/BR (no *TP53* mutation) | Relapse – enters second-line treatment | 0.0084 | Median time to new treatment is reported to be 68.4 months. This information was used to calculate a 28-day TP for all patients (regardless of del17p status). Data on time to new treatment is not presented by del17p status, however data on PFS by del17p status is presented. 28-day TPs were therefore calculated for PFS by del17p status and the ratio of these TPs was used to impute a 28-day TP for the non-response rate in del17p patients. This TP was assumed to apply to patients receiving either FCR or BR. | [6] |
| p19 | In remission following second-line treatment with FCR/BR (no *TP53* mutation) | Relapse – enters refractory treatment | 0.0185 | The proportion of all patients who had progressed at 49 months was reported by Wierda *et al.* [20] (see p53). No information was provided on progression rates by del17p status. However, Robak *et al.* [12] and Badoux *et al.* [13] provide information on overall survival and treatment response rates which can be used to calculate 28-day TPs for these two variables by del17p status (see p7, p8 and p9 for further details). Hazard rates were calculated for no del17p patients versus all patients for each of these 28-day TPs, an unweighted average was calculated, and this was applied to p53 to generate this TP. This TP was assumed to apply to patients receiving either FCR or BR. | [12, 13, 20] |
| p20 | In remission following refractory treatment with Ofa (no *TP53* mutation) | Relapse – enters BSC | 0.0737 | The median time to new treatment is reported to be 8.5 months (fludarabine- and alemtuzumab-refractory patients) and 8.2 months (bulky fludarabine-refractory patients). A weighted average of the 28-day TPs derived from this information was calculated. As no differences by del17p status were noted, this TP was used for all patients regardless of del17p status. | [18] |
| p21 | Receiving best-supportive care (no *TP53* mutation) | Dead from disease | 0.0753 | The NICE submission for the use of ofatumumab in patients refractory to fludarabine and alemtuzumab also reported survival data for patients receiving best supportive care. This information was presented as a Weibull survival function, which was used to calculate a 28-day TP. In the absence of any data to the contrary in the published literature, this probability was assumed to apply to all patients receiving BSC, regardless of previous treatments of del17p status. | [21] |
| p22 | Undergoing first-line Ofa treatment (*TP53* mutation) | Dead from disease | 0.0288 | As there is no trial data available for Ofa monotherapy in treatment naïve patients, the same outcomes were assumed as for relapsed/refractory patients. See p13 for further details. | [17, 18] |
| p23 | Undergoing first-line Ofa treatment (*TP53* mutation) | No response to treatment – enters refractory therapy | 0.0440 | As there is no trial data available for Ofa monotherapy in treatment naïve patients, the same outcomes were assumed as for relapsed/refractory patients. See p29 for further details. As per p8, it was assumed that a small proportion of these patients will enter BSC rather than refractory treatment, hence p29 is adjusted accordingly. | [18] |
| p24 | Undergoing first-line Ofa treatment (*TP53* mutation) | Undergoing BMT | 0.0174 | See p15. | EO |
| p25 | Undergoing first-line Ofa treatment (*TP53* mutation) | No response to treatment – enters BSC | 0.0023 | As there is no trial data available for Ofa monotherapy in treatment naïve patients, the same outcomes were assumed as for relapsed/refractory patients. See p29 for further details. As per p8, it was assumed that a small proportion of these patients will enter BSC rather than refractory treatment, hence p29 is adjusted accordingly. | [18] |
| p26 | Undergoing BMT (*TP53* mutation) | Dead from disease | 0.0082 | See p16. | [19] |
| p27 | Undergoing BMT (*TP53* mutation) | Enters BSC | 0.0097 | See p17. | [19] |
| p28 | Undergoing refractory Ofa treatment (*TP53* mutation) | Dead from disease | 0.0288 | See p13. | [17, 18] |
| p29 | Undergoing refractory Ofa treatment (*TP53* mutation) | No response to treatment – enters BSC | 0.0463 | The non-response rate in this patient subgroup was reported to be 0.71 (22/31) at two years. A 28-day TP was calculated based on this figure. | [18] |
| p30 | In remission following first-line treatment with Ofa (*TP53* mutation) | Relapse – enters refractory treatment | 0.0737 | As there is no trial data available for Ofa monotherapy in treatment naïve patients, the same outcomes were assumed as for relapsed/refractory patients. See p31 for further details. | [18] |
| p31 | In remission following refractory treatment with Ofa (*TP53* mutation) | Relapse – enters BSC | 0.0737 | See p20. | [18] |
| p32 | Receiving best-supportive care (*TP53* mutation) | Dead from disease | 0.0753 | See p21. | [21] |
| p33 | Undergoing refractory IB treatment (no *TP53* mutation) | Dead from disease | 0.0032 | The mortality rate for this patient subgroup at 30 months was reported to be 0.10. This was converted into a 28-day TP. | [22] |
| p34 | Undergoing refractory IB treatment (no *TP53* mutation) | No response to treatment – enters BSC | 0.0043 | This TP was assumed to be equal to the 28-day TP of progression whilst undergoing IB treatment. Byrd *et al.* [22] reports that this rate of progression was 0.13 at 30 months for this patient subgroup. This was converted into a 28-day TP. | [22] |
| p35 | Undergoing refractory IB treatment (*TP53* mutation) | Dead from disease | 0.0131 | The mortality rate for this patient subgroup at 30 months was reported to be 0.35. This was converted into a 28-day TP. | [22] |
| p36 | Undergoing refractory IB treatment (*TP53* mutation) | No response to treatment – enters BSC | 0.0223 | This TP was assumed to be equal to the 28-day TP of progression whilst undergoing IB treatment. Byrd *et al.* [22] reports that this rate of progression was 0.52 at 30 months for this patient subgroup. This was converted into a 28-day TP. | [22] |
| p37 | Undergoing first-line FCR treatment (unknown *TP53* mutation status) | Dead from disease | 0.0035 | The mortality rate for this patient subgroup was reported to be 0.13 (53/408) at three years. This was converted into a 28-day TP. | [6] |
| p38 | Undergoing first-line FCR treatment (unknown *TP53* mutation status) | No response to treatment – enters second-line treatment | 0.0026 | The non-response rate for this patient subgroup was reported to be 0.10 (39/408) at three years. This was converted to a 28-day TP. | [6] |
| p39 | Undergoing first-line BR treatment (unknown *TP53* mutation status) | Dead from disease | 0.0035 | See p4. | [6] |
| p40 | Undergoing first-line BR treatment (unknown *TP53* mutation status) | No response to treatment – enters second-line treatment | 0.0026 | See p4. | [6] |
| p41 | Undergoing second-line FCR treatment (unknown *TP53* mutation status) | Dead from disease | 0.0041 | The mortality rate after 1729 days was reported to be 0.23 (62/274). This was converted to a 28-day TP. | [12] |
| p42 | Undergoing second-line FCR treatment (unknown *TP53* mutation status) | No response to treatment – enters refractory therapy | 0.0055 | See p8. | [12, 13] |
| p43 | Undergoing second-line FCR treatment (unknown *TP53* mutation status) | No response to treatment – enters BSC | 0.0003 | See p8. | [12, 13] |
| p44 | Undergoing second-line BR treatment (unknown *TP53* mutation status) | Dead from disease | 0.0186 | Median survival for all patients was reported to be 33.9 months. This was converted into a 28-day TP. | [16] |
| p45 | Undergoing second-line BR treatment (unknown *TP53* mutation status) | No response to treatment – enters refractory therapy | 0.0055 | See p11. | [12, 13] |
| p46 | Undergoing second-line BR treatment (unknown *TP53* mutation status) | No response to treatment – enters BSC | 0.0003 | See p11. | [12, 13] |
| p47 | Undergoing refractory Ofa treatment (unknown *TP53* mutation status) | Dead from disease | 0.0288 | See p13. | [17, 18] |
| p48 | Undergoing refractory Ofa treatment (unknown *TP53* mutation status) | No response to treatment – enters BSC | 0.0250 | Wierda *et al.* [18] reports two-yearly non-response rates of 0.42 (fludarabine- and alemtuzumab-refractory patients) and 0.53 (bulky fludarabine-refractory patients). A weighted average of these non-response rates was used to calculate a 28-day TP. | [18] |
| p49 | Undergoing refractory Ofa treatment (unknown *TP53* mutation status) | Undergoing BMT | 0.1000 | See p15. | EO |
| p50 | Undergoing BMT (unknown *TP53* mutation status) | Dead from disease | 0.0082 | See p16. | [19] |
| p51 | Undergoing BMT (unknown *TP53* mutation status) | Enters BSC | 0.0097 | See p17. | [19] |
| p52 | In remission following first-line treatment with FCR/BR (unknown *TP53* mutation status) | Relapse – enters second-line treatment | 0.0093 | Median time to new treatment is reported to be 68.4 months. This information was used to calculate a 28-day TP for all patients (regardless of del17p status). This TP was assumed to apply to patients receiving either FCR or BR. | [6] |
| p53 | In remission following second-line treatment with FCR/BR (unknown *TP53* mutation status) | Relapse – enters refractory treatment | 0.0218 | The proportion of all patients who had progressed at 49 months was reported to be 0.69. This figure was converted into a 28-day TP. This TP was assumed to apply to patients receiving either FCR or BR. | [20] |
| p54 | In remission following refractory treatment with Ofa (unknown *TP53* mutation status) | Relapse – enters BSC | 0.0737 | See p20. | [18] |
| p55 | Receiving best-supportive care (unknown *TP53* mutation status) | Dead from disease | 0.0753 | See p21. | [21] |
| p56 | Undergoing first-line FCR treatment (patient correctly identified by genomic testing to be an FCR responder) | Dead from disease | 0.0022 | It was assumed that the efficacy of FCR treatment would be improved in this patient subgroup due to better targeting of treatment. The mortality rate at three years estimated by Hallek *et al.* [6] was therefore applied for patients without del17p, reduced by 20% to 0.08. This was converted to a 28-day TP. | [6] |
| p57 | Undergoing first-line FCR treatment (patient correctly identified by genomic testing to be an FCR responder) | No response to treatment – enters second-line treatment | 0.0018 | It was assumed that the efficacy of FCR treatment would be improved in this patient subgroup due to better targeting of treatment. The non-response rate at three years estimated by Hallek *et al.* [6] was therefore applied for patients without del17p, reduced by 20% to 0.07. This was converted to a 28-day TP. | [6] |
| p58 | Undergoing first-line FCR treatment (patient correctly identified by genomic testing to be an FCR responder) | Further genomic changes emerge – enters refractory therapy | 0.0006 | See p3. | [7, 8] |
| p59 | Undergoing first-line FCR treatment (patient incorrectly identified by genomic testing to be an FCR responder) | Dead from disease | 0.0244 | In the absence of any data in the published literature, it was assumed that this TP was equal to that for mortality in del17p patients receiving FCR as first-line treatment. The survival rate in this patient subgroup was reported to be 0.38 (8/22). This was converted to a 28-day TP. | [6] |
| p60 | Undergoing first-line FCR treatment (patient incorrectly identified by genomic testing to be an FCR responder) | No response to treatment – enters second-line treatment | 0.0097 | In the absence of any data in the published literature, it was assumed that this TP was equal to that for non-response in del17p patients receiving FCR as first-line treatment. The non-response rate in this patient subgroup was reported to be 0.32 (7/22). This was converted to a 28-day TP. | [6] |
| p61 | Undergoing first-line FCR treatment (patient incorrectly identified by genomic testing to be an FCR responder) | Further genomic changes emerge – enters refractory therapy | 0.0006 | See p3. | [7, 8] |
| p62 | Undergoing second-line BR treatment (patient identified by genomic testing to be a likely FCR responder) | Dead from disease | 0.0128 | It was assumed that the efficacy of BR treatment would be improved in this patient subgroup due to better targeting of treatment. An estimate of median survival was derived in the same way as for p10, which was then increased by 20%. This was converted to a 28-day TP. | [16] |
| p63 | Undergoing second-line BR treatment (patient identified by genomic testing to be a likely FCR responder) | No response to treatment – enters refractory therapy | 0.0028 | It was assumed that the efficacy of BR treatment would be improved in this patient subgroup due to better targeting of treatment. An estimate of the non-response rate was derived in the same way as for p8 and p9. This was then decreased by 20%. This was converted to a 28-day TP. | [12, 13] |
| p64 | Undergoing second-line BR treatment (patient identified by genomic testing to be a likely FCR responder) | No response to treatment – enters BSC | 0.0012 | See p63. | [12, 13] |
| p65 | Undergoing refractory IB treatment (patient identified by genomic testing to be a likely FCR responder) | Dead from disease | 0.0032 | In the absence of any data in the published literature, it was assumed that the mortality rate in patients without del17p was a reasonable proxy for the mortality rate in likely FCR responders. See p33 for further information. | [22] |
| p66 | Undergoing refractory IB treatment (patient identified by genomic testing to be a likely FCR responder) | No response to treatment – enters BSC | 0.0043 | In the absence of any data in the published literature, it was assumed that the non-response rate in patients without del17p was a reasonable proxy for the mortality rate in likely FCR responders. See p34 for further information. | [22] |
| p67 | In remission following first-line treatment with FCR (patient correctly identified by genomic testing to be an FCR responder) | Relapse – enters second-line treatment | 0.0070 | It was assumed that the efficacy of FCR treatment would be improved in this patient subgroup due to better targeting of treatment. The median time to new treatment estimated by Hallek *et al.* [6] was therefore applied for patients without del17p, increased by 20% to 2754 days. This was converted to a 28-day TP. | [6] |
| p68 | In remission following first-line treatment with FCR (patient incorrectly identified by genomic testing to be an FCR responder) | Relapse – enters second-line treatment | 0.0361 | In the absence of any data in the published literature, it was assumed that this TP was equal to that for time to new treatment following FCR in del17p patients. See p18 for further details. | [6] |
| p69 | In remission following second-line treatment with BR (patient identified by genomic testing to be a likely FCR responder) | Relapse – enters refractory treatment | 0.0127 | It was assumed that the efficacy of BR treatment would be improved in this patient subgroup. The rate of progression used in p53 was therefore applied, reduced by 20% to 0.55. This was then converted to a 28-day TP. | [20] |
| p70 | Receiving best-supportive care (patient identified by genomic testing to be a likely FCR responder) | Dead from disease | 0.0753 | See p21. | [21] |
| p71 | Undergoing first-line IB treatment (patient identified by genomic testing to be a likely FCR non-responder) | Dead from disease | 0.0025 | There is very limited data in the published literature on the efficacy of first-line IB treatment. O’Brien *et al.* [23] reports such data for a small group of elderly patients (who may be a reasonable proxy for likely FCR non-responders) estimating that the proportion of patients who are dead at 24 months is 0.034. Farooqui *et al.* [24] reports such data for a small group of patients with del17p, estimating that the proportion of patients who are dead at 24 months is 0.09. Given the limitations associated with the two datasets, 28-day TPs were calculated for both and an unweighted average of these two TPs was used. | [23, 24] |
| p72 | Undergoing first-line IB treatment (patient identified by genomic testing to be a likely FCR non-responder) | No response to treatment – enters BSC | 0.0041 | This TP was estimated using the same studies as p71. The progression rate at 24 months is reported to be 0.037 (O’Brien *et al.* [23]) and 0.16 (Farooqui *et al.* [24]). 28-day TPs were calculated for both studies and an unweighted average used. | [23, 24] |
| p73 | Receiving best-supportive care (patient identified by genomic testing to be a likely FCR non-responder) | Dead from disease | 0.0753 | See p21. | [21] |

BMT = bone marrow transplant; BR = bendamustine and rituximab; BSC = best supportive care; EO = expert opinion; FCR = fludarabine, cyclophosphamide and rituximab; IB = ibrutinib; NICE = National Institute for Health and Care Excellence; Ofa = ofatumumab; OUH = Oxford University Hospitals NHS Trust; P = Probability; PFS = progression-free survival; TP = 28-day transition probability.

# Appendix Five: Unit costs

| **Resource use item** | **Unit cost** | **Notes** | **Source** |
| --- | --- | --- | --- |
| *Chemotherapy drugs ^a^* |  |  |  |
| FCR (first cycle) | £2,224.87 | Cost per 28-day cycle. Fludarabine: 40mg/m^2^ for five days of cycle. Cyclophosphamide: 250mg/m^2^ per day for first three days of cycle. Rituximab: 375mg/m^2^ on day zero of cycle | [25, 26] |
| FCR (subsequent cycles) | £2,574.13 | Cost per 28-day cycle. Fludarabine: 40mg/m^2^ for five days each cycle. Cyclophosphamide: 250mg/m^2^ per day for first three days of cycle. Rituximab: 500mg/m^2^ on day one of cycle | [25, 26] |
| BR (first cycle) | £2,504.26 | Cost per 28-day cycle. Bendamustine: 100mg/m^2^ on days one and two of cycle. Rituximab: 375mg/m^2^ on day zero of cycle | [25] |
| BR (subsequent cycles) | £2,853.52 | Cost per 28-day cycle. Bendamustine: 100mg/m^2^ on days one and two of cycle. Rituximab: 500mg/m^2^ on day one of cycle | [25] |
| Ofatumumab (first cycle) | £11,466.00 | Cost per 28-day cycle. One infusion per week (infusion one is 300mg, infusions 2-4 are 2,000mg). For ofatumumab with high dose methylprednisolone, add £105.80. For ofatumumab with dexamethasone, add £3.85 | [25, 26] |
| Ofatumumab (second cycle) | £14,560.00 | Cost per 28-day cycle. One 2,000mg infusion per week. For ofatumumab with high dose methylprednisolone, add £105.80. For ofatumumab with dexamethasone, add £3.85 | [25, 26] |
| Ofatumumab (subsequent cycles) | £3,640.00 | Cost per 28-day cycle. One 2,000mg infusion per cycle. For ofatumumab with high dose methylprednisolone, add £105.80. For ofatumumab with dexamethasone, add £3.85 | [25, 26] |
| Ibrutinib (all cycles) | £2,299.79 | Cost per 28-day cycle. Daily dose 420mg. Assumed that annual cost of treatment would not exceed £30,000, so daily cost assumed to be £82.14 | Ass |
| Treatment of complications arising from chemotherapy | £2,776.70 | An unweighted average was taken of the costs of seven potential complications arising from chemotherapy. Further information provided in **Appendix Seven**. | [25, 27, 28] |
| *Other drugs ^a^* |  |  |  |
| Co-trimoxazole | £0.19 | Cost per 960mg dose (given three times a week) | [26] |
| Fluconazole | £0.03 | Cost per 50mg dose (given daily) | [26] |
| Aciclovir | £0.03 | Cost per 200mg dose (given three times a day) | [26] |
| GCSF | £55.78 | Cost per 300mcg injection (given daily) | [26] |
| Hydrocortisone | £1.08 | Cost per 100mg injection (given daily) | [26] |
| Chlorpheniramine | £0.06 | Cost per 30mg dose (given daily) | [26] |
| Paracetamol | £0.01 | Cost per 1g dose (given daily) | [26] |
| Ganciclovir | £29.77 | Cost per 5mg/kg IV infusion (given twice daily) | [25] |
| IV immunoglobulin treatment | £1,201.23 | Immunoglobulins, Band 1, weighted average (Currency code: XD34Z) | [28] |
| *Surgeries, tests and procedures ^b^* |  |  |  |
| Genetic test | £281.08 | Calculated based on clinical practice in OUH | Calc |
| Genomic test | £319.23 | Calculated based on clinical practice in OUH | Calc |
| FBC | £3.01 | Haematology test (Currency code: DAPS05) | [28] |
| U&E | £1.25 | Clinical biochemistry test (Currency code: DAPS04) | [28] |
| LFT | £1.25 | Clinical biochemistry test (Currency code: DAPS04) | [28] |
| CT scan | £106.32 | Average of all types of CT scan, weighted by activity | [28] |
| IgV_H_ test | £38.74 | Histopathology and histology test (Currency code: DAPS02) | [28] |
| Lung function test | £40.61 | Simple lung function test (Currency code: DZ32Z) | [28] |
| Diagnostic bone marrow extraction | £482.48 | Clinical haematology (Currency code: SA33Z) | [28] |
| Thyroid function test | £1.25 | Clinical biochemistry test (Currency code: DAPS04) | [28] |
| Immunoglobulin test | £3.01 | Haematology test (Currency code: DAPS05) | [28] |
| Blood transfusion | £381.88 | This cost reflects 2.10 units of CMV negative and irradiated red cells, and one haematology appointment (non-consultant) | [21, 28, 29] |
| Blood and platelet support | £381.88 | This cost was assumed to be the same as that for a blood transfusion | [21, 28, 29] |
| BMT | £43,723.96 | Average of all allogeneic graft categories (Currency codes SA20A, SA21A, SA22A), weighted by activity | [28] |
| Treatment of complications arising from BMT | £1,842.66 | An unweighted average was taken of the costs of three potential complications post-BMT. Further information provided in **Appendix Eight**. | [25, 28] |
| Donor lymphocyte infusion | £381.88 | Assumed to be the same cost as a blood transfusion, as per a previous study in leukaemia | [21, 28-30] |
| *Staff costs ^b^* |  |  |  |
| Haematology appointment (consultant) | £142.71 | Currency code WF01A: Non-Admitted Face to Face Attendance, Follow-up - Clinical Haematology. Service code: 303 (Consultant led outpatient attendances) | [28] |
| Haematology appointment (non-consultant) | £90.72 | Currency code WF01A: Non-Admitted Face to Face Attendance, Follow-up - Clinical Haematology. Service code: 303 (Non consultant led outpatient attendances) | [28] |
| Telephone consultation with haematology nurse | £60.77 | Currency code WF01C: Non-Admitted Non-Face to Face Attendance, Follow-up - Clinical Haematology. Service code: 303 (Non consultant led outpatient attendances) | [28] |
| Immunology appointment (consultant) | £195.01 | Currency code WF01A: Non-Admitted Face to Face Attendance, Follow-up - Clinical Immunology. Service code: 316 (Consultant led outpatient attendances) | [28] |
| *Miscellaneous ^b^* |  |  |  |
| Pharmacy costs for dispensing chemotherapy drugs | £10.25 | 15min of hospital pharmacist time | [21, 31] |
| Deliver exclusively oral chemotherapy | £162.06 | Currency code SB11Z. Weighted average of all service codes (DCRDN, OP, Oth) | [28] |
| Deliver more complex parenteral chemotherapy at first attendance | £261.71 | Currency code SB13Z. Weighted average of all service codes (DCRDN, OP, Oth) | [28] |
| Deliver complex chemotherapy, including prolonged infusional treatment, at first attendance | £319.62 | Currency code SB14Z. Weighted average of all service codes (DCRDN, OP, Oth) | [28] |
| Deliver subsequent elements of a chemotherapy cycle | £291.64 | Currency code SB15Z. Weighted average of all service codes (DCRDN, OP, Oth) | [28] |
| Inpatient stay | £2,577.16 | Currency code SA32D: Chronic Lymphocytic Leukaemia and Related Disorders, with CC Score 0-2. Service code 303. Non-elective long stay. | [28] |

Ass = assumption; BMT = bone marrow transplant; BR = bendamustine and rituximab; Calc = calculated; CMV = Cytomegalovirus; CT = computerised tomography; FBC = full blood count; FCR = fludarabine, cyclophosphamide and rituximab; GCSF = granulocyte colony-stimulating factor; IgV_H_ = immunoglobulin heavy chain variable region; LFT = liver function test; OUH = Oxford University Hospitals NHS Trust; U&E = urea and electrolytes test. ^a^ Dosages are based on an adult with height 168cm and weight 77kg (yielding a body surface area of 1.88m^2^) [32]. ^b^ All costs informed by NHS Reference Costs are based on patients aged over 18 years.

# Appendix Six: Complication rates

| **Treatment** | **Line of therapy / patient subgroup** | **Average number of complications experienced per 28 day cycle of treatment** | **Notes** | **Sources** |
| --- | --- | --- | --- | --- |
| FCR | First-line | 0.3489 | 733 grade 3/4 events experienced across 2101 treatment courses | [6] |
|  | Second-line | 0.6978 | No information was available in the literature on this parameter. Clinical experience at OUH NHS Trust suggests that the complication rate doubles with each line of treatment. The first-line complication rate was therefore doubled | Ass |
|  | First-line – FCR responders | 0.1745 | No information was available in the literature on this parameter. It was therefore assumed that the complication rate would halve due to improved targeting of treatment | Ass |
| BR | First-line | 0.3626 | No information was available in the literature on this parameter. Clinical experience at OUH NHS Trust suggests that the complication rate doubles with each line of treatment. The second-line complication rate was therefore halved | Ass |
|  | Second-line | 0.7252 | 256 grade 3/4 events experienced across 353 treatment courses | [16] |
|  | Second-line – FCR responders | 0.3626 | No information was available in the literature on this parameter. It was therefore assumed that the complication rate would halve due to improved targeting of treatment | Ass |
| Ofa | First-line | 0.0922 | No information was available in the literature on this parameter. Clinical experience at OUH NHS Trust suggests that the complication rate doubles with each line of treatment. The second-line complication rate was therefore halved | Ass |
|  | Second- or third-line | 0.1843 | Byrd *et al.* [17] report 82 grade 3/4 events experienced across approximately 1100 treatment courses. A second study [21] reports 85 grade 3/4 events experienced across approximately 289 treatment courses. An unweighted average was taken of these two complication rates | [17, 21] |
| IB | Third-line | 0.0581 | 106 grade 3/4 events experienced across approximately 1823 treatment courses | [17] |
|  | Third-line – FCR responders | 0.0291 | No information was available in the literature on this parameter. It was therefore assumed that the complication rate would halve due to improved targeting of treatment | Ass |
|  | First-line – FCR non-responders | 0.0291 | No information was available in the literature on this parameter. It was therefore assumed that the complication rate would be the same as that in FCR responders receiving third-line IB treatment | Ass |
| BMT | - | 0.0195 | This complication rate was estimated based on clinical experience at OUH NHS Trust, which suggested that 80% of patients experience one complication whilst in remission (an average of 51 28-day cycles) | Ass |

Ass = assumption; BMT = bone marrow transplant; BR = bendamustine and rituximab; FCR = fludarabine, cyclophosphamide and rituximab; IB = ibrutinib; Ofa = ofatumumab; OUH = Oxford University Hospitals.

# Appendix Seven: Costs associated with treating chemotherapy complications

Note: A variety of complications can be experienced by CLL patients following chemotherapy treatment. Grade one and two complications (e.g. chills) were not costed as either no healthcare resources are used to treat these complications, or patients self-medicate [33]. Given this, only grade three and four complications were included in this analysis. These complication rates were estimated using the data sources which informed the transition probabilities, but were not estimated separately for different genetic subgroups as it is generally accepted that complication rates are independent of genetic subgroups. **Appendix Six** presents these complication rates. The most common grade three and four complications (and the treatments given for these complications) were predicted by clinicians in the Oxford University Hospitals NHS Trust, informed by clinical experience. These complications match those outlined in the latest UK CLL guidelines [34]. **T**he costs associated with treating these complications are presented in the table below. These unit costs were used to calculate an average cost for a grade three or four complication, which was combined with the data on average complication rates to calculate an average cost of complications for each health state.

| **Chemotherapy complication** | **Unit cost** | **Notes** | **Source** |
| --- | --- | --- | --- |
| Anaemia | £2,254 | Currency codes SA03G/H: haemolytic anaemia. Average used of the costs for clinical haematology, weighted by activity | [28] |
| CMV reactivation | £1,042 | Ganciclovir treatment. Assumed an average of 35 infusions per course of treatment | [25] |
| Dyspnoea | £3,409 | Currency codes DZ21A/M/Q/R/S/T/U: chronic obstructive pulmonary disease or bronchitis. Average used of the costs for clinical haematology, weighted by activity | [27, 28] |
| Febrile neutropenia | £4,009 | In the absence of specific cost data, this cost was assumed to be equal to that for infections (see below) | [28] |
| Fever | £1,608 | Currency code WA04Z: acute febrile illness with length of stay 4 days or less – clinical haematology | [28] |
| Infection (including pneumonia) | £4,009 | Currency codes DZ11-J (lobar, atypical or viral pneumonia) and WA03A-C (septicaemia). Average used of the costs for clinical haematology, weighted by activity | [28] |
| Thrombocytopenia | £3,106 | Currency code SA12G-K: thrombocytopenia. Average used of the costs for clinical haematology, weighted by activity | [28] |
| **Unweighted average cost** | **£2,777** |  |  |

CMV = cytomegalovirus.

# Appendix Eight: Costs associated with treating bone marrow transplant complications

Note: The costs associated with complications experienced by patients after BMT were calculated in a similar way to the costs associated with chemotherapy complications. Again, the most common complications (and the treatments given for these complications) were predicted by clinicians in the Oxford University Hospitals NHS Trust, informed by clinical experience.

| **BMT complication** | **Unit cost** | **Notes** | **Source** |
| --- | --- | --- | --- |
| Infection (including pneumonia) | £4,009 | Currency codes DZ11-J (lobar, atypical or viral pneumonia) and WA03A-C (septicaemia). Average used of the costs for clinical haematology, weighted by activity | [28] |
| Chronic graft versus host disease | £477 | Unweighted average cost calculated for three treatment options: (1) Steroids – 2mg/kg of methylprednisolone daily for 28 days; (2) PUVA light therapy; (3) Photopheresis (Currency code for (2) and (3): JC47A) | [28] |
| CMV reactivation | £1,042 | Ganciclovir treatment. Assumed an average of 35 infusions per course of treatment | [25] |
| **Unweighted average cost** | **£1,843** |  |  |

BMT = bone marrow transplant; CMV = cytomegalovirus.

# Appendix Nine: Average length of chemotherapy treatment

Note: Some of the resource use calculations required information on the average length of treatment for different types of chemotherapy. This data was extracted from the data sources which informed the transition probabilities and is presented below. Average length of treatment was not estimated separately for different genetic subgroups as this information was rarely available.

| **Disease state** | **Average number of 28-day cycles spent in state** | **Notes** | **Source** |
| --- | --- | --- | --- |
| Undergoing FCR treatment | 5.2 | Mean number of treatment courses | [6] |
| Undergoing BR treatment | 4.5 | Mean number of treatment courses | [16] |
| Undergoing Ofa treatment | 5.8 | Median treatment exposure was reported to be 5.3 months | [17] |
| Undergoing IB treatment | 9.3 | Median treatment exposure was reported to be 8.6 months | [17] |
| In remission following FCR treatment | 56.3 | Median PFS was reported to be 51.8 months | [6] |
| In remission following BMT | 51.3 | The relapse incidence at 4 years following BMT was reported to be 0.4. This was converted into a 28-day transition probability of 0.0097 (see p17, p27, p51, p60 in **Appendix Four**). This transition probability suggests that it would take 102.6 28-day cycles for all patients to exit remission following BMT. It was assumed that half of all patients would have exited this state exactly halfway through this period i.e. after 51.3 cycles | [19] |

BMT = bone marrow transplant; BR = bendamustine and rituximab; BSC = best supportive care; FCR = fludarabine, cyclophosphamide and rituximab; IB = ibrutinib; Ofa = ofatumumab; PFS = progression-free survival.

# Appendix Ten: Resource use and overall costs for each health state

| **State** | **Item ^a^** | **Notes ^b^** | **Cost (£)** |
| --- | --- | --- | --- |
| FCR treatment (first-line, first cycle) | Genetic test (Comparator A/B only) | - | 281.08 |
|  | Genomic test (Intervention 1/2 only) | - | 319.23 |
|  | FCR treatment | - | 2554.74 |
|  | Co-trimoxazole treatment | 960mg three times a week | 2.29 |
|  | Fluconazole treatment | 50mg daily | 0.76 |
|  | Aciclovir treatment | 200mg three times a day | 2.59 |
|  | GCSF treatment | 60% of patients receive 300mcg daily for seven days of cycle | 234.26 |
|  | FBC | One test per cycle | 3.01 |
|  | U&E | One test per cycle | 1.25 |
|  | LFT | One test per cycle | 1.25 |
|  | CT scan | One scan per cycle | 106.32 |
|  | Blood transfusion | 20% of patients have one transfusion over all cycles of FCR treatment. Average treatment length is 5.2 cycles, so 0.19 transfusions per cycle were costed | 14.69 |
|  | Outpatient appointment with haematologist | One appointment per cycle | 142.71 |
|  | Registrar appointment prior to treatment commencing | One appointment per cycle | 90.72 |
|  | Complications (Comparator A/B/C) | See **Appendix Six** for more details of complication rates | 968.83 |
|  | Complications (Intervention 1/2) | See **Appendix Six** for more details of complication rates | 484.42 |
|  |  | **TOTAL (Comparator A/B)** | **4404.48** |
|  |  | **TOTAL (Comparator C)** | **4123.40** |
|  |  | **TOTAL (Intervention 1/2)** | **3958.22** |
| FCR treatment (second-line, first cycle) | Genetic test (Comparator A/B only) |  | 281.08 |
|  | FCR treatment | - | 2554.74 |
|  | Co-trimoxazole treatment | 960mg three times a week | 2.29 |
|  | Fluconazole treatment | 50mg daily | 0.76 |
|  | Aciclovir treatment | 200mg three times a day | 2.59 |
|  | GCSF treatment | 60% of patients receive 300mcg daily for seven days of cycle | 234.26 |
|  | FBC | One test per cycle | 3.01 |
|  | U&E | One test per cycle | 1.25 |
|  | LFT | One test per cycle | 1.25 |
|  | CT scan | One scan per cycle | 106.32 |
|  | Blood transfusion | 20% of patients have one transfusion over all cycles of FCR treatment. Average treatment length is 5.2 cycles, so 0.19 transfusions per cycle were costed | 14.69 |
|  | Outpatient appointment with haematologist | One appointment per cycle | 142.71 |
|  | Registrar appointment prior to treatment commencing | One appointment per cycle | 90.72 |
|  | Complications | See **Appendix Six** for more details of complication rates | 1937.66 |
|  |  | **TOTAL (Comparator A/B)** | **5373.31** |
|  |  | **TOTAL (Comparator C)** | **5092.23** |
| FCR treatment (first- or second-line, subsequent cycles) | FCR treatment | - | 2876.02 |
|  | Co-trimoxazole treatment | 960mg three times a week | 2.29 |
|  | Fluconazole treatment | 50mg daily | 0.76 |
|  | Aciclovir treatment | 200mg three times a day | 2.59 |
|  | GCSF treatment | 60% of patients receive 300mcg daily for seven days of cycle | 234.26 |
|  | FBC | One test per cycle | 3.01 |
|  | U&E | One test per cycle | 1.25 |
|  | LFT | One test per cycle | 1.25 |
|  | CT scan | One scan is given at the end of the full course of FCR treatment. Average treatment length is 4.2 cycles (excluding cycle 1), so 0.24 scans per cycle were costed | 25.31 |
|  | Blood transfusion | 20% of patients have one transfusion over all cycles of FCR treatment. Average treatment length is 5.2 cycles, so 0.19 transfusions per cycle were costed | 14.69 |
|  | Outpatient appointment with haematologist | After cycle one, patients have an outpatient appointment halfway through the treatment course and another at the end. Average treatment length is 4.2 cycles (excluding cycle one), so 0.48 appointments per cycle were costed | 67.96 |
|  | Registrar appointment prior to treatment commencing | One appointment per cycle | 90.72 |
|  | Complications (Comparator A/B/C, first-line treatment) | See **Appendix Six** for more details of complication rates | 968.83 |
|  | Complications (Comparator A/B/C, second-line treatment) | See **Appendix Six** for more details of complication rates | 1937.66 |
|  | Complications (Intervention 1/2) | See **Appendix Six** for more details of complication rates | 484.42 |
|  |  | **TOTAL (Comparator A/B/C, first-line)** | **4288.92** |
|  |  | **TOTAL (Comparator A/B/C, second-line)** | **5257.75** |
|  |  | **TOTAL (Intervention 1/2)** | **3804.50** |
| BR treatment (first-line, first cycle) | Genetic test (Comparator A/B only) | - | 281.08 |
|  | BR treatment | - | 2854.63 |
|  | GCSF treatment | 10% of patients receive 300mcg daily for seven days of cycle | 39.04 |
|  | FBC | One test per cycle | 3.01 |
|  | U&E | One test per cycle | 1.25 |
|  | LFT | One test per cycle | 1.25 |
|  | CT scan | One scan per cycle | 106.32 |
|  | Blood transfusion | 10% of patients have one transfusion over all cycles of BR treatment. Average treatment length is 4.5 cycles, so 0.22 transfusions per cycle were costed | 8.44 |
|  | Outpatient appointment with haematologist | One appointment per cycle | 142.71 |
|  | Registrar appointment prior to treatment commencing | One appointment per cycle | 90.72 |
|  | Complications | See **Appendix Six** for more details of complication rates | 1006.85 |
|  |  | **TOTAL (Comparator A/B)** | **4535.29** |
|  |  | **TOTAL (Comparator C)** | **4254.21** |
| BR treatment (second-line, first cycle) | Genetic test (Comparator A/B only) | - | 281.08 |
|  | Genomic test (Intervention 1/2 only) | - | 319.23 |
|  | BR treatment | - | 2854.63 |
|  | GCSF treatment | 10% of patients receive 300mcg daily for seven days of cycle | 39.04 |
|  | FBC | One test per cycle | 3.01 |
|  | U&E | One test per cycle | 1.25 |
|  | LFT | One test per cycle | 1.25 |
|  | CT scan | One scan per cycle | 106.32 |
|  | Blood transfusion | 10% of patients have one transfusion over all cycles of BR treatment. Average treatment length is 4.5 cycles, so 0.22 transfusions per cycle were costed | 8.44 |
|  | Outpatient appointment with haematologist | One appointment per cycle | 142.71 |
|  | Registrar appointment prior to treatment commencing | One appointment per cycle | 90.72 |
|  | Complications (Comparator A/B/C) | See **Appendix Six** for more details of complication rates | 2013.69 |
|  | Complications (Intervention 1/2) | See **Appendix Six** for more details of complication rates | 1006.85 |
|  |  | **TOTAL (Comparator A/B)** | **5542.13** |
|  |  | **TOTAL (Comparator C)** | **5261.05** |
|  |  | **TOTAL (Intervention 1/2)** | **4573.44** |
| BR treatment (first- or second-line, subsequent cycles) | BR treatment | - | 2854.63 |
|  | GCSF treatment | 10% of patients receive 300mcg daily for seven days of cycle | 39.04 |
|  | FBC | One test per cycle | 3.01 |
|  | U&E | One test per cycle | 1.25 |
|  | LFT | One test per cycle | 1.25 |
|  | CT scan | One scan is given at the end of the full course of FCR treatment. Average treatment length is 3.5 cycles (excluding cycle 1), so 0.28 scans per cycle were costed | 30.16 |
|  | Blood transfusion | 10% of patients have one transfusion over all cycles of BR treatment. Average treatment length is 4.5 cycles, so 0.22 transfusions per cycle were costed | 8.44 |
|  | Outpatient appointment with haematologist | After cycle one, patients have an outpatient appointment halfway through the treatment course and another at the end. Average treatment length is 3.5 cycles (excluding cycle one), so 0.57 appointments per cycle were costed | 80.95 |
|  | Registrar appointment prior to treatment commencing | One appointment per cycle | 90.72 |
|  | Complications (Comparator A/B/C, first-line treatment) | See **Appendix Six** for more details of complication rates | 1006.85 |
|  | Complications (Comparator A/B/C, second-line treatment) | See **Appendix Six** for more details of complication rates | 2013.69 |
|  | Complications (Intervention 1/2) | See **Appendix Six** for more details of complication rates | 1006.85 |
|  |  | **TOTAL (Comparator A/B/C, first-line)** | **4427.32** |
|  |  | **TOTAL (Comparator A/B/C, second-line)** | **5434.16** |
|  |  | **TOTAL (Intervention 1/2)** | **4427.32** |
| Ofa treatment (first-line, first cycle) | Genetic test (Comparator A/B only) | - | 281.08 |
|  | Genomic test (Intervention 2 only) | - | 319.23 |
|  | Ofa treatment | - | 11876.84 |
|  | Hydrocortisone treatment | 100mg daily for 28 days | 30.24 |
|  | Chlorpheniramine treatment | 30mg daily for 28 days | 1.65 |
|  | Paracetamol | 1g daily for 28 days | 0.32 |
|  | FBC | One test per cycle | 3.01 |
|  | U&E | One test per cycle | 1.25 |
|  | LFT | One test per cycle | 1.25 |
|  | CT scan | One scan per cycle | 106.32 |
|  | Blood transfusion | 10% of patients have one transfusion over all cycles of Ofa treatment. Average treatment length is 5.8 cycles, so 0.17 transfusions per cycle were costed | 6.63 |
|  | Outpatient appointment with haematologist | One appointment per cycle | 142.71 |
|  | Registrar appointment prior to treatment commencing | One appointment per cycle | 90.72 |
|  | Complications | See **Appendix Six** for more details of complication rates | 255.93 |
|  |  | **TOTAL (Comparator A/B)** | **12797.93** |
|  |  | **TOTAL (Intervention 2)** | **12836.08** |
| Ofa treatment (first-line, second cycle) | Ofa treatment | - | 14942.86 |
|  | Hydrocortisone treatment | 100mg daily for 28 days | 30.24 |
|  | Chlorpheniramine treatment | 30mg daily for 28 days | 1.65 |
|  | Paracetamol | 1g daily for 28 days | 0.32 |
|  | FBC | One test per cycle | 3.01 |
|  | U&E | One test per cycle | 1.25 |
|  | LFT | One test per cycle | 1.25 |
|  | Blood transfusion | 10% of patients have one transfusion over all cycles of Ofa treatment. Average treatment length is 5.8 cycles, so 0.17 transfusions per cycle were costed | 6.63 |
|  | Registrar appointment prior to treatment commencing | One appointment per cycle | 90.72 |
|  | Complications | See **Appendix Six** for more details of complication rates | 255.93 |
|  |  | **TOTAL** | **15333.84** |
| Ofa treatment (first-line, subsequent cycles) | Ofa treatment | - | 3992.11 |
|  | FBC | One test per cycle | 3.01 |
|  | U&E | One test per cycle | 1.25 |
|  | LFT | One test per cycle | 1.25 |
|  | CT scan | One scan is given at the end of the full course of Ofa treatment. Average treatment length is 3.8 cycles (excluding cycles one and two), so 0.27 scans per cycle were costed | 28.27 |
|  | Blood transfusion | 10% of patients have one transfusion over all cycles of Ofa treatment. Average treatment length is 5.8 cycles, so 0.17 transfusions per cycle were costed | 6.63 |
|  | Outpatient appointment with haematologist | After cycles one and two, patients have an outpatient appointment halfway through the treatment course and another at the end. Average treatment length is 3.8 cycles (excluding cycles one and two), so 0.53 appointments per cycle were costed | 75.88 |
|  | Registrar appointment prior to treatment commencing | One appointment per cycle | 90.72 |
|  | Complications | See **Appendix Six** for more details of complication rates | 255.93 |
|  |  | **TOTAL** | **4455.03** |
| Ofa treatment (second-line or refractory, first cycle) | Ofa treatment | - | 11876.84 |
|  | Genetic test (Comparator A/B only) | - | 281.08 |
|  | Hydrocortisone treatment | 100mg daily for 28 days | 30.24 |
|  | Chlorpheniramine treatment | 30mg daily for 28 days | 1.65 |
|  | Paracetamol | 1g daily for 28 days | 0.32 |
|  | FBC | One test per cycle | 3.01 |
|  | U&E | One test per cycle | 1.25 |
|  | LFT | One test per cycle | 1.25 |
|  | CT scan | One scan per cycle | 106.32 |
|  | Blood transfusion | 10% of patients have one transfusion over all cycles of Ofa treatment. Average treatment length is 5.8 cycles, so 0.17 transfusions per cycle were costed | 6.63 |
|  | Outpatient appointment with haematologist | One appointment per cycle | 142.71 |
|  | Registrar appointment prior to treatment commencing | One appointment per cycle | 90.72 |
|  | Complications | See **Appendix Six** for more details of complication rates | 511.87 |
|  |  | **TOTAL (Comparator A/B)** | **13053.87** |
|  |  | **TOTAL (Comparator C)** | **12772.79** |
| Ofa treatment (second-line or refractory, second cycle) | Ofa treatment | - | 14942.86 |
|  | Hydrocortisone treatment | 100mg daily for 28 days | 30.24 |
|  | Chlorpheniramine treatment | 30mg daily for 28 days | 1.65 |
|  | Paracetamol | 1g daily for 28 days | 0.32 |
|  | FBC | One test per cycle | 3.01 |
|  | U&E | One test per cycle | 1.25 |
|  | LFT | One test per cycle | 1.25 |
|  | Blood transfusion | 10% of patients have one transfusion over all cycles of Ofa treatment. Average treatment length is 5.8 cycles, so 0.17 transfusions per cycle were costed | 6.63 |
|  | Registrar appointment prior to treatment commencing | One appointment per cycle | 90.72 |
|  | Complications | See **Appendix Six** for more details of complication rates | 511.87 |
|  |  | **TOTAL** | **15589.78** |
| Ofa treatment (second-line or refractory, subsequent cycles) | Ofa treatment | - | 3992.11 |
|  | FBC | One test per cycle | 3.01 |
|  | U&E | One test per cycle | 1.25 |
|  | LFT | One test per cycle | 1.25 |
|  | CT scan | One scan is given at the end of the full course of Ofa treatment. Average treatment length is 3.8 cycles (excluding cycles one and two), so 0.27 scans per cycle were costed | 28.27 |
|  | Blood transfusion | 10% of patients have one transfusion over all cycles of Ofa treatment. Average treatment length is 5.8 cycles, so 0.17 transfusions per cycle were costed | 6.63 |
|  | Outpatient appointment with haematologist | After cycles one and two, patients have an outpatient appointment halfway through the treatment course and another at the end. Average treatment length is 3.8 cycles (excluding cycles one and two), so 0.53 appointments per cycle were costed | 75.88 |
|  | Registrar appointment prior to treatment commencing | One appointment per cycle | 90.72 |
|  | Complications | See **Appendix Six** for more details of complication rates | 511.87 |
|  |  | **TOTAL** | **4710.97** |
| Ibrutinib treatment (first-line) | Genomic test | Patients undergo one test across the full course of IB treatment. Average treatment length is 9.3 cycles, so 0.10 tests per cycle were costed | 34.15 |
|  | IB treatment | - | 2472.10 |
|  | FBC | One test per cycle | 3.01 |
|  | U&E | One test per cycle | 1.25 |
|  | LFT | One test per cycle | 1.25 |
|  | CT scan | One scan is given at the start of treatment, and another after three months. Average treatment length is 9.3 cycles, so 0.21 scans per cycle were costed | 22.75 |
|  | IgV_H_ test | One test per cycle | 38.74 |
|  | Blood transfusion | 10% of patients have one transfusion over all cycles of IB treatment. Average treatment length is 9.3 cycles, so 0.11 transfusions per cycle were costed | 4.08 |
|  | Outpatient appointment with haematologist | Patients have three appointments across the whole treatment period. Average treatment length is 9.3 cycles, so 0.32 appointments per cycle were costed | 45.80 |
|  | Complications | See **Appendix Six** for more details of complication rates | 80.73 |
|  |  | **TOTAL** | **2703.84** |
| Ibrutinib treatment (second-line or refractory) | Genetic test (Comparator B only) | Patients undergo one test across the full course of IB treatment. Average treatment length is 9.3 cycles, so 0.10 tests per cycle were costed | 30.07 |
|  | Genomic test (Intervention 1/2 only) | As above - 0.10 tests per cycle were costed | 34.15 |
|  | IB treatment | - | 2472.10 |
|  | FBC | One test per cycle | 3.01 |
|  | U&E | One test per cycle | 1.25 |
|  | LFT | One test per cycle | 1.25 |
|  | CT scan | One scan is given at the start of treatment, and another after three months. Average treatment length is 9.3 cycles, so 0.21 scans per cycle were costed | 22.75 |
|  | IgV_H_ test | One test per cycle | 38.74 |
|  | Blood transfusion | 10% of patients have one transfusion over all cycles of IB treatment. Average treatment length is 9.3 cycles, so 0.11 transfusions per cycle were costed | 4.08 |
|  | Outpatient appointment with haematologist | Patients have three appointments across the whole treatment period. Average treatment length is 9.3 cycles, so 0.32 appointments per cycle were costed | 45.80 |
|  | Complications (Comparator B only) | See **Appendix Six** for more details of complication rates | 161.45 |
|  | Complications (Intervention 1/2 only) | See **Appendix Six** for more details of complication rates | 80.73 |
|  |  | **TOTAL (Comparator B)** | **2780.49** |
|  |  | **TOTAL (Intervention 1/2)** | **2703.84** |
| Remission following FCR treatment (all lines of treatment) | Co-trimoxazole treatment | 960mg three times a week. Given for three months (12 weeks) following last cycle of FCR treatment. 36 doses divided by the average number of 28 day cycles spent in remission following FCR treatment (56.3 cycles) so 0.64 doses per cycle | 0.12 |
|  | Fluconazole treatment | 50mg daily. Given for three months (12 weeks) following last cycle of FCR treatment. 84 doses divided by the average number of 28 day cycles spent in remission following FCR treatment (56.3 cycles) so 0.64 doses per cycle | 0.04 |
|  | Aciclovir treatment | 200mg three times a day. Given for three months (12 weeks) following last cycle of FCR treatment. 252 doses divided by the average number of 28 day cycles spent in remission following FCR treatment (56.3 cycles) so 4.48 doses per cycle | 0.14 |
|  | FBC | One test every three cycles | 1.00 |
|  | Outpatient appointment with haematologist | One appointment every three cycles | 47.57 |
|  | IVIG treatment | One infusion every three weeks in 10% of patients | 160.16 |
|  | Outpatient appointment with immunologist | One appointment every six months in 10% of patients | 3.25 |
|  |  | **TOTAL** | **212.29** |
| Remission following BR treatment (all lines of treatment) | FBC | One test every three cycles | 1.00 |
|  | Outpatient appointment with haematologist | One appointment every three cycles | 47.57 |
|  | IVIG treatment | One infusion every three weeks in 5% of patients | 80.08 |
|  | Outpatient appointment with immunologist | One appointment every six months in 5% of patients | 1.63 |
|  |  | **TOTAL** | **130.28** |
| Remission following Ofa treatment (all lines of treatment) | FBC | One test every three cycles | 1.00 |
|  | Outpatient appointment with haematologist | One appointment every three cycles | 47.57 |
|  |  | **TOTAL** | **48.57** |
| Undergoing BMT | BMT | One per cycle | **43723.96** |
| Remission following BMT | Outpatient appointment with haematologist | 1-2 appointments a week for the first eight weeks, then two appointments in the next four weeks, then one appointment every three weeks until 26 weeks. Assumed to be 19 appointments in total, spread over the average number of cycles spent in remission following BMT (51.3 cycles), so 0.37 appointments per cycle in total | 52.83 |
|  | Outpatient appointment with haematology nurse | As above - 0.37 appointments per cycle in total | 33.58 |
|  | Telephone consultation with haematology nurse | One appointment per week | 60.77 |
|  | CT scan | One scan is given after three months. Average treatment length is 51.3 cycles, so 0.02 scans per cycle were costed | 2.07 |
|  | Lung function test | 4-6 tests required over course of entire remission period (51.3 cycles) so costed five tests at 0.10 per cycle | 3.96 |
|  | Diagnostic bone marrow extraction | Three tests required over course of entire remission period (51.3 cycles) so 0.06 per cycle | 28.20 |
|  | IgV_H_ test | Three tests required over course of entire remission period (51.3 cycles) so 0.06 per cycle | 2.26 |
|  | Thyroid function test | Five tests required over course of entire remission period (51.3 cycles) so 0.10 per cycle | 0.12 |
|  | Immunoglobulin test | 6-7 tests required over course of entire remission period (51.3 cycles) so costed 6.5 tests at 0.13 per cycle | 0.38 |
|  | IVIG treatment | 5% of patients require one course of treatment, spread over the entire remission period (51.3 cycles) so 0.02 courses per cycle in total | 1.17 |
|  | FBC | Six tests required over course of entire remission period (51.3 cycles) so 0.12 per cycle | 0.35 |
|  | Donor lymphocyte infusion | 25% of patients require infusions. Marks *et al.* [35] report that 130 infusions were required across 81 patients. This figure was divided by the average number of cycles spent in remission following BMT, hence 0.03 infusions per cycle | 2.99 |
|  | Blood and platelet support | 15% of patients require transfusions, with 14 transfusions required on average over the three-month period following BMT. This figure was divided by the average number of cycles spent in remission following BMT hence 0.27 transfusions per cycle | 15.63 |
|  | Complications | See **Appendix Six** for more details of complication rates | 28.72 |
|  |  | **TOTAL** | **233.04** |
| BSC | Fluconazole treatment | 50mg daily in 20% of patients | 0.15 |
|  | Aciclovir treatment | 200mg three times a day in 20% of patients | 0.52 |
|  | IVIG treatment | One infusion every three weeks in 20% of patients | 320.33 |
|  | FBC | One test per cycle | 3.01 |
|  | Outpatient appointment with haematologist | One appointment per cycle | 142.71 |
|  | Blood transfusion | One transfusion every five weeks in 50% of patients | 152.75 |
|  | Inpatient stay | One appointment every 2-3 months (assumed to be every 2.5 months) | 1030.86 |
|  |  | **TOTAL** | **1650.33** |

BMT = bone marrow transplant; BR = bendamustine and rituximab; BSC = best supportive care; CT = computerised tomography; FBC = full blood count; FCR = fludarabine, cyclophosphamide and rituximab; GCSF = granulocyte colony-stimulating factor; IB = ibrutinib; IgV_H_ = immunoglobulin heavy chain variable region; IVIG = IV immunoglobulin treatment; LFT = liver function test; mcg = micrograms; mg = milligrams; Ofa = ofatumumab; U&E = urea and electrolytes test. ^a^ All chemotherapy costs include the cost of chemotherapy drugs, pharmacy costs and also the costs of treatment delivery. ^b^ A number of the calculations in this table incorporate information on the average number of cycles of each type of chemotherapy treatment. For further information on these calculations, please see **Appendix Six**.

# Appendix Eleven: Literature review to identify CLL utility values

The aim of this literature review was to identify references reporting utility values for disease states that could be experienced by chronic lymphocytic leukaemia (CLL) patients. An earlier study conducted a literature review with a similar objective, identifying a number of utility values with potential relevance for this analysis [36]. This earlier literature review considered all references published on or before 2^nd^ June 2010. To ensure that this analysis made use of all relevant utility information in this context, the same search syntax was applied from 3^rd^ June 2010 until 13^th^ June 2014.

The search strategies were described in detail in the previous study. In brief, the strategies combined multiple keyword variations using the logical operator ‘OR’ to identify studies reporting utility values for health states or toxicities associated with CLL and CLL treatment, or cost-effectiveness analyses, health technology assessment reports and review articles potentially reporting relevant utilities. Only English language studies were considered. Literature searches were undertaken in Embase, Medline and Medline In-Process, Econlit, and The UK National Health Service (NHS) Economic Evaluation Database. The reference lists of included articles were manually reviewed to check for additional studies that might also meet the inclusion criteria. In addition, a manual search of the NICE website was undertaken to identify relevant single technology appraisals.

The search results are described in the following table:

| **Database** | **Date** | **References identified** | **After removing duplicates** | **After reviewing titles** | **After reviewing abstracts** | **Articles evaluated, after discarding irrelevant and unavailable articles, and adding ‘other’ references** |
| --- | --- | --- | --- | --- | --- | --- |
| Embase | 13/06/14 | 463 | 452 | 159 | 26 | 3 |
| Medline | 13/06/14 | 124 | 29 | 10 | 3 | 0 |
| Econlit | 13/06/14 | 9 | 8 | 0 | 0 | 0 |
| NHS EED | 13/06/14 | 14 | 1 | 0 | 0 | 0 |
| Other | - | - | - | - | - | 4 |
| **Total** |  | **610** | **490** | **169** | **29** | **7** |
| **References not retained** |  |  | **120** | **321** | **140** | **24** |

NHS EED = NHS economic evaluation database.

A large number of those studies initially identified were not retained in the final count because these studies were all economic evaluations that used similar data sources for the utility weights. In addition, a number of the ‘Other’ references included in the final count were not identified in the specified databases because they were submitted as evidence to NICE instead of being published in a peer-reviewed journal.

After removing duplicates, reviewing titles, abstracts and full articles, and adding references from other sources, seven studies were taken forward [36-42]. This utility data was combined with that identified in the earlier review, and the most appropriate utility estimates were selected for use in the modelling process.

# Appendix Twelve: CLL utility values

Data on the utilities attached to health states experienced by CLL patients was extracted from the literature, as described in **Appendix Eleven**, and the most appropriate estimates were selected for use in the model. Sources which used preference-based approaches to estimate utility values for CLL health states using general population samples were favoured, as per NICE guidance [43]. The utility values that were selected were based on general health states (e.g. receiving a specific line of treatment, being in remission), and were not linked to specific treatments, as such data were not available in the literature. These utilities were then adjusted to reflect the complication rate in each health state (see **Appendix Six** for more information on complication rates), using information on the average disutility associated with a grade three or four adverse event. The table below presents the utilities that were applied in the model and some notes on the selection process.

| **Disease state** | **Utility** | **Notes** | **Source** |
| --- | --- | --- | --- |
| Undergoing first-line treatment | 0.803 | No information was available on this utility in the literature. Beusterien *et al.* [40] report four utility values for patients undergoing first-line treatment, elicited from the general public using a standard gamble (SG) approach (complete response, partial response, stable disease and progressive disease). An unweighted average of these four values was used. | [40] |
| Undergoing second-line treatment | 0.710 | SG approach used to elicit preferences from general public. | [40] |
| Undergoing refractory treatment | 0.650 | SG approach used to elicit preferences from general public. | [40] |
| Undergoing BMT | 0.650 | No information was available on this utility in the literature. It was assumed that patients undergoing BMT would have the same utility as those undergoing refractory treatment. | [40] |
| In remission | 0.910 | SG approach used to elicit preferences from general public. This utility was also applied for patients receiving ibrutinib treatment (adjusted to reflect the complication rate) as these patients are assumed to be asymptomatic until they no longer respond. | [40] |
| Disutility associated with anaemia | -0.090 | SG approach used to elicit preferences from general public. | [40] |
| Disutility associated with pyrexia | -0.110 | SG approach used to elicit preferences from general public. | [40] |
| Disutility associated with pneumonia | -0.200 | SG approach used to elicit preferences from general public. | [40] |
| Disutility associated with grade 3/4 adverse event | -0.133 | Unweighted average calculated based on the disutilities associated with anaemia, pyrexia and pneumonia. | Calc |
| Receiving BSC | 0.680 | SG approach used to elicit preferences from general public. Napp *et al.* [36] adjust the Beusterien *et al.* [40] values to fit the decision context considered in a UK HTA. | [36, 40] |

BMT = bone marrow transplant; BSC = best supportive care; Calc = calculated; HTA = health technology assessment; SG = standard gamble.

# Appendix Thirteen: Parameter variations considered in the sensitivity and scenario analyses

Sensitivity analysis

| **Parameter** | **Value** | | | **Justification for low/high values** |
| --- | --- | --- | --- | --- |
|  | **Base** | **Low** | **High** |  |
| **Test costs** |  |  |  |  |
| Cost of genetic testing (£) | 281.08 | 140.54 | 421.62 | Assumed 50% variation |
| Cost of genomic testing (£) | 319.23 | 159.62 | 478.85 | Assumed 50% variation |
| Cost of a FBC (£) | 3.01 | 1.68 | 3.97 | Informed by the lower and upper quartiles in the NHS Reference Costs database [28] |
| Cost of a U&E test (£) | 1.25 | 0.82 | 1.43 | Informed by the lower and upper quartiles in the NHS Reference Costs database [28] |
| Cost of a LFT (£) | 1.25 | 0.82 | 1.43 | Informed by the lower and upper quartiles in the NHS Reference Costs database [28] |
| Cost of a CT scan (£) | 106.32 | 89.71 | 143.10 | Informed by the lower and upper quartiles in the NHS Reference Costs database [28] |
| Cost of an IgVH test (£) | 38.74 | 16.50 | 51.57 | Informed by the lower and upper quartiles in the NHS Reference Costs database [28] |
| Cost of a simple lung function test (£) | 40.61 | 29.69 | 37.34 | Informed by the lower and upper quartiles in the NHS Reference Costs database [28] |
| Cost of a bone marrow aspirate (£) | 482.48 | 313.39 | 590.59 | Informed by the lower and upper quartiles in the NHS Reference Costs database [28] |
| Cost of a thyroid function test (£) | 1.25 | 0.82 | 1.43 | Informed by the lower and upper quartiles in the NHS Reference Costs database [28] |
| Cost of an immunoglobulin test (£) | 3.01 | 1.68 | 3.97 | Informed by the lower and upper quartiles in the NHS Reference Costs database [28] |
| **Cost of chemotherapy treatment** |  |  |  |  |
| FCR treatment – first cycle (£) | 2,554.74 | 1,277.37 | 3,832.12 | Assumed 50% variation |
| FCR treatment – subsequent cycles (£) | 2,876.02 | 1,438.01 | 4,314.03 | Assumed 50% variation |
| BR treatment – first cycle (£) | 2,854.63 | 1,427.32 | 4,281.95 | Assumed 50% variation |
| BR treatment – subsequent cycles (£) | 3,165.66 | 1,582.83 | 4,748.49 | Assumed 50% variation |
| Ofatumumab treatment – first cycle (£) | 11,826.62 | 5,913.31 | 17,739.94 | Assumed 50% variation |
| Ofatumumab treatment – second cycle (£) | 14,892.64 | 7,446.32 | 22,338.96 | Assumed 50% variation |
| Ofatumumab treatment – subsequent cycles (£) | 3,941.89 | 1,970.95 | 5,912.84 | Assumed 50% variation |
| Ibrutinib treatment (£) | 2,472.10 | 1,236.05 | 3,708.15 | Assumed 50% variation |
| **Other drug costs** |  |  |  |  |
| Cost per 960mg dose of co-trimoxazole (£) | 0.19 | 0.10 | 0.29 | Assumed 50% variation |
| Cost per 50mg dose of flucanazole (£) | 0.03 | 0.01 | 0.04 | Assumed 50% variation |
| Cost per 200mg dose of aciclovir (£) | 0.03 | 0.02 | 0.05 | Assumed 50% variation |
| Cost per 300mcg injection of GCSF (£) | 55.78 | 27.89 | 83.66 | Assumed 50% variation |
| Cost per 100mg injection of hydrocortisone (£) | 1.08 | 0.54 | 1.62 | Assumed 50% variation |
| Cost per 30mg dose of chlorpheniramine (£) | 0.06 | 0.03 | 0.09 | Assumed 50% variation |
| Cost per 1g dose of paracetamol (£) | 0.01 | 0.01 | 0.02 | Assumed 50% variation |
| **Other costs** |  |  |  |  |
| Cost for IVIG treatment (£) | 1,201.23 | 600.62 | 1,801.85 | Assumed 50% variation |
| Cost for a haematology appointment (consultant) (£) | 142.71 | 97.36 | 171.55 | Informed by the lower and upper quartiles in the NHS Reference Costs database [28] |
| Cost for a haematology appointment (non-consultant) (£) | 90.72 | 53.1 | 111.75 | Informed by the lower and upper quartiles in the NHS Reference Costs database [28] |
| Cost for a telephone consultation with a haematology nurse (£) | 60.77 | 39.39 | 62.03 | Informed by the lower and upper quartiles in the NHS Reference Costs database [28] |
| Cost for an immunology appointment (consultant) (£) | 195.01 | 85.24 | 322.01 | Informed by the lower and upper quartiles in the NHS Reference Costs database [28] |
| Cost for an inpatient stay (£) | 2,577.16 | 1,708.39 | 3,110.34 | Informed by the lower and upper quartiles in the NHS Reference Costs database [28] |
| Cost to treat a complication arising from chemotherapy (£) | 2,776.70 | 1,388.35 | 4,165.04 | Assumed 50% variation |
| Cost per BMT (£) | 43,723.96 | 21,861.98 | 65,585.93 | Assumed 50% variation |
| Cost to treat a complication arising from BMT (£) | 1,842.66 | 921.33 | 2,763.99 | Assumed 50% variation |
| Cost per donor lymphocyte infusion (£) | 381.88 | 190.94 | 572.82 | Assumed 50% variation |
| **Transition probabilities** |  |  |  |  |
| Comparator A/B: proportion of patients without *TP53* mutations | 0.9323 | 0.8500 | 0.9500 | Assumption |
| Comparator A/B: proportion of patients receiving FCR as first-line treatment | 0.8000 | 0.7500 | 0.8500 | Assumption |
| Comparator A/B: proportion of patients receiving FCR as second-line treatment | 0.2000 | 0.1500 | 0.2500 | Assumption |
| Proportion of patients who move from second-line FCR/BR straight to BSC: no *TP53* mutation/FCR responder | 0.0500 | 0.0250 | 0.0750 | Assumption |
| Proportion of patients who move from first-line Ofa treatment straight to BSC: *TP53* mutation/FCR non-responder | 0.0500 | 0.0250 | 0.0750 | Assumption |
| 28-day TP of dying from CLL during first-line FCR treatment: no *TP53* mutation | 0.0027 | 0.0014 | 0.0041 | Assumed 50% variation |
| 28-day TP of no response when undergoing first-line FCR treatment: no *TP53* mutation | 0.0016 | 0.0008 | 0.0024 | Assumed 50% variation |
| 28-day TP of patients moving straight to refractory treatment as high risk genetic mutations emerge following first-line FCR treatment: no *TP53* mutation | 0.0006 | 0.0003 | 0.0009 | Assumed 50% variation |
| 28-day TP of dying from CLL during first-line BR treatment: no *TP53* mutation | 0.0027 | 0.0014 | 0.0041 | Assumed 50% variation |
| 28-day TP of no response when undergoing first-line BR treatment: no *TP53* mutation | 0.0016 | 0.0008 | 0.0024 | Assumed 50% variation |
| 28-day TP of patients moving straight to refractory treatment as high risk genetic mutations emerge following first-line BR treatment: no *TP53* mutation | 0.0006 | 0.0003 | 0.0009 | Assumed 50% variation |
| 28-day TP of dying from CLL during second-line FCR treatment: no *TP53* mutation | 0.0033 | 0.0016 | 0.0049 | Assumed 50% variation |
| 28-day TP of moving to refractory treatment following no response to first-line FCR treatment: no *TP53* mutation | 0.0049 | 0.0025 | 0.0074 | Assumed 50% variation |
| 28-day TP of moving to BSC following no response to first-line FCR treatment: no *TP53* mutation | 0.0003 | 0.0001 | 0.0004 | Assumed 50% variation |
| 28-day TP of dying from CLL during second-line BR treatment: no *TP53* mutation | 0.0178 | 0.0089 | 0.0266 | Assumed 50% variation |
| 28-day TP of no response when undergoing second-line BR treatment: no *TP53* mutation | 0.0049 | 0.0025 | 0.0074 | Assumed 50% variation |
| 28-day TP of moving to BSC following second-line BR treatment: no *TP53* mutation | 0.0003 | 0.0001 | 0.0004 | Assumed 50% variation |
| 28-day TP of dying from CLL during refractory Ofa treatment: no *TP53* mutation | 0.0288 | 0.0160 | 0.0416 | Low and high values based on the estimates from Byrd *et al.* [17] and Wierda *et al.* [18] that informed the base case TP |
| 28-day TP of moving to BSC following refractory Ofa treatment: no *TP53* mutation | 0.0201 | 0.0101 | 0.0302 | Assumed 50% variation |
| 28-day TP of undergoing BMT following refractory Ofa treatment: no *TP53* mutation | 0.0174 | 0.0087 | 0.0261 | Assumed 50% variation |
| 28-day TP of dying from CLL during BMT remission: no *TP53* mutation | 0.0082 | 0.0058 | 0.0121 | Informed by the 95% CIs reported by Dreger *et al.* [19] |
| 28-day TP of relapsing during BMT remission: no *TP53* mutation | 0.0097 | 0.0058 | 0.0148 | Informed by the 95% CIs reported by Dreger *et al.* [19] |
| 28-day TP of moving to second-line treatment when in remission following first-line FCR/BR treatment: no *TP53* mutation | 0.0084 | 0.0042 | 0.0126 | Assumed 50% variation |
| 28-day TP of moving to refractory treatment when in remission following second-line FCR/BR treatment: no *TP53* mutation | 0.0185 | 0.0092 | 0.0277 | Assumed 50% variation |
| 28-day TP of moving to BSC when in remission following refractory Ofa treatment: no *TP53* mutation | 0.0737 | 0.0369 | 0.1106 | Assumed 50% variation |
| 28-day TP of dying from CLL when receiving BSC: no *TP53* mutation | 0.0753 | 0.0377 | 0.1130 | Assumed 50% variation |
| 28-day TP of dying from CLL during first-line Ofa treatment: *TP53* mutation | 0.0288 | 0.0144 | 0.0432 | Assumed 50% variation |
| 28-day TP of no response when undergoing first-line Ofa treatment: *TP53* mutation | 0.0440 | 0.0220 | 0.0660 | Assumed 50% variation |
| 28-day TP of undergoing BMT following refractory Ofa treatment: *TP53* mutation | 0.0174 | 0.0087 | 0.0261 | Assumed 50% variation |
| 28-day TP of moving to BSC following first-line Ofa treatment: *TP53* mutation | 0.0023 | 0.0012 | 0.0035 | Assumed 50% variation |
| 28-day TP of dying from CLL during BMT remission: *TP53* mutation | 0.0082 | 0.0058 | 0.0121 | Informed by the 95% CIs reported by Dreger *et al.* [19] |
| 28-day TP of relapsing during BMT remission: *TP53* mutation | 0.0097 | 0.0058 | 0.0148 | Informed by the 95% CIs reported by Dreger *et al.* [19] |
| 28-day TP of dying from CLL during refractory Ofa treatment: *TP53* mutation | 0.0288 | 0.0160 | 0.0416 | Low and high values based on the estimates from Byrd *et al.* [17] and Wierda *et al.* [18] that informed the base case TP |
| 28-day TP of moving to BSC following refractory Ofa treatment: *TP53* mutation | 0.0463 | 0.0231 | 0.0694 | Assumed 50% variation |
| 28-day TP of moving to refractory Ofa treatment when in remission following first-line Ofa treatment: *TP53* mutation | 0.0737 | 0.0369 | 0.1106 | Assumed 50% variation |
| 28-day TP of moving to BSC when in remission following refractory Ofa treatment: *TP53* mutation | 0.0737 | 0.0369 | 0.1106 | Assumed 50% variation |
| 28-day TP of dying from CLL when receiving BSC: *TP53* mutation | 0.0753 | 0.0377 | 0.1130 | Assumed 50% variation |
| 28-day TP of dying from CLL during refractory IB treatment: no *TP53* mutation | 0.0032 | 0.0016 | 0.0048 | Assumed 50% variation |
| 28-day TP of moving to BSC following refractory IB treatment: no *TP53* mutation | 0.0043 | 0.0021 | 0.0064 | Assumed 50% variation |
| 28-day TP of dying from CLL during refractory IB treatment: *TP53* mutation | 0.0131 | 0.0066 | 0.0197 | Assumed 50% variation |
| 28-day TP of moving to BSC when in remission following refractory IB treatment: *TP53* mutation | 0.0223 | 0.0111 | 0.0334 | Assumed 50% variation |
| 28-day TP of dying from CLL during first-line FCR treatment: any *TP53* mutation status | 0.0035 | 0.0018 | 0.0053 | Assumed 50% variation |
| 28-day TP of no response when undergoing first-line FCR treatment: any *TP53* mutation status | 0.0026 | 0.0013 | 0.0038 | Assumed 50% variation |
| 28-day TP of dying from CLL during first-line BR treatment: any *TP53* mutation status | 0.0035 | 0.0018 | 0.0053 | Assumed 50% variation |
| 28-day TP of no response when undergoing first-line BR treatment: any *TP53* mutation status | 0.0026 | 0.0013 | 0.0038 | Assumed 50% variation |
| 28-day TP of dying from CLL during second-line FCR treatment: any *TP53* mutation status | 0.0041 | 0.0021 | 0.0062 | Assumed 50% variation |
| 28-day TP of moving to refractory treatment following no response to first-line FCR treatment: any *TP53* mutation status | 0.0055 | 0.0027 | 0.0082 | Assumed 50% variation |
| 28-day TP of moving to BSC following no response to first-line FCR treatment: any *TP53* mutation status | 0.0003 | 0.0001 | 0.0004 | Assumed 50% variation |
| 28-day TP of dying from CLL during second-line BR treatment: any *TP53* mutation status | 0.0186 | 0.0093 | 0.0280 | Assumed 50% variation |
| 28-day TP of no response when undergoing second-line BR treatment: any *TP53* mutation status | 0.0055 | 0.0027 | 0.0082 | Assumed 50% variation |
| 28-day TP of moving to BSC following second-line BR treatment: any *TP53* mutation status | 0.0003 | 0.0001 | 0.0004 | Assumed 50% variation |
| 28-day TP of dying from CLL during refractory Ofa treatment: any *TP53* mutation status | 0.0288 | 0.0160 | 0.0416 | Low and high values based on the estimates from Byrd *et al.* [17] and Wierda *et al.* [18] that informed the base case TP |
| 28-day TP of moving to BSC following refractory Ofa treatment: any *TP53* mutation status | 0.0250 | 0.0125 | 0.0375 | Assumed 50% variation |
| 28-day TP of undergoing BMT following refractory Ofa treatment: any *TP53* mutation status | 0.0174 | 0.0087 | 0.0261 | Assumed 50% variation |
| 28-day TP of dying from CLL during BMT remission: any *TP53* mutation status | 0.0082 | 0.0058 | 0.0121 | Informed by the 95% CIs reported by Dreger *et al.* [19] |
| 28-day TP of relapsing during BMT remission: any *TP53* mutation status | 0.0097 | 0.0058 | 0.0148 | Informed by the 95% CIs reported by Dreger *et al.* [19] |
| 28-day TP of moving to second-line treatment when in remission following first-line FCR/BR treatment: any *TP53* mutation status | 0.0093 | 0.0046 | 0.0139 | Assumed 50% variation |
| 28-day TP of moving to refractory treatment when in remission following second-line FCR/BR treatment: any *TP53* mutation status | 0.0218 | 0.0109 | 0.0327 | Assumed 50% variation |
| 28-day TP of moving to BSC when in remission following refractory Ofa treatment: any *TP53* mutation status | 0.0737 | 0.0369 | 0.1106 | Assumed 50% variation |
| 28-day TP of dying from CLL when receiving BSC: any *TP53* mutation status | 0.0753 | 0.0377 | 0.1130 | Assumed 50% variation |
| Intervention 1/2: proportion of patients who are FCR responders | 0.8333 | 0.4167 | 1.2500 | Assumed 50% variation |
| Intervention 1/2: proportion of patients who are correctly identified as FCR responders | 0.7455 | 0.3727 | 1.1182 | Assumed 50% variation |
| 28-day TP of dying from CLL during first-line FCR treatment: FCR responder, correctly identified | 0.0022 | 0.0011 | 0.0032 | Assumed 50% variation |
| 28-day TP of no response when undergoing first-line FCR treatment: FCR responder, correctly identified | 0.0018 | 0.0009 | 0.0026 | Assumed 50% variation |
| 28-day TP of patients moving straight to refractory treatment as high risk genetic mutations emerge following first-line FCR treatment: FCR responder, correctly identified | 0.0006 | 0.0003 | 0.0009 | Assumed 50% variation |
| 28-day TP of dying from CLL during first-line FCR treatment: FCR responder, incorrectly identified | 0.0244 | 0.0122 | 0.0366 | Assumed 50% variation |
| 28-day TP of no response when undergoing first-line FCR treatment: FCR responder, incorrectly identified | 0.0097 | 0.0049 | 0.0146 | Assumed 50% variation |
| 28-day TP of patients moving straight to refractory treatment as high risk genetic mutations emerge following first-line FCR treatment: FCR responder, incorrectly identified | 0.0006 | 0.0003 | 0.0009 | Assumed 50% variation |
| 28-day TP of dying from CLL during second-line BR treatment: FCR responder | 0.0128 | 0.0064 | 0.0192 | Assumed 50% variation |
| 28-day TP of no response when undergoing second-line BR treatment: FCR responder | 0.0028 | 0.0014 | 0.0042 | Assumed 50% variation |
| 28-day TP of moving to BSC following second-line BR treatment: FCR responder | 0.0012 | 0.0006 | 0.0018 | Assumed 50% variation |
| 28-day TP of dying from CLL during refractory IB treatment: FCR responder | 0.0032 | 0.0016 | 0.0048 | Assumed 50% variation |
| 28-day TP of moving to second-line treatment when in remission following first-line FCR treatment: FCR responder, correctly identified | 0.0070 | 0.0035 | 0.0105 | Assumed 50% variation |
| 28-day TP of moving to second-line treatment when in remission following first-line FCR treatment: FCR responder, incorrectly identified | 0.0361 | 0.0180 | 0.0541 | Assumed 50% variation |
| 28-day TP of moving to refractory treatment when in remission following second-line BR treatment: FCR responder | 0.0127 | 0.0064 | 0.0191 | Assumed 50% variation |
| 28-day TP of moving to BSC following refractory IB treatment: FCR responder | 0.0043 | 0.0021 | 0.0064 | Assumed 50% variation |
| 28-day TP of dying from CLL when receiving BSC: FCR responder | 0.0753 | 0.0377 | 0.1130 | Assumed 50% variation |
| 28-day TP of dying from CLL during first-line IB treatment: FCR non responder | 0.0025 | 0.0012 | 0.0037 | Assumed 50% variation |
| 28-day TP of moving to BSC following first-line IB treatment: FCR non-responder | 0.0041 | 0.0020 | 0.0061 | Assumed 50% variation |
| 28-day TP of dying from CLL when receiving BSC: FCR non-responder | 0.0753 | 0.0377 | 0.1130 | Assumed 50% variation |
| Multiplier for mortality rate improvement following FCR treatment in FCR responders | 0.2000 | 0.1000 | 0.3000 | Assumed 50% variation |
| Multiplier for time to new treatment improvement following FCR treatment in FCR responders | 0.2000 | 0.1000 | 0.3000 | Assumed 50% variation |
| Multiplier for non-response rate improvement following FCR treatment in FCR responders | 0.2000 | 0.1000 | 0.3000 | Assumed 50% variation |
| Multiplier for mortality rate improvement following BR treatment in FCR responders | 0.2000 | 0.1000 | 0.3000 | Assumed 50% variation |
| Multiplier for non-response rate improvement following BR treatment in FCR responders | 0.2000 | 0.1000 | 0.3000 | Assumed 50% variation |
| Multiplier for movement to BSC improvement following BR treatment in FCR responders | 0.2000 | 0.1000 | 0.3000 | Assumed 50% variation |
| Multiplier for P of relapse improvement following BR treatment in FCR responders | 0.2000 | 0.1000 | 0.3000 | Assumed 50% variation |
| **Frequency of complications** |  |  |  |  |
| Average number of complications experienced per cycle of first-line FCR treatment | 0.349 | 0.174 | 0.523 | Assumed 50% variation |
| Average number of complications experienced per cycle of second-line FCR treatment | 0.698 | 0.349 | 1.047 | Assumed 50% variation |
| Average number of complications experienced per cycle of first-line FCR treatment: FCR responder | 0.174 | 0.087 | 0.262 | Assumed 50% variation |
| Average number of complications experienced per cycle of first-line BR treatment | 0.363 | 0.181 | 0.544 | Assumed 50% variation |
| Average number of complications experienced per cycle of second-line BR treatment | 0.725 | 0.363 | 1.088 | Assumed 50% variation |
| Average number of complications experienced per cycle of second-line BR treatment: FCR responder | 0.363 | 0.181 | 0.544 | Assumed 50% variation |
| Average number of complications experienced per cycle of first-line Ofa treatment | 0.092 | 0.046 | 0.138 | Assumed 50% variation |
| Average number of complications experienced per cycle of second- or third-line Ofa treatment | 0.184 | 0.092 | 0.277 | Assumed 50% variation |
| Average number of complications experienced per cycle of refractory IB treatment | 0.058 | 0.029 | 0.087 | Assumed 50% variation |
| Average number of complications experienced per cycle of refractory IB treatment: FCR responder | 0.029 | 0.015 | 0.044 | Assumed 50% variation |
| Average number of complications experienced per cycle of first-line IB treatment: FCR non-responder | 0.029 | 0.015 | 0.044 | Assumed 50% variation |
| **Utilities** |  |  |  |  |
| Undergoing first-line treatment | 0.803 | 0.624 | 0.910 | Low value: average of values in Napp *et al.* [36] and Ferguson *et al.* [41]. High value: utility assumed to be the same as remission |
| Undergoing second line treatment | 0.710 | 0.680 | 0.750 | Informed by 95% CI in Beusterien *et al.* [40] |
| Undergoing refractory treatment | 0.650 | 0.600 | 0.690 | Informed by 95% CI in Beusterien *et al.* [40] |
| Undergoing BMT | 0.650 | 0.585 | 0.715 | Assumed 10% variation |
| In remission | 0.910 | 0.880 | 0.930 | Informed by 95% CI in Beusterien *et al.* [40] |
| Average disutility associated with a grade 3/4 adverse event | -0.133 | -0.173 | -0.100 | Informed by 95% CI in Beusterien *et al.* [40] |
| Receiving BSC | 0.680 | 0.640 | 0.720 | Informed by 95% CIs in Napp *et al.* [36] |
| **Other parameters** |  |  |  |  |
| Body surface area (m^2^) | 1.88 | 1.72 | 1.93 | Informed by estimates used in two NICE submissions [36, 44] |
| Discount rate: costs | 0.035 | 0 | 0.050 | Assumption |
| Discount rate: outcomes | 0.035 | 0 | 0.050 | Assumption |
| Number of units of blood received during blood transplant | 2.10 | 1.05 | 3.15 | Assumed 50% variation |
| Average number of cycles of Ofa treatment | 5.76 | 2.88 | 8.64 | Assumed 50% variation |
| Average number of cycles of IB treatment | 9.35 | 4.67 | 14.02 | Assumed 50% variation |
| Average number of cycles spent in remission following FCR treatment | 56.31 | 50.22 | 62.61 | Informed by 95% CI in Hallek *et al.* [6] |
| Average number of cycles spent in remission following BMT | 51.32 | 25.66 | 76.98 | Assumed 50% variation |
| Average number of cycles of FCR treatment (excluding the first cycle) | 4.20 | 2.10 | 6.30 | Assumed 50% variation |
| Average number of cycles of BR treatment (excluding the first cycle) | 3.53 | 1.76 | 5.29 | Assumed 50% variation |
| Increase in complications: first-line FCR treatment to second-line FCR treatment (multiplier) | 2.00 | 1.00 | 3.00 | Assumed 50% variation |
| Decrease in complications: first-line FCR treatment to first-line FCR treatment in FCR responders (multiplier) | 0.50 | 0.25 | 0.75 | Assumed 50% variation |
| Decrease in complications: second-line BR treatment to first-line BR treatment (multiplier) | 0.50 | 0.25 | 0.75 | Assumed 50% variation |
| Decrease in complications: second-line BR treatment to second-line BR treatment in FCR responders (multiplier) | 0.50 | 0.25 | 0.75 | Assumed 50% variation |
| Decrease in complications: second-line Ofa treatment to first-line Ofa treatment (multiplier) | 0.50 | 0.25 | 0.75 | Assumed 50% variation |
| Decrease in complications: refractory IB treatment to refractory IB treatment in FCR responders (multiplier) | 0.50 | 0.25 | 0.75 | Assumed 50% variation |
| Decrease in complications: refractory IB treatment to first-line IB treatment in FCR non-responders (multiplier) | 0.50 | 0.25 | 0.75 | Assumed 50% variation |

BMT = bone marrow transplant; BR = bendamustine and rituximab; CI = confidence interval; CT = computerised tomography; FBC = full blood count; FCR = fludarabine, cyclophosphamide and rituximab; GCSF = granulocyte colony-stimulating factor; IB = ibrutinib; IgV_H_ = immunoglobulin variable region; IVIG = IV immunoglobulin; LFT = liver function test; NICE = National Institute for Health and Care Excellence; Ofa = ofatumumab; TP = transition probability; U&E = urea and electrolytes.

Scenario analysis

In addition to the parameter variations considered within the univariate sensitivity analysis, several scenario analyses were conducted. These scenarios considered reductions in the cost of ibrutinib treatment (by 50%, 67% and 75%) as well as the use of US costs (£63,011 per year), no genetic and genomic testing before subsequent lines of treatment, and variations in the assumed improved treatment effects applied following genomic testing (from a 20% improvement in the base case to 40% and 60%). Different starting ages for patients in the model were also considered (25 and 85), as well as the use of data from the alternative genomic testing model which excluded information on the presence of *SAMHD1* mutations (see **Appendix Three** for more details). To isolate the impact of use of ibrutinib treatment on the economic evaluation results a scenario was evaluated in which genetic testing parameters were applied instead of genomic testing parameters for Intervention One and Two. Finally, a scenario in which ibrutinib treatment was in widespread use was evaluated (i.e. Comparators A and C are no longer feasible strategies).

# Appendix Fourteen: Extending the economic evaluation to consider a societal perspective

Several references are available to guide analysts wishing to extend economic evaluations to a societal perspective [45-49]. There is a degree of consensus concerning which costs should be included in these analyses, with three main cost categories identified:

1. Productivity costs. These costs are incurred when the productivity of CLL patients is affected by illness, treatment, disability or premature death, and they fall into five sub-categories [50]:
   1. Short-term absenteeism due to CLL-related morbidity;
   2. Short-term presenteeism due to CLL-related morbidity;
   3. Long-term absenteeism due to CLL-related morbidity;
   4. Productivity losses due to CLL-related mortality;
   5. Productivity gains due to improved treatment.
2. Informal care costs. CLL patients frequently need informal care in addition to the care provided by healthcare professionals. This category captures the costs of the time invested in patient care by informal unpaid caregivers, such as family or friends [50].
3. Out of pocket costs. These costs can be incurred by both CLL patients and informal caregivers and fall into several sub-categories, including:
   1. Costs associated with receiving outpatient and inpatient care;
   2. Costs associated with day-to-day living;
   3. Other healthcare costs;
   4. Clothing and equipment costs.

The steps involved in estimating these costs are described below. All of the parameters that informed the societal cost calculations are reported in the table at the end of this appendix.

Productivity costs

Although many CLL patients are elderly, a proportion of this population are still employed, so productivity costs are still potentially important for this analysis. In addition to the costs that were captured in the five sub-categories below, it is also possible that compensation mechanisms and multiplier effects exist which act on these estimates. However, methods to account for these effects are currently underdeveloped [48, 51] so they are not included in the analysis.

*Short-term absenteeism due to CLL-related morbidity*

Short-term absences can occur due to time taken off work (or leisure) to attend routine healthcare appointments, or as a consequence of treatment. Such absences either result in lost earnings (due to time taken off work) or lost leisure time, which also has a monetary value. UK government data from 2013 indicates that the proportion of the population in employment is 80.9% (25-49 year olds), 67.4% (50-64 year olds) and 9.5% (65 years and older) [52]. As the starting age of patient cohorts in the Markov model is 65 years in the base case analysis, the estimate of 9.5% was applied. However, this proportion will diminish over time as patients age in the model and retire from employment. In the absence of data on the rate of change, this proportion was reduced by 20% a year such that no patients in the model were working five years after retirement (i.e. after reaching 70 years in age). This assumption was varied between one year and ten years in sensitivity analysis.

The costs associated with short-term absenteeism were then estimated for both employed and unemployed (or, more likely, retired) patients. For employed patients, data were available from a German study on events experienced by CLL patients which can lead to short-term absenteeism. This study reported that on average each year CLL patients take 7.6 sick days, have 14.7 physician contacts, and spend 8.9 days in hospital [53]. It was assumed that sick days and days in hospital could only be experienced during symptomatic states in the model i.e. not in the following states:

- General remission states;
- Bone marrow transplant (BMT – it was assumed that patients undergoing BMT would require the whole month off work as treatment is intensive);
- BMT remission states;
- Receiving best supportive care (BSC – it was assumed that patients would be off work permanently in this state);
- Receiving ibrutinib treatment (as patients receive ibrutinib until they become symptomatic).

The number of sick days or inpatient days per 28-day cycle in the model was therefore estimated to be 1.27. Physician contacts were assumed to require half a day away from employment, so the number of days of physician contacts per 28-day cycle was estimated to be 0.56. As these are only short-term absences from employment, either the human capital or friction cost approach can be used to value these absences [50]. Costs were therefore calculated using data on average UK hourly wage rates, adjusted for age, and assuming that a whole day at work is 7.5 hours [54].

Patients who are unemployed (or retired) were assumed to experience the same events as those in employment, in the same model states. However, the valuation of these events differed: minimum wage rates were applied to reflect the opportunity cost of the time of these patients (as per recently published cost of illness studies in heart disease and cancer [55-58]).

*Short-term presenteeism due to CLL-related morbidity*

Presenteeism captures the impact of reduced productivity during work or leisure time. There is little data available on the costs associated with presenteeism, either in CLL or cancer more generally. One literature review from 2008 found only one study reporting presenteeism rates for cancer [59]. This study reported that presenteeism in cancer is 8.45% over the course of a year, hence 2.4 days are lost per 28-day model cycle [60]. This percentage was applied during symptomatic model states as per the short-term absenteeism calculations, and average UK hourly wage rates (or minimum wage rates) were applied to value this time [54, 58]. This approach assumes that employees experiencing presenteeism are 0% effective during this time, which is a standard assumption in analyses of presenteeism. This assumption was tested in sensitivity analysis.

*Long-term absenteeism due to CLL-related morbidity*

No data was identified on long-term absenteeism in CLL. It was therefore assumed that this only occurs in the BSC state, until retirement age (assumed to be 65 years for both men and women). Information was extracted from the model on the average number of cycles spent in BSC for each comparator, and average UK hourly wage rates were applied to value this time [54]. Current guidance recommends that the friction cost approach is applied to value long-term absenteeism [49], so only absences up to 90 days were included in calculations. As the starting age of patients in the model is 65 years, there are no costs associated with long-term absenteeism in the base case analysis. However, starting ages of 25 years and 45 years were evaluated in a sensitivity analysis and in these scenarios a proportion of the patient cohort experienced long-term absenteeism.

*Productivity losses due to CLL-related mortality*

Information was extracted from the model on the number of deaths due to CLL in each cycle. This data was combined with information on retirement age to calculate working years lost to CLL, which were then valued using average UK hourly wage rates [54]. Again, there are no costs associated with productivity losses in the base case analysis due to the assumed starting age of 65 years.

*Productivity gains due to improved treatment*

Productivity gains due to improved treatment reflect additional healthy time used for both work and leisure. It has been noted previously that the value of additional healthy time used for leisure is probably captured in the utility scores for health states [45]. Capturing the value of additional healthy time used for work is more problematic, and primarily depends on whether patients considered this when providing utility data. No information is available in the studies from which utility data was extracted for the cost-utility analysis on whether patients were asked to consider the implications of their choices for their ability to work [40]. It is therefore unclear whether the value of additional healthy time used for work has already been captured. A number of papers suggest that productivity effects mainly impact on costs rather than outcomes, but evidence is generally lacking on this effect [61-63]. For the purposes of this analysis it was assumed that any productivity gains due to improved treatment were captured by the utility scores. Any missed productivity gains are likely to be small in size as many patients are either retired or approaching retirement.

Informal care costs

CLL patients frequently need a large amount of informal care in addition to the care provided by healthcare professionals. Information on the time costs of informal caregiving were extracted from a study which estimated the economic burden of blood disorders and blood cancers across the European Union (EU) [64]. This study provided information on the number of hours of informal care provided to two patient groups. The first group was patients dying with blood cancer. For the purposes of this analysis, these patients were assumed to be those receiving BSC. The second patient group was patients severely hampered by blood cancer, who were assumed to be those in any other symptomatic health state. In both cases, information was available on the number of hours of care provided by both employed and non-employed informal caregivers. Information on average UK hourly wage rates (or minimum wage rates) was applied to value this time and costs per 28-day model cycle were calculated [54, 58].

Out of pocket costs

Out of pocket (OOP) costs can be incurred by both CLL patients and informal caregivers.

*Patients*

Information on the OOP costs of patients was extracted from a report on the costs associated with cancer published by Macmillan, a UK cancer charity [65]. This report contained the results of a survey conducted amongst 1,600 UK cancer patients in 2012 which captured self-reported expenditure across a range of areas over a six-month period. Categories of expenditure included costs associated with receiving outpatient and inpatient care (e.g. travel costs), costs associated with day-to-day living (e.g. home help), other healthcare costs (e.g. over the counter medicines), and clothing and equipment costs. Information was extracted from the report on all categories of expenditure except for the costs of help around the home and personal care, to avoid double counting with informal care time costs. Average costs per 28-day cycle were then calculated. It should be noted that this publication did not report the costs associated with admission to a nursing home. These costs may be important to older CLL patients, but are not captured in this analysis.

*Informal caregivers*

Estimates of the non-time costs of informal caregivers were informed by an American study which provided this information for caregivers of patients with advanced terminal cancer and patients receiving continuing cancer care [66]. These estimates were provided as a proportion of total informal caregiver costs. This information was used to inflate the informal care costs extracted from the EU study [64] to generate estimates of non-time costs for informal caregivers for this analysis.

**Parameters used in the societal cost calculations**

| **Parameter** | **Category** | **Value ^a^** | **Source** |
| --- | --- | --- | --- |
| Proportion of the population in employment by age | 25-49 years | 80.9% | [52] |
|  | 50-64 years | 67.4% |  |
|  | 65 years and older | 9.5% |  |
| Rate at which the proportion of the population in employment decreases each year after the age of 65 years | - | 20% | Ass |
| Short-term absenteeism in CLL patients: mean annual values per CLL patient | Sick days | 7.6 | [53] |
|  | Physician contacts | 14.7 |  |
|  | Days in hospital | 8.9 |  |
| Length of a working day (hours) | - | 7.5 | Ass |
| Annual presenteeism rate | - | 8.45% | [60] |
| Proportion of time lost during presenteeism | - | 100% | Ass |
| Annual mean hours of informal care provided per blood cancer patient | Patients dying with blood cancer | 1,300 | [64] |
|  | Patients severely hampered by blood cancer | 34 |  |
| Mean out of pocket costs for patients per 28-day cycle | - | £59.64 | [65] |
| Mean out of pocket costs for informal caregivers per 28-day cycle | Patients dying with blood cancer | £81.20 | [64, 66] |
|  | Patients severely hampered by blood cancer | £2.09 |  |
| Retirement age (years) ^b^ | - | 65 |  |
| Median hourly earnings by age | 22-29 years | £10.58 | [54] |
|  | 30-39 years | £14.37 |  |
|  | 40-49 years | £14.63 |  |
|  | 50-59 years | £13.90 |  |
|  | 60 years and older | £12.12 |  |
| Minimum wage rate per hour | - | £6.31 | [58] |

Ass = assumption; CLL = chronic lymphocytic leukaemia. ^a^ All costs are for the year 2013; ^b^ Assumed to be the same for men and women.

Adapting the cost-effectiveness and cost-utility analysis to reflect a societal costing perspective

The additional costs that are relevant from a societal perspective were accrued by patients in model states as specified above, in addition to the costs that were accrued from a national health service perspective. All unit costs are reported in British Pounds for the year 2013. All of the parameters that were used in the societal cost calculations were varied in a univariate sensitivity analysis, with parameter ranges reflecting evidence from the original data source when this was available. In the absence of such evidence, parameters were varied by 50% above and below their base case value. Results were also calculated for different starting ages for patients in the model (25 and 45, with 25 reflecting the likely youngest age at presentation [67]). Probabilistic sensitivity analysis was used to further consider the impact of parameter uncertainty, and the results of these simulations were used to calculate cost-effectiveness acceptability curves.

# Appendix Fifteen: CHEERS checklist

| **Section/item** | **Item number** | **Recommendation** | **Reported on page number ^a^** | **Comments** |
| --- | --- | --- | --- | --- |
| **Title and abstract** |  |  |  |  |
| Title | 1 | Identify the study as an economic evaluation or use more specific terms such as “cost-effectiveness analysis”, and describe the interventions compared. | Page 1 | - |
| Abstract | 2 | Provide a structured summary of objectives, perspective, setting, methods (including study design and inputs), results (including base case and uncertainty analyses), and conclusions. | Page 2 | - |
| **Introduction** |  |  |  |  |
| Background and objectives | 3 | Provide an explicit statement of the broader context for the study. Present the study question and its relevance for health policy or practice decisions. | Pages 3, 4 | - |
| **Methods** |  |  |  |  |
| Target population and subgroups | 4 | Describe characteristics of the base case population and subgroups analysed, including why they were chosen. | Pages 4, 5 | See Sections 2.1 and 2.3. |
| Setting and location | 5 | State relevant aspects of the system(s) in which the decision(s) need(s) to be made. | Pages 4, 5 | See Section 2.2 and Appendix One. |
| Study perspective | 6 | Describe the perspective of the study and relate this to the costs being evaluated. | Pages 6, 7 | See section 2.6 for description of the base case perspective. See Section 2.10 and Appendix Fourteen for description of the use of a societal analytical perspective. |
| Comparators | 7 | Describe the interventions or strategies being compared and state why they were chosen. | Pages 4, 5 | See Section 2.2 and Appendix One. |
| Time horizon | 8 | State the time horizon(s) over which costs and consequences are being evaluated and say why appropriate. | Page 6 | See Section 2.8. |
| Discount rate | 9 | Report the choice of discount rate(s) used for costs and outcomes and say why appropriate. | Page 6 | See Section 2.8. |
| Choice of health outcomes | 10 | Describe what outcomes were used as the measure(s) of benefit in the evaluation and their relevance for the type of analysis performed. | Pages 4, 6 | See the opening paragraph of Section 2, and Section 2.7. |
| Measurement of effectiveness | 11 | *Single study-based estimates:* Describe fully the design features of the single effectiveness study and why the single study was a sufficient source of clinical effectiveness data.  *Synthesis-based estimates:* Describe fully the methods used for identification of included studies and synthesis of clinical effectiveness data. | Page 5 | See Section 2.4 and Appendix Three. |
| Measurement and valuation of preference based outcomes | 12 | If applicable, describe the population and methods used to elicit preferences for outcomes. | Page 6 | See Section 2.7 and Appendices Eleven and Twelve. |
| Estimating resources and costs | 13 | *Single study-based economic evaluation:* Describe approaches used to estimate resource use associated with the alternative interventions. Describe primary or secondary research methods for valuing each resource item in terms of its unit cost. Describe any adjustments made to approximate to opportunity costs.  *Model-based economic evaluation:* Describe approaches and data sources used to estimate resource use associated with model health states. Describe primary or secondary research methods for valuing each resource item in terms of its unit cost. Describe any adjustments made to approximate to opportunity costs. | Page 6 | See Section 2.6 and Appendices Five to Ten. |
| Currency, price date, and conversion | 14 | Report the dates of the estimated resource quantities and unit costs. Describe methods for adjusting estimated unit costs to the year of reported costs if necessary. Describe methods for converting costs into a common currency base and the exchange rate. | Page 6 | See Section 2.6. |
| Choice of model | 15 | Describe and give reasons for the specific type of decision-analytical model used. Providing a figure to show model structure is strongly recommended. | Page 4 | See Section 2.2, Figure 1, Table 1 and Appendix One. |
| Assumptions | 16 | Describe all structural or other assumptions underpinning the decision-analytical model. | Pages 4, 5 | See Sections 2.2 and 2.3. |
| Analytical methods | 17 | Describe all analytical methods supporting the evaluation. This could include methods for dealing with skewed, missing, or censored data; extrapolation methods; methods for pooling data; approaches to validate or make adjustments (such as half cycle corrections) to a model; and methods for handling population heterogeneity and uncertainty. | Pages 6,7 | See Sections 2.9, 2.9 and 2.10. |
| **Results** |  |  |  |  |
| Study parameters | 18 | Report the values, ranges, references, and, if used, probability distributions for all parameters. Report reasons or sources for distributions used to represent uncertainty where appropriate. Providing a table to show the input values is strongly recommended. | Pages 5, 6 | See Sections 2.4, 2.5 and 2.6, Appendices Three to Ten, and Appendices Twelve to Thirteen. |
| Incremental costs and outcomes | 19 | For each intervention, report mean values for the main categories of estimated costs and outcomes of interest, as well as mean differences between the comparator groups. If applicable, report incremental cost-effectiveness ratios. | Page 7 | See the opening paragraph of Section 2, and Table 4. |
| Characterising uncertainty | 20 | *Single study-based economic evaluation:* Describe the effects of sampling uncertainty for the estimated incremental cost and incremental effectiveness parameters, together with the impact of methodological assumptions (such as discount rate, study perspective).  *Model-based economic evaluation:* Describe the effects on the results of uncertainty for all input parameters, and uncertainty related to the structure of the model and assumptions. | Pages 7, 8, 9 | See Sections 3.1-3.4, Tables 5 and 6, Figure 2, and Appendices Sixteen to Seventeen. |
| Characterising heterogeneity | 21 | If applicable, report differences in costs, outcomes, or cost-effectiveness that can be explained by variations between subgroups of patients with different baseline characteristics or other observed variability in effects that are not reducible by more information. | Pages 8, 9 | See Sections 3.2 and 3.3. |
| **Discussion** |  |  |  |  |
| Study findings, limitations, generalisability, and current knowledge | 22 | Summarise key study findings and describe how they support the conclusions reached. Discuss limitations and the generalisability of the findings and how the findings fit with current knowledge. | Pages 9, 10 | - |
| **Other** |  |  |  |  |
| Source of funding | 23 | Describe how the study was funded and the role of the funder in the identification, design, conduct, and reporting of the analysis. Describe other non-monetary sources of support. | Page 12 | - |
| Conflicts of interest | 24 | Describe any potential for conflict of interest of study contributors in accordance with journal policy. In the absence of a journal policy, we recommend authors comply with International Committee of Medical Journal Editors recommendations. | Page 12 | - |

^a^ Page numbers refer to original submitted manuscript

# Appendix Sixteen: Parameter variations which change the base case analysis results

| **Parameter variation** | **Low/high value** | **Most cost-effective strategy (ICER) ^a^** | |
| --- | --- | --- | --- |
|  |  | **CEA** | **CUA** |
| Base case | - | Comparator A  (£7,903) | Comparator A  (£8,565) |
| Proportion of patients in Comparators A and B who do not have *TP53* mutations – varied between 85% and 95% (base case 93%) | Low | Comparator C  (£10,943) | Comparator C  (£12,447) |
|  | High | Comparator A  (£5,379) | Comparator A  (£5,852) |
| 28-day transition probability of dying from CLL during first-line FCR treatment: no *TP53* mutation – varied between 0.0014 and 0.0041 (base case 0.0027) | Low | Comparator A  (£7,316) | Comparator A  (£8,117) |
|  | High | Comparator C  (£5,855) | Comparator C  (£6,921) |
| 28-day transition probability of moving to second-line treatment when in remission following first-line FCR/BR treatment: no *TP53* mutation – varied between 0.0042 and 0.0126 (base case 0.0084) | Low | Comparator A  (£8,566) | Comparator A  (£9,626) |
|  | High | Comparator C  (£10,943) | Comparator C  (£12,447) |
| 28-day transition probability of moving to refractory treatment when in remission following second-line FCR/BR treatment: no *TP53* mutation – varied between 0.0092 and 0.0277 (base case 0.0185) | Low | Comparator A  (£10,016) | Comparator A  (£11,339) |
|  | High | Comparator A  (£29,590) | Comparator C  (£12,447) |
| 28-day transition probability of dying from CLL during first-line FCR treatment: any *TP53* mutation status – varied between 0.0018 and 0.0053 (base case 0.0035) | Low | Comparator C  (£7,245) | Comparator C  (£8,402) |
|  | High | Comparator A  (£7,671) | Comparator A  (£8,529) |
| 28-day transition probability of dying from CLL during second-line BR treatment: any *TP53* mutation status – varied between 0.0093 and 0.0280 (base case 0.0186) | Low | Comparator C  (£25,590) | Comparator A  (£12,298) |
|  | High | Comparator A  (£10,789) | Comparator A  (£12,113) |
| 28-day transition probability of moving to second-line treatment when in remission following first-line FCR/BR treatment: any *TP53* mutation status – varied between 0.0046 and 0.0139 (base case 0.0093) | Low | Comparator C  (£8,520) | Comparator C  (£9,586) |
|  | High | Comparator A  (£10,828) | Comparator A  (£12,298) |
| 28-day transition probability of moving to refractory treatment when in remission following second-line FCR/BR treatment: any *TP53* mutation status – varied between 0.0109 and 0.0327 (base case 0.0218) | Low | Comparator C  (£10,049) | Comparator C  (£11,392) |
|  | High | Comparator A  (£10,828) | Comparator A  (£12,298) |
| 28-day transition probability of moving to second-line treatment when in remission following first-line FCR treatment: FCR responder, correctly identified – varied between 0.0035 and 0.0105 (base case 0.0070) | Low | Intervention 2  (£21,053) | Intervention 2  (£19,248) |
|  | High | Comparator A  (£7,903) | Comparator A  (£8,565) |
| Cost discount rate – varied between 0% and 5% (base case 3.5%) | Low | Comparator A  (£9,698) | Comparator A  (£10,511) |
|  | High | Comparator B  (£29,666) | Comparator A  (£8,120) |
| Outcome discount rate – varied between 0% and 5% (base case 3.5%) | Low | Comparator B  (£22,968) | Comparator A  (£5,389) |
|  | High | Comparator A  (£9,580) | Comparator A  (£10,355) |

BR = bendamustine and rituximab; CEA = cost-effectiveness analysis; CLL = chronic lymphocytic leukaemia; CUA = cost-utility analysis; FCR = rituximab, cyclophosphamide and fludarabine; ICER – incremental cost-effectiveness ratio. ^a^ Parameter variations which change the base case analysis results are highlighted (Comparator B, Comparator C, Intervention 2).

# Appendix Seventeen: Scenario analysis results

Note: In all tables, the most cost-effective strategy at a threshold of £30,000 per LY/QALY gained is highlighted.

**Scenario One:** Changes in the cost of Ibrutinib treatment

**50% reduction**

| **Analysis** | **Comparator** | **Mean LYs/QALYs per patient** | **Mean costs per patient** | **ICER (excluding dominated strategies)** | **ICER (excluding extendedly dominated strategies)** |
| --- | --- | --- | --- | --- | --- |
| CEA | C | 6.37 | £69,704 | - | - |
|  | A | 6.61 | £71,576 | £7,903 | £7,903 |
|  | Int 2 | 6.65 | £74,460 | £82,818 | EXT.DOM |
|  | Int 1 | 7.45 | £86,992 | £15,592 | EXT.DOM |
|  | B | 7.63 | £87,027 | £188 | £15,130 |
| CUA | C | 5.60 | £69,704 | - | - |
|  | A | 5.82 | £71,576 | £8,565 | £8,565 |
|  | Int 2 | 5.93 | £74,460 | £25,285 | EXT.DOM |
|  | Int 1 | 6.67 | £86,992 | £17,027 | £18,135 |
|  | B | 6.44 | £87,027 | DOM | DOM |

**67% reduction**

| **Analysis** | **Comparator** | **Mean LYs/QALYs per patient** | **Mean costs per patient** | **ICER (excluding dominated strategies)** | **ICER (excluding extendedly dominated strategies)** |
| --- | --- | --- | --- | --- | --- |
| CEA | Int 2 | 6.65 | £68,684 | - | - |
|  | C | 6.37 | £69,704 | DOM | DOM |
|  | A | 6.61 | £71,576 | DOM | DOM |
|  | Int 1 | 7.45 | £76,294 | £9,468 | £9,468 |
|  | B | 7.63 | £80,135 | £21,032 | £21,032 |
| CUA | Int 2 | 5.93 | £68,684 | - | - |
|  | C | 5.60 | £69,704 | DOM | DOM |
|  | A | 5.82 | £71,576 | DOM | DOM |
|  | Int 1 | 6.67 | £76,294 | £10,340 | £10,340 |
|  | B | 6.44 | £80,135 | DOM | DOM |

**75% reduction**

| **Analysis** | **Comparator** | **Mean LYs/QALYs per patient** | **Mean costs per patient** | **ICER (excluding dominated strategies)** | **ICER (excluding extendedly dominated strategies)** |
| --- | --- | --- | --- | --- | --- |
| CEA | Int 2 | 6.65 | £65,796 | - | - |
|  | C | 6.37 | £69,704 | DOM | DOM |
|  | Int 1 | 7.45 | £70,945 | £6,406 | £6,406 |
|  | A | 6.61 | £71,576 | DOM | DOM |
|  | B | 7.63 | £76,689 | £31,453 | £31,453 |
| CUA | Int 2 | 5.93 | £65,796 | - | - |
|  | C | 5.60 | £69,704 | DOM | DOM |
|  | Int 1 | 6.67 | £70,945 | £6,996 | £6,996 |
|  | A | 5.82 | £71,576 | DOM | DOM |
|  | B | 6.44 | £76,689 | DOM | DOM |

**Increase to £63,011 per year (to match the cost of treatment in the USA)**

| **Analysis** | **Comparator** | **Mean LYs/QALYs per patient** | **Mean costs per patient** | **ICER (excluding dominated strategies)** | **ICER (excluding extendedly dominated strategies)** |
| --- | --- | --- | --- | --- | --- |
| CEA | C | 6.37 | £69,704 | - | - |
|  | A | 6.61 | £71,576 | £7,903 | £7,903 |
|  | Int 2 | 6.65 | £129,927 | £1,675,410 | EXT.DOM |
|  | B | 7.63 | £153,207 | £23,601 | £79,934 |
|  | Int 1 | 7.45 | £189,722 | DOM | DOM |
| CUA | C | 5.60 | £69,704 | - | - |
|  | A | 5.82 | £71,576 | £8,565 | £8,565 |
|  | Int 2 | 5.93 | £129,927 | £511,515 | EXT.DOM |
|  | B | 6.44 | £153,207 | £45,573 | £130,629 |
|  | Int 1 | 6.67 | £189,722 | £162,160 | £162,160 |

CEA = cost-effectiveness analysis; CUA = cost-utility analysis; DOM = dominated; EXT.DOM = extendedly dominated; ICER = incremental cost-effectiveness ratio; Int 1 = Intervention One; Int 2 = Intervention Two; LYs = life years; QALYs = quality-adjusted life years.

Comments: Comparator A is no longer the most cost-effective strategy in any scenario in which the cost of ibrutinib treatment is reduced. Instead, one of the strategies which uses ibrutinib treatment is always the most cost-effective option. In the cost-utility analysis (CUA), the most cost-effective strategy is always Intervention One. In the cost-effectiveness analysis (CEA), the most cost-effective strategy is Comparator B for 50% and 67% reductions in the cost of ibrutinib. For a 75% reduction in the cost, the most cost-effective strategy is Intervention One. An increase in the cost of ibrutinib to match the cost of treatment in the USA does not change the results, with Comparator A remaining the most cost-effective strategy in both the CEA and the CUA.

**Scenario Two:** No genetic or genomic testing before subsequent lines of treatment

| **Analysis** | **Comparator** | **Mean LYs/QALYs per patient** | **Mean costs per patient** | **ICER (excluding dominated strategies)** | **ICER (excluding extendedly dominated strategies)** |
| --- | --- | --- | --- | --- | --- |
| CEA | C | 6.37 | £69,704 | - | - |
|  | A | 6.61 | £71,358 | £6,984 | £6,984 |
|  | Int 2 | 6.65 | £91,226 | £570,451 | EXT.DOM |
|  | B | 7.63 | £107,021 | £16,013 | £34,921 |
|  | Int 1 | 7.45 | £118,524 | DOM | DOM |
| CUA | C | 5.60 | £69,704 | - | - |
|  | A | 5.82 | £71,358 | £7,568 | £7,568 |
|  | Int 2 | 5.93 | £91,226 | £174,163 | EXT.DOM |
|  | B | 6.44 | £107,021 | £30,921 | EXT.DOM |
|  | Int 1 | 6.67 | £118,524 | £51,085 | £55,484 |

CEA = cost-effectiveness analysis; CUA = cost-utility analysis; DOM = dominated; EXT.DOM = extendedly dominated; ICER = incremental cost-effectiveness ratio; Int 1 = Intervention One; Int 2 = Intervention Two; LYs = life years; QALYs = quality-adjusted life years.

Comments: Comparator A remains the most cost-effective strategy in both the CEA and CUA.

**Scenario Three:** Variations in the improved first-line and second-line treatment effect applied following genomic testing for patients identified as FCR responders

**Assumed improved treatment effect is 40% rather than 20%**

| **Analysis** | **Comparator** | **Mean LYs/QALYs per patient** | **Mean costs per patient** | **ICER (excluding dominated strategies)** | **ICER (excluding extendedly dominated strategies)** |
| --- | --- | --- | --- | --- | --- |
| CEA | C | 6.37 | £69,704 | - | - |
|  | A | 6.61 | £71,576 | £7,903 | £7,903 |
|  | Int 2 | 7.26 | £88,693 | £26,264 | £26,264 |
|  | B | 7.63 | £107,703 | £51,449 | EXT.DOM |
|  | Int 1 | 8.07 | £115,992 | £19,085 | £33,963 |
| CUA | C | 5.60 | £69,704 | - | - |
|  | A | 5.82 | £71,576 | £8,565 | £8,565 |
|  | Int 2 | 6.50 | £88,693 | £25,245 | £25,245 |
|  | B | 6.44 | £107,703 | DOM | DOM |
|  | Int 1 | 7.23 | £115,992 | £37,090 | £37,090 |

**Assumed improved treatment effect is 60% rather than 20%**

| **Analysis** | **Comparator** | **Mean LYs/QALYs per patient** | **Mean costs per patient** | **ICER (excluding dominated strategies)** | **ICER (excluding extendedly dominated strategies)** |
| --- | --- | --- | --- | --- | --- |
| CEA | C | 6.37 | £69,704 | - | - |
|  | A | 6.61 | £71,576 | £7,903 | £7,903 |
|  | Int 2 | 8.02 | £84,814 | £9,372 | £9,372 |
|  | B | 7.63 | £107,703 | DOM | DOM |
|  | Int 1 | 8.83 | £112,113 | £33,963 | £33,963 |
| CUA | C | 5.60 | £69,704 | - | - |
|  | A | 5.82 | £71,576 | £8,565 | £8,565 |
|  | Int 2 | 7.19 | £84,814 | £9,640 | £9,640 |
|  | B | 6.44 | £107,703 | DOM | DOM |
|  | Int 1 | 7.93 | £112,113 | £37,090 | £37,090 |

CEA = cost-effectiveness analysis; CUA = cost-utility analysis; DOM = dominated; EXT.DOM = extendedly dominated; ICER = incremental cost-effectiveness ratio; Int 1 = Intervention One; Int 2 = Intervention Two; LYs = life years; QALYs = quality-adjusted life years.

Comments: In both the CEA and CUA, the most cost-effective strategy changes from Comparator A to Intervention Two.

**Scenario Four:** Variations in patient starting ages

**Starting age is 25**

| **Analysis** | **Comparator** | **Mean LYs/QALYs per patient** | **Mean costs per patient** | **ICER (excluding dominated strategies)** | **ICER (excluding extendedly dominated strategies)** |
| --- | --- | --- | --- | --- | --- |
| CEA | C | 7.00 | £74,899 | - | - |
|  | A | 7.35 | £77,313 | £6,829 | £6,829 |
|  | Int 2 | 7.73 | £109,895 | £85,369 | EXT.DOM |
|  | B | 8.88 | £131,996 | £19,269 | £35,772 |
|  | Int 1 | 8.73 | £143,896 | DOM | DOM |
| CUA | C | 6.15 | £74,899 | - | - |
|  | A | 6.47 | £77,313 | £7,458 | £7,458 |
|  | Int 2 | 6.91 | £109,895 | £74,521 | EXT.DOM |
|  | B | 7.42 | £131,996 | £43,872 | EXT.DOM |
|  | Int 1 | 7.82 | £143,896 | £29,570 | £49,563 |

**Starting age is 85**

| **Analysis** | **Comparator** | **Mean LYs/QALYs per patient** | **Mean costs per patient** | **ICER (excluding dominated strategies)** | **ICER (excluding extendedly dominated strategies)** |
| --- | --- | --- | --- | --- | --- |
| CEA | C | 3.90 | £49,424 | - | - |
|  | A | 3.91 | £50,954 | £82,931 | EXT.DOM |
|  | Int 2 | 3.65 | £53,477 | DOM | DOM |
|  | B | 4.11 | £56,874 | £29,947 | £34,472 |
|  | Int 1 | 3.97 | £63,354 | DOM | DOM |
| CUA | C | 3.41 | £49,424 | - | - |
|  | A | 3.43 | £50,954 | £82,011 | EXT.DOM |
|  | Int 2 | 3.23 | £53,477 | DOM | DOM |
|  | B | 3.55 | £56,874 | £48,416 | £48,416 |
|  | Int 1 | 3.54 | £63,354 | DOM | DOM |

CEA = cost-effectiveness analysis; CUA = cost-utility analysis; DOM = dominated; EXT.DOM = extendedly dominated; ICER = incremental cost-effectiveness ratio; Int 1 = Intervention One; Int 2 = Intervention Two; LYs = life years; QALYs = quality-adjusted life years.

Comments: Comparator A remains the most cost-effective strategy for patients aged 25 in both the CEA and CUA. However, for patients aged 85, the most cost-effective strategy changes to Comparator C. In addition, if the NICE end-of-life threshold of £50,000 is applied, Comparator B becomes the most cost-effective strategy for patients aged 85 in both the CEA and CUA.

**Scenario Five:** Information on the presence of *SAMHD1* mutations excluded from the analysis

| **Analysis** | **Comparator** | **Mean LYs/QALYs per patient** | **Mean costs per patient** | **ICER (excluding dominated strategies)** | **ICER (excluding extendedly dominated strategies)** |
| --- | --- | --- | --- | --- | --- |
| CEA | C | 6.37 | £69,704 | - | - |
|  | A | 6.61 | £71,576 | £7,903 | £7,903 |
|  | Int 2 | 6.79 | £91,419 | £109,937 | EXT.DOM |
|  | B | 7.63 | £107,703 | £19,370 | £35.376 |
|  | Int 1 | 7.34 | £110,031 | DOM | DOM |
| CUA | C | 5.60 | £69,704 | - | - |
|  | A | 5.82 | £71,576 | £8,565 | £8,565 |
|  | Int 2 | 6.07 | £91,419 | £79,993 | EXT.DOM |
|  | B | 6.44 | £107,703 | £43,213 | EXT.DOM |
|  | Int 1 | 6.57 | £110,031 | £18,627 | £51,282 |

CEA = cost-effectiveness analysis; CUA = cost-utility analysis; DOM = dominated; EXT.DOM = extendedly dominated; ICER = incremental cost-effectiveness ratio; Int 1 = Intervention One; Int 2 = Intervention Two; LYs = life years; QALYs = quality-adjusted life years.

Comments: Comparator A remains the most cost-effective strategy in both the CEA and CUA.

**Scenario Six:** Ibrutinib treatment in widespread use

| **Analysis** | **Comparator** | **Mean LYs/QALYs per patient** | **Mean costs per patient** | **ICER (excluding dominated strategies)** | **ICER (excluding extendedly dominated strategies)** |
| --- | --- | --- | --- | --- | --- |
| CEA | Int 2 | 6.65 | £91,790 | - | - |
|  | B | 7.63 | £107,703 | £16,133 | £16,133 |
|  | Int 1 | 7.45 | £119,088 | DOM | DOM |
| CUA | Int 2 | 5.93 | £91,790 | - | - |
|  | B | 6.44 | £107,703 | £31,153 | £31,153 |
|  | Int 1 | 6.67 | £119,088 | £50,559 | £50,559 |

CEA = cost-effectiveness analysis; CUA = cost-utility analysis; DOM = dominated; EXT.DOM = extendedly dominated; ICER = incremental cost-effectiveness ratio; Int 1 = Intervention One; Int 2 = Intervention Two; LYs = life years; QALYs = quality-adjusted life years.

Comments: In this scenario, ibrutinib was assumed to be in widespread use and Comparators A and C were no longer feasible strategies. As such, Comparator B was the most cost-effective option in the CEA while Intervention Two was the most cost-effective option in the CUA. This indicates that the improved quality of life experienced by patients receiving ibrutinib treatment is an important driver of the economic evaluation results.

**Scenario Seven:** Genomic testing costs and parameters equalised with those of genetic testing

| **Analysis** | **Comparator** | **Mean LYs/QALYs per patient** | **Mean costs per patient** | **ICER (excluding dominated strategies)** | **ICER (excluding extendedly dominated strategies)** |
| --- | --- | --- | --- | --- | --- |
| CEA | C | 6.37 | £69,704 | - | - |
|  | A | 6.61 | £71,576 | £7,903 | £7,903 |
|  | Int 2 | 6.87 | £90,876 | £74,059 | EXT.DOM |
|  | Int 1 | 7.20 | £101,941 | £33,905 | EXT.DOM |
|  | B | 7.63 | £107,703 | £13,269 | £35.376 |
| CUA | C | 5.60 | £69,704 | - | - |
|  | A | 5.82 | £71,576 | £8,565 | £8,565 |
|  | Int 2 | 6.14 | £90,876 | £59,897 | EXT.DOM |
|  | Int 1 | 6.44 | £101,941 | £37,027 | £48,893 |
|  | B | 6.44 | £107,703 | £1,497,878 | £1,497,878 |

CEA = cost-effectiveness analysis; CUA = cost-utility analysis; DOM = dominated; EXT.DOM = extendedly dominated; ICER = incremental cost-effectiveness ratio; Int 1 = Intervention One; Int 2 = Intervention Two; LYs = life years; QALYs = quality-adjusted life years.

Comments: In this scenario, the cost of genomic testing was assumed to be the same as the cost of genetic testing, and the performance of genomic testing (in terms of the proportion of patients predicted to PFS positive, and the true positive rate) was equalised with the performance of genetic testing (see **Appendix Three** for more details). This scenario therefore strips out all changes in costs and outcomes associated with improved test performance, highlighting the changes that are due to the use of ibrutinib treatment. Comparator A remains the most cost-effective strategy in both the CEA and CUA, and changes in health outcomes and costs per patient are minimal for all strategies.

**Scenario Eight:** Use of new data on ibrutinib as initial therapy in CLL

| **Analysis** | **Comparator** | **Mean LYs/QALYs per patient** | **Mean costs per patient** | **ICER (excluding dominated strategies)** | **ICER (excluding extendedly dominated strategies)** |
| --- | --- | --- | --- | --- | --- |
| CEA | C | 6.37 | £69,704 | - | - |
|  | A | 6.61 | £71,576 | £7,903 | £7,903 |
|  | Int 2 | 6.65 | £91,790 | £580,390 | EXT.DOM |
|  | B | 7.63 | £107,703 | £16,133 | £35,376 |
|  | Int 1 | 7.51 | £120,841 | DOM | DOM |
| CUA | C | 5.60 | £69,704 | - | - |
|  | A | 5.82 | £71,576 | £8,565 | £8,565 |
|  | Int 2 | 5.93 | £91,790 | £177,198 | EXT.DOM |
|  | B | 6.44 | £107,703 | £31,153 | EXT.DOM |
|  | Int 1 | 6.72 | £120,841 | £50,559 | £54,868 |

CEA = cost-effectiveness analysis; CUA = cost-utility analysis; DOM = dominated; EXT.DOM = extendedly dominated; ICER = incremental cost-effectiveness ratio; Int 1 = Intervention One; Int 2 = Intervention Two; LYs = life years; QALYs = quality-adjusted life years.

Comments: After finalising the results of the analysis in this paper, new evidence on the use of ibrutinib as initial therapy in patients with CLL was published [68]. A final scenario considered the impact on the base case analysis of including this new evidence on PFS and OS. This new evidence only changed the results for Intervention One, increasing the number of life-years and QALYs per patient, but also increasing the mean cost per patient. Overall, Intervention One becomes slightly more cost-effective in the CUA, but with an ICER that is still higher than the NICE threshold of £30,000/QALY. Comparator A remains the most cost-effective strategy in both the CEA and CUA.

# Appendix Eighteen: Results from a societal perspective

The additional discounted societal costs for the entire patient cohort across the 30-year model timeframe are presented in the table on the following page for each comparator. The costs from a NHS perspective are also presented, along with combined costs across both analytical perspectives.

The highest societal costs are associated with Comparator A (current practice in hospitals that use genetic information to stratify patients by likely response to FCR treatment). These costs total £4,459 per patient, with informal care costs accounting for 48% of this figure. The lowest societal costs are associated with Intervention One (genomic testing followed by first-line ibrutinib treatment in likely FCR non-responders). These costs total £3,028 per patient, with informal care costs accounting for 49% of this figure. This difference likely arises because ibrutinib treatment leads to patients spending more time in asymptomatic health states, reducing the incidence of presenteeism and the associated costs for informal carers. The breakdown of societal costs across the different cost categories is relatively constant across the five strategies. In all cases, there are no costs associated with long-term absenteeism or mortality as the starting age of patients in the model is 65 years (i.e. retirement age).

Overall, the additional societal costs for the different strategies only represent a small increase in total costs from a NHS perspective. Societal costs as a proportion of total costs range from 2.5% (Intervention One) to 5.9% (Comparator A). Consequently, the cheapest (Comparator C - current practice with no genetic testing) and most expensive (Intervention One) strategies remain the same as when a NHS perspective is considered.

**Costs for each comparator from both a societal and NHS perspective**

| **Category** | **Comparator** | | | | | | | | | |
| --- | --- | --- | --- | --- | --- | --- | --- | --- | --- | --- |
|  | **A** | | **B** | | **C** | | **Int 1** | | **Int 2** | |
|  | **Costs** | **%** | **Costs** | **%** | **Costs** | **%** | **Costs** | **%** | **Costs** | **%** |
| Short-term absenteeism | £1,362,793 | 3% | £1,072,947 | 3% | £1,256,637 | 3% | £901,480 | 3% | £1,001,198 | 3% |
| Short-term presenteeism | £13,210,024 | 30% | £10,400,453 | 33% | £12,181,017 | 30% | £8,738,362 | 29% | £9,704,966 | 30% |
| Long-term absenteeism | £0 | 0% | £0 | 0% | £0 | 0% | £0 | 0% | £0 | 0% |
| Mortality costs | £0 | 0% | £0 | 0% | £0 | 0% | £0 | 0% | £0 | 0% |
| Informal care costs | £21,477,132 | 48% | £13,336,033 | 43% | £19,974,374 | 48% | £14,973,485 | 49% | £15,522,432 | 48% |
| OOP costs | £8,542,325 | 19% | £6,357,403 | 20% | £7,866,065 | 19% | £5,665,132 | 19% | £6,187,135 | 19% |
| **Total additional societal costs (whole cohort)** | **£44,592,274** | **-** | **£31,166,836** | **-** | **£41,278,092** | **-** | **£30,278,458** | **-** | **£32,415,731** | **-** |
| **Total additional societal costs (per patient)** | **£4,459** | **-** | **£3,117** | **-** | **£4,128** | **-** | **£3,028** | **-** | **£3,242** | **-** |
| **Total costs from NHS perspective (whole cohort)** | **£715,759,326** | **-** | **£1,077,033,723** | **-** | **£697,044,181** | **-** | **£1,190,882,282** | **-** | **£917,896,030** | **-** |
| **Total costs from NHS perspective (per patient)** | **£71,576** | **-** | **£107,703** | **-** | **£69,704** | **-** | **£119,088** | **-** | **£91,790** | **-** |
| **Total costs (whole cohort)** | **£760,351,600** | **-** | **£1,108,200,559** | **-** | **£738,322,273** | **-** | **£1,221,160,741** | **-** | **£950,311,761** | **-** |
| **Total costs (per patient)** | **£76,035** | **-** | **£110,820** | **-** | **£73,832** | **-** | **£122,116** | **-** | **£95,031** | **-** |

Int 1 = Intervention One; Int 2 = Intervention Two; NHS = National Health Service; OOP = out of pocket.

The results for the CEA and CUA from a societal perspective are presented in the table below. As the increase in mean costs per patient is small for each comparator, there is little change in the overall results. The same comparators are dominated (or extendedly dominated) in both the CEA and the CUA, and the ICERs are approximately the same, with small increases for Comparator A and small decreases for Comparator B and Intervention One.

**Results for the cost-effectiveness and cost-utility analyses from a societal perspective**

| **Analysis** | **Comparator** | **Mean LYs/QALYs per patient** | **Mean costs per patient** | **ICER (excluding dominated strategies)** | **ICER (excluding extendedly dominated strategies)** |
| --- | --- | --- | --- | --- | --- |
| CEA | C | 6.37 | £73,832 | - | - |
|  | A | 6.61 | £76,035 | £9,302 | £9,302 |
|  | Int 2 | 6.65 | £95,031 | £545,428 | EXT.DOM |
|  | B | 7.63 | £110,820 | £16,007 | £34,062 |
|  | Int 1 | 7.45 | £122,116 | DOM | DOM |
| CUA | C | 5.60 | £73,832 | - | - |
|  | A | 5.82 | £76,035 | £10,081 | £10,081 |
|  | Int 2 | 5.93 | £95,031 | £166,523 | EXT.DOM |
|  | B | 6.44 | £110,820 | £30,908 | EXT.DOM |
|  | Int 1 | 6.67 | £122,116 | £50,164 | £54,207 |

CEA = cost-effectiveness analysis; CUA = cost-utility analysis; DOM = dominated; EXT.DOM = extendedly dominated; ICER = incremental cost-effectiveness ratio; Int 1 = Intervention One; Int 2 = Intervention Two; LYs = life-years; QALYs = quality-adjusted life years.

Sensitivity analysis

As there are only small changes in the mean costs per patient for each comparator when a societal perspective is considered, the same parameter variations that change the most cost-effective strategy from Comparator A to an alternative strategy in the base case analysis are also important in this extended analysis. These results are therefore not presented. There were also few variations in the parameters that were used in the societal perspective that change the most cost-effective strategy from Comparator A, which was to be expected given the minimal contribution of societal costs to overall costs.

The only variation that did change the results was when different starting ages were considered for patients in the model. The results for the CEA and CUA from a societal perspective for patients aged 25 and 45 are presented in the following tables. The mean costs per patient increase considerably in both cases. For 25-year-old patients, the most expensive strategy is Comparator C at £707,752 per patient while the cheapest strategy is Intervention Two at £687,062 per patient. These large increases in cost arise because more CLL patients now die at a younger age, increasing the costs associated with premature mortality. Indeed, these costs represent 99% of all additional societal costs for all five strategies. The main consequence of these cost increases is that the most cost-effective strategy in the CEA changes from Comparator A to Comparator B (current practice in hospitals that use genetic information to stratify patients by likely response to FCR treatment, with ibrutinib given as refractory treatment for all patients) with an ICER of £2,943 per life-year gained. The most cost-effective strategy in the CUA also changes from Comparator A to Intervention One with an ICER of £19,933 per QALY gained. When a starting age of 45 years is considered, the mean costs per patient fall, but are still considerably higher than those incurred in the base case analysis. Comparator B is the most cost-effective strategy in both the CEA and the CUA for this patient group.

**Results for the cost-effectiveness and cost-utility analyses from a societal perspective for patients aged 25**

| **Analysis** | **Comparator** | **Mean LYs/QALYs per patient** | **Mean costs per patient** | **ICER (excluding dominated strategies)** | **ICER (excluding extendedly dominated strategies)** |
| --- | --- | --- | --- | --- | --- |
| CEA | Int 2 | 7.73 | £687,062 | - | - |
|  | B | 8.88 | £690,438 | £2,943 | £2,943 |
|  | A | 7.35 | £697,523 | DOM | DOM |
|  | Int 1 | 8.73 | £698,459 | DOM | DOM |
|  | C | 7.00 | £707,752 | DOM | DOM |
| CUA | Int 2 | 6.91 | £687,062 | - | - |
|  | B | 7.42 | £690,438 | £6,701 | £6,701 |
|  | A | 6.47 | £697,523 | DOM | DOM |
|  | Int 1 | 7.82 | £698,459 | £19,933 | £19,933 |
|  | C | 6.15 | £707,752 | DOM | DOM |

CEA = cost-effectiveness analysis; CUA = cost-utility analysis; DOM = dominated; EXT.DOM = extendedly dominated; ICER = incremental cost-effectiveness ratio; Int 1 = Intervention One; Int 2 = Intervention Two; LYs = life-years; QALYs = quality-adjusted life years.

**Results for the cost-effectiveness and cost-utility analyses from a societal perspective for patients aged 45**

| **Analysis** | **Comparator** | **Mean LYs/QALYs per patient** | **Mean costs per patient** | **ICER (excluding dominated strategies)** | **ICER (excluding extendedly dominated strategies)** |
| --- | --- | --- | --- | --- | --- |
| CEA | A | 7.25 | £345,301 | - | - |
|  | C | 6.91 | £352,471 | DOM | DOM |
|  | B | 8.70 | £354,766 | £6,530 | £6,530 |
|  | Int 2 | 7.57 | £365,165 | DOM | DOM |
|  | Int 1 | 8.54 | £370,966 | DOM | DOM |
| CUA | A | 6.38 | £345,301 | - | - |
|  | C | 6.07 | £352,471 | DOM | DOM |
|  | B | 7.27 | £354,766 | £10,616 | £10,616 |
|  | Int 2 | 6.77 | £365,165 | DOM | DOM |
|  | Int 1 | 7.65 | £370,966 | £43,272 | £43,272 |

CEA = cost-effectiveness analysis; CUA = cost-utility analysis; DOM = dominated; EXT.DOM = extendedly dominated; ICER = incremental cost-effectiveness ratio; Int 1 = Intervention One; Int 2 = Intervention Two; LYs = life-years; QALYs = quality-adjusted life years.

These results indicate that at younger ages, a strategy which includes ibrutinib treatment is likely to be the most cost-effective option from a societal perspective. This is because fewer patients die when undergoing ibrutinib treatment, which reduces mortality costs compared to strategies in which patients don’t receive ibrutinib.

A final point to note in relation to patient age is that the societal analysis did not include the costs associated with admission to a nursing home. The use of ibrutinib treatment in older patients may prevent such admissions as patients with effectively treated disease are better able to cope with other comorbidities. However, this effect is not captured in the model. If it were captured, it is possible that strategies which include ibrutinib treatment would also be cost-effective in older patients when a societal perspective is considered.

# References

1. Knight, S.J., et al., *Development and application of a targeted array test to diagnose and direct therapy in haematological cancers*. 2010, Health Innovation Challenge Fund.

2. Clifford, R., et al., *SAMHD1 is mutated recurrently in chronic lymphocytic leukemia and is involved in response to DNA damage.* Blood, 2014. **123**(7): p. 1021-31.

3. Clifford, R.M., et al., *Towards Response Prediction Using Integrated Genomics in Chronic Lymphocytic Leukaemia: Results on 250 First-Line FCR Treated Patients from UK Clinical Trials.* Blood, 2014. **124**(21): p. 1942-1942.

4. National Institute for Health and Care Excellence, *Appraisal consultation document – Ibrutinib for treating chronic lymphocytic leukaemia*. 2016.

5. Office for National Statistics, *National Life Tables, United Kingdom, 2010-2012*. 2013.

6. Hallek, M., et al., *Addition of rituximab to fludarabine and cyclophosphamide in patients with chronic lymphocytic leukaemia: a randomised, open-label, phase 3 trial.* Lancet, 2010. **376**(9747): p. 1164-74.

7. Shanafelt, T.D., et al., *Prospective Evaluation of Clonal Evolution During Long-Term Follow-Up of Patients With Untreated Early-Stage Chronic Lymphocytic Leukemia.* Journal of Clinical Oncology, 2006. **24**(28): p. 4634-4641.

8. Gunnarsson, R., et al., *Array-based genomic screening at diagnosis and during follow-up in chronic lymphocytic leukemia.* Blood, 2011. **96**(8): p. 1161-1169.

9. Cuneo, A., et al., *Appropriate use of bendamustine in first-line therapy of chronic lymphocytic leukemia. Recommendations from SIE, SIES, GITMO Group.* Leukemia Research, 2014(0).

10. Eichhorst, B., et al., *Chemoimmunotherapy With Fludarabine (F), Cyclophosphamide (C), and Rituximab (R) (FCR) Versus Bendamustine and Rituximab (BR) In Previously Untreated and Physically Fit Patients (pts) With Advanced Chronic Lymphocytic Leukemia (CLL): Results Of a Planned….* Blood, 2013. **122**(21): p. 526-526.

11. Fischer, K., et al., *Bendamustine in combination with rituximab for previously untreated patients with chronic lymphocytic leukemia: a multicenter phase II trial of the German Chronic Lymphocytic Leukemia Study Group.* J Clin Oncol, 2012. **30**(26): p. 3209-16.

12. Robak, T., et al., *Rituximab plus fludarabine and cyclophosphamide prolongs progression-free survival compared with fludarabine and cyclophosphamide alone in previously treated chronic lymphocytic leukemia.* J Clin Oncol, 2010. **28**(10): p. 1756-65.

13. Badoux, X.C., et al., *Fludarabine, cyclophosphamide, and rituximab chemoimmunotherapy is highly effective treatment for relapsed patients with CLL.* 2011. **117**(11): p. 3016-3024.

14. Eketorp Sylvan, S., et al., *Outcomes of patients with fludarabine-refractory chronic lymphocytic leukemia: a population-based study from a well-defined geographic region.* Leuk Lymphoma, 2014. **55**(8): p. 1774-80.

15. Tam, C.S., et al., *The natural history of fludarabine-refractory chronic lymphocytic leukemia patients who fail alemtuzumab or have bulky lymphadenopathy.* Leuk Lymphoma, 2007. **48**(10): p. 1931-9.

16. Fischer, K., et al., *Bendamustine Combined With Rituximab in Patients With Relapsed and/or Refractory Chronic Lymphocytic Leukemia: A Multicenter Phase II Trial of the German Chronic Lymphocytic Leukemia Study Group.* Journal of Clinical Oncology, 2011. **29**(26): p. 3559-3566.

17. Byrd, J.C., et al., *Ibrutinib versus ofatumumab in previously treated chronic lymphoid leukemia.* N Engl J Med, 2014. **371**(3): p. 213-23.

18. Wierda, W.G., et al., *Ofatumumab as single-agent CD20 immunotherapy in fludarabine-refractory chronic lymphocytic leukemia.* J Clin Oncol, 2010. **28**(10): p. 1749-55.

19. Dreger, P., et al., *Allogeneic stem cell transplantation provides durable disease control in poor-risk chronic lymphocytic leukemia: long-term clinical and MRD results of the German CLL Study Group CLL3X trial.* Blood, 2010. **116**(14): p. 2438-2447.

20. Wierda, W., et al., *Chemoimmunotherapy with fludarabine, cyclophosphamide, and rituximab for relapsed and refractory chronic lymphocytic leukemia.* J Clin Oncol, 2005. **23**(18): p. 4070-8.

21. National Institute for Health and Care Excellence, *Ofatumumab for the treatment of chronic lymphocytic leukaemia in patients who are refractory to fludarabine and alemtuzumab*. 2010.

22. Byrd, J.C., et al., *Three-year follow-up of treatment-naive and previously treated patients with CLL and SLL receiving single-agent ibrutinib.* Blood, 2015. **125**(16): p. 2497-506.

23. O'Brien, S., et al., *Ibrutinib as initial therapy for elderly patients with chronic lymphocytic leukaemia or small lymphocytic lymphoma: an open-label, multicentre, phase 1b/2 trial.* Lancet Oncology, 2014. **15**(1): p. 48-58.

24. Farooqui, M.Z., et al., *Ibrutinib for previously untreated and relapsed or refractory chronic lymphocytic leukaemia with TP53 aberrations: a phase 2, single-arm trial.* Lancet Oncol, 2015. **16**(2): p. 169-76.

25. Joint Formulary Committee, *British National Formulary (online)*. 2014, BMJ Group and Pharmaceutical Press: London.

26. Department of Health, *Drugs and pharmaceutical electronic market information (eMit)*. 2014.

27. Punekar, Y.S., A. Shukla, and H. Mullerova, *COPD management costs according to the frequency of COPD exacerbations in UK primary care.* Int J Chron Obstruct Pulmon Dis, 2014. **9**: p. 65-73.

28. Department of Health. *NHS Reference Costs 2012-2013*. 2014; Available from: https://www.gov.uk/government/collections/nhs-reference-costs#published-reference-costs.

29. Department of Health, *NHS Blood and Transplant Price List 2012/13*. 2013.

30. Hoyle M, et al., *Dasatinib, nilotinib and standard dose imatinib for the first-line treatment of chronic myeloid leukaemia: systematic reviews and economic analyses*. 2011: University of Exeter (Report for NICE).

31. Curtis, L., *Unit Costs of Health & Social Care*. 2014, Personal Social Services Research Unit, University of Kent.

32. Health and Social Care Information Centre, *Health Survey for England - 2012*. 2013.

33. Roche Products Ltd, *Rituximab for the treatment of relapsed or refractory chronic lymphocytic leukaemia*. 2009.

34. Oscier, D., et al., *Guidelines on the diagnosis, investigation and management of Chronic Lymphocytic Leukaemia*. 2012, British Committee for Standards in Haematology.

35. Marks, D.I., et al., *The toxicity and efficacy of donor lymphocyte infusions given after reduced-intensity conditioning allogeneic stem cell transplantation.* Blood, 2002. **100**(9): p. 3108-3114.

36. Napp Pharmaceuticals Ltd, *Bendamustine for the first-line treatment of chronic lymphocytic leukaemia (Binet stage B or C) in patients for whom fludarabine combination chemotherapy is not appropriate*. 2010.

37. Hancock, S., B. Wake, and C. Hyde, *Fludarabine as first line therapy for chronic lymphocytic leukaemia*. 2003, West Midlands Health Technology Assessment Collaboration.

38. Hoyle, M., et al., *Ofatumumab (Arzerra) for the treatment of chronic lymphocytic leukaemia in patients who are refractory to fludarabine and alemtuzumab: a critique of the submission from GSK* 2010, National Institute for Health and Care Excellence.

39. Kharfan-Dabaja, M.A., et al., *Comparing efficacy of reduced-toxicity allogeneic hematopoietic cell transplantation with conventional chemo-(immuno) therapy in patients with relapsed or refractory CLL: a Markov decision analysis.* Bone Marrow Transplant, 2012. **47**(9): p. 1164-70.

40. Beusterien, K.M., et al., *Population preference values for treatment outcomes in chronic lymphocytic leukaemia: a cross-sectional utility study.* Health Qual Life Outcomes, 2010. **8**: p. 50.

41. Ferguson, J., et al., *Health state preference study mapping the change over the course of the disease process in chronic lymphocytic leukaemia (CLL).* Value in Health, 2008. **11**(6): p. A485.

42. Tolley, K., et al., *Utility elicitation study in the UK general public for late-stage chronic lymphocytic leukaemia.* Eur J Health Econ, 2013. **14**(5): p. 749-59.

43. National Institute for Health and Care Excellence, *Guide to the methods of technology appraisal*. 2013.

44. Roche Products Ltd, *Rituximab for the first-line treatment of chronic lymphocytic leukaemia*. 2008.

45. Drummond, M.F., et al., *Methods for the Economic Evaluation of Health Care Programmes*. Third ed. 2005.

46. Drummond, M. and A. McGuire, *Economic Evaluation in Health Care: Merging Theory with Practice*. 2002: Oxford University Press.

47. McIntosh, E., et al., eds. *Applied Methods of Cost-benefit Analysis in Health Care*. 2010, Oxford University Press.

48. Krol, M. and W. Brouwer, *How to Estimate Productivity Costs in Economic Evaluations.* PharmacoEconomics, 2014. **32**(4): p. 335-344.

49. Tan, S.S., et al., *Update of the Dutch Manual for Costing in Economic Evaluations.* Int J Technol Assess Health Care, 2012. **28**(2): p. 152-8.

50. Krol, M., W. Brouwer, and F. Rutten, *Productivity costs in economic evaluations: past, present, future.* Pharmacoeconomics, 2013. **31**(7): p. 537-49.

51. Krol, M., et al., *Productivity cost calculations in health economic evaluations: correcting for compensation mechanisms and multiplier effects.* Soc Sci Med, 2012. **75**(11): p. 1981-8.

52. Department for Work & Pensions, *Older Workers Statistical Information Booklet 2013: Official Statistics*. 2013.

53. Blankart, C.R., et al., *Cost of illness and economic burden of chronic lymphocytic leukemia.* Orphanet Journal of Rare Diseases, 2013. **8**: p. 32-32.

54. Office for National Statistics, *Patterns of Pay: Estimates from the Annual Survey of Hours and Earnings, UK, 1997 to 2013*. 2014.

55. Luengo-Fernandez, R., et al., *Economic burden of cancer across the European Union: a population-based cost analysis.* Lancet Oncol, 2013. **14**(12): p. 1165-74.

56. Luengo‐Fernández, R., et al., *Cost of cardiovascular diseases in the United Kingdom.* Heart, 2006. **92**(10): p. 1384-1389.

57. Krol, M., *Productivity costs in economic evaluations*. 2012, Erasmus University Rotterdam,.

58. www.gov.uk. *National Minimum Wage rates*. 2015 22/06/2015]; Available from: https://www.gov.uk/national-minimum-wage-rates.

59. Schultz, A., C.-Y. Chen, and D. Edington, *The Cost and Impact of Health Conditions on Presenteeism to Employers.* PharmacoEconomics, 2009. **27**(5): p. 365-378.

60. Goetzel, R.Z., et al., *Health, absence, disability, and presenteeism cost estimates of certain physical and mental health conditions affecting U.S. employers.* J Occup Environ Med, 2004. **46**(4): p. 398-412.

61. Krol, M., W. Brouwer, and P. Sendi, *Productivity costs in health-state valuations : does explicit instruction matter?* Pharmacoeconomics, 2006. **24**(4): p. 401-14.

62. Krol, M., P. Sendi, and W. Brouwer, *Breaking the silence: exploring the potential effects of explicit instructions on incorporating income and leisure in TTO exercises.* Value Health, 2009. **12**(1): p. 172-80.

63. Tilling, C., et al., *Does the EQ-5D Reflect Lost Earnings?* PharmacoEconomics, 2012. **30**(1): p. 47-61.

64. Burns, R., R. Luengo-Fernandez, and J. Leal, *Economic Burden of Disorders of the Blood in Europe*, in *European Hematology Association 20th Congress*. 2015: Vienna, Austria.

65. Finney, A., et al., *Cancer's hidden price tag: Revealing the costs behind the illness*. 2013, Macmillan Cancer Support.

66. Van Houtven, C.H., et al., *Economic Burden for Informal Caregivers of Lung and Colorectal Cancer Patients.* The Oncologist, 2010. **15**(8): p. 883-893.

67. Oscier, D., et al., *Guidelines on the diagnosis and management of chronic lymphocytic leukaemia.* British Journal of Haematology, 2004. **125**(3): p. 294-317.

68. Burger, J.A., et al., *Ibrutinib as Initial Therapy for Patients with Chronic Lymphocytic Leukemia.* New England Journal of Medicine, 2015. **373**(25): p. 2425-2437.
